# Upregulation of cell cycle genes in head and neck cancer patients may be antagonized by erufosine’s down regulation of cell cycle processes in OSCC cells

**DOI:** 10.18632/oncotarget.23537

**Published:** 2017-12-20

**Authors:** Shariq S. Ansari, Ashwini K. Sharma, Michael Zepp, Elizabet Ivanova, Frank Bergmann, Rainer König, Martin R. Berger

**Affiliations:** ^1^ Toxicology and Chemotherapy Unit, German Cancer Research Center, Heidelberg, Germany; ^2^ Institute for Pharmacy and Molecular Biotechnology (IPMB) and BioQuant, Heidelberg University, Heidelberg, Germany; ^3^ Division of Theoretical Bioinformatics, German Cancer Research Center (DKFZ), Heidelberg, Germany; ^4^ Laboratory for Experimental Chemotherapy, Department of Pharmacology, Pharmacotherapy and Toxicology, Faculty of Pharmacy, Medical University of Sofia, Bulgaria; ^5^ Institute of Pathology, University Hospital Heidelberg, Heidelberg, Germany; ^6^ Integrated Research and Treatment Center Center for Sepsis Control and Care (CSCC), Jena University Hospital, Jena, Germany; ^7^ Network Modeling, Leibniz Institute for Natural Products Research and Infection Biology, Hans-Knöll-Institute, Jena, Germany

**Keywords:** erufosine, head and neck cancer, cyclins and CDKs, G2/M block, OSCC xenograft mouse model

## Abstract

The TCGA database was analyzed to identify deregulation of cell cycle genes across 24 cancer types and ensuing effects on patient survival. Pan-cancer analysis showed that head and neck squamous cell carcinoma (HNSCC) ranks amongst the top four cancers showing deregulated cell cycle genes. Also, the median gene expression of all CDKs and cyclins in HNSCC patient samples was higher than that of the global gene expression. This was verified by IHC staining of CCND1 from HNSCC patients. When evaluating the quartiles with highest and lowest expression, increased CCND1/CDK6 levels had negative implication on patient survival. In search for a drug, which may antagonize this tumor profile, the potential of the alkylphosphocholine erufosine was evaluated against cell lines of the HNSCC subtype, oral squamous cell carcinoma (OSCC) using *in-vitro* and *in-vivo* assays. Erufosine inhibited growth of OSCC cell lines concentration dependently. Initial microarray findings revealed that cyclins and CDKs were down-regulated concentration dependently upon exposure to erufosine and participated in negative enrichment of cell cycle processes. These findings, indicating a pan-cdk/cyclin inhibition by erufosine, were verified at both, mRNA and protein levels. Erufosine caused a G2/M block and inhibition of colony formation. Significant tumor growth retardation was seen upon treatment with erufosine in a xenograft model. For the decreased cyclin D1 and CDK 4/6 levels found in tumor tissue, these proteins can serve as biomarker for erufosine intervention. The findings demonstrate the potential of erufosine as cell cycle inhibitor in HNSCC treatment, alone or in combination with current therapeutic agents.

## INTRODUCTION

Cell cycle is a tightly regulated and integrated set of events, which ensures the cell to proliferate and grow by passing through the G1, S, G2 and M phases [1]. The core purpose of this regulated event is to make sure that DNA is duplicated during the S phase and equally distributed to the daughter cells [2]. The completion of different phases is regulated by the cyclin-dependent kinases (CDKs), which provide transition from one phase to another in a controlled fashion [3]. CDK activity entails binding of regulatory subunits, cyclins, that are synthesized and degraded at specific times during cell cycle progression, thus regulating kinase activity in a timely manner [4]. This progression has to go through several ‘cell cycle checkpoints’ that sense possible defects during DNA synthesis and chromosome segregation [4]. Components of the cell cycle machinery are frequently altered in human cancer. CDKs govern the initiation, progression, and completion of cell cycle events, and are therefore a major target for deregulation in cancer [5]. Over the last two decades, numerous CDK inhibitors have been developed as cancer therapeutics [6] and various clinical trials in several tumor types have been carried out, reinforcing CDKs/cyclins as potential targets for anticancer drug development [7, 8].

Squamous cell carcinoma of the head and neck (HNSCC) consists of a group of cancers that originate in the mucosal linings of the upper aero digestive tract and, in aggregate, represent the sixth leading cause of cancer worldwide [9–11]. Oral squamous cell carcinoma (OSCC) refers to the most common malignant tumor of the head and neck, constituting about 90% of all the reported HNSCC [12]. Oral cancer constitutes the cancers of the oral cavity (tongue, floor of mouth, hard palate, buccal mucosa, and alveolar ridges) and oropharynx (base of tongue, tonsils, and soft palate). The major risk factors for HNSCC include tobacco and alcohol consumption [13], with higher incidences in South East Asian countries when compared to the Western world [14]. Surgery, radiation and chemotherapy are the mainstay treatments for head and neck cancer [15]. Cisplatin is the chemotherapeutic agent of choice in the chemo radiotherapy regimen for treating advanced HNSCC [16, 17]. Also a combination regimen of radiotherapy and cetuximab is often used for treating selected patients [18, 19]. Despite these improvements, survival has not markedly improved because patients still frequently develop local-regional recurrences, distant metastasis and second primary tumors [20]. Thus, OSCC is still a challenging disease to treat in the field of head and neck cancer.

Ether lipids and their derivatives relating to platelet activating factor (PAF) and various alkyl-phospho-lipids (APL) have been used as anti-neoplastic agents as they induce apoptosis and decrease cell migration/invasion, leading to the inhibition of tumor and metastasis development [21]. The ether lipid analogue, erufosine (erucylphospho-N,N,N,-trimethyl-propylammonium, ErPC_3_) is an antineoplastic agent classified as a third generation alkylphosphocholine (APC) [22]. Due to the structure with 22-carbon long chain and the ω-9 cis-double bond, it forms lamellar instead of micellar structures in aqueous solutions, resulting in lack of hemolytic activity and hence can be intravenously administered [23]. Erufosine and other APC’s are known to stimulate normal hematopoiesis in bone marrow, which implies that they could be used in combination treatment regimens in order to ameliorate the toxicity of conventional cytotoxic agents [24]. The mode of action of APCs involves disturbance of phospholipid metabolism and of membrane lipid raft-mediated signaling [25]. APCs have shown significant cytotoxic and pro-apoptotic activities towards a number of tumors cell lines by modulating members of the JNK 1/2, Raf/MEK/ERK, and PI3K/Akt/mTOR signaling pathways and inhibiting cell division [26–28].

In the present study, we aimed to investigate the extent of deregulation of cell cycle genes across various cancer types with specific emphasis on head and neck cancer and to determine the effect of the deregulated genes on patient survival. We also report the potential of erufosine to cause downregulation of cyclins and CDks in human OSCC cell lines and describe this effect as negative gene enrichment of the cell cycle processes. As the changes in gene expression levels caused by erufosine correspond to the potential therapeutic targets in clinical settings, we extended the translational importance by characterizing erufosine’s anti-neoplastic activity *in-vitro* as well as by a xenograft mouse model.

## RESULTS

### Cell cycle genes are upregulated in head and neck cancer

**Figure 1:**
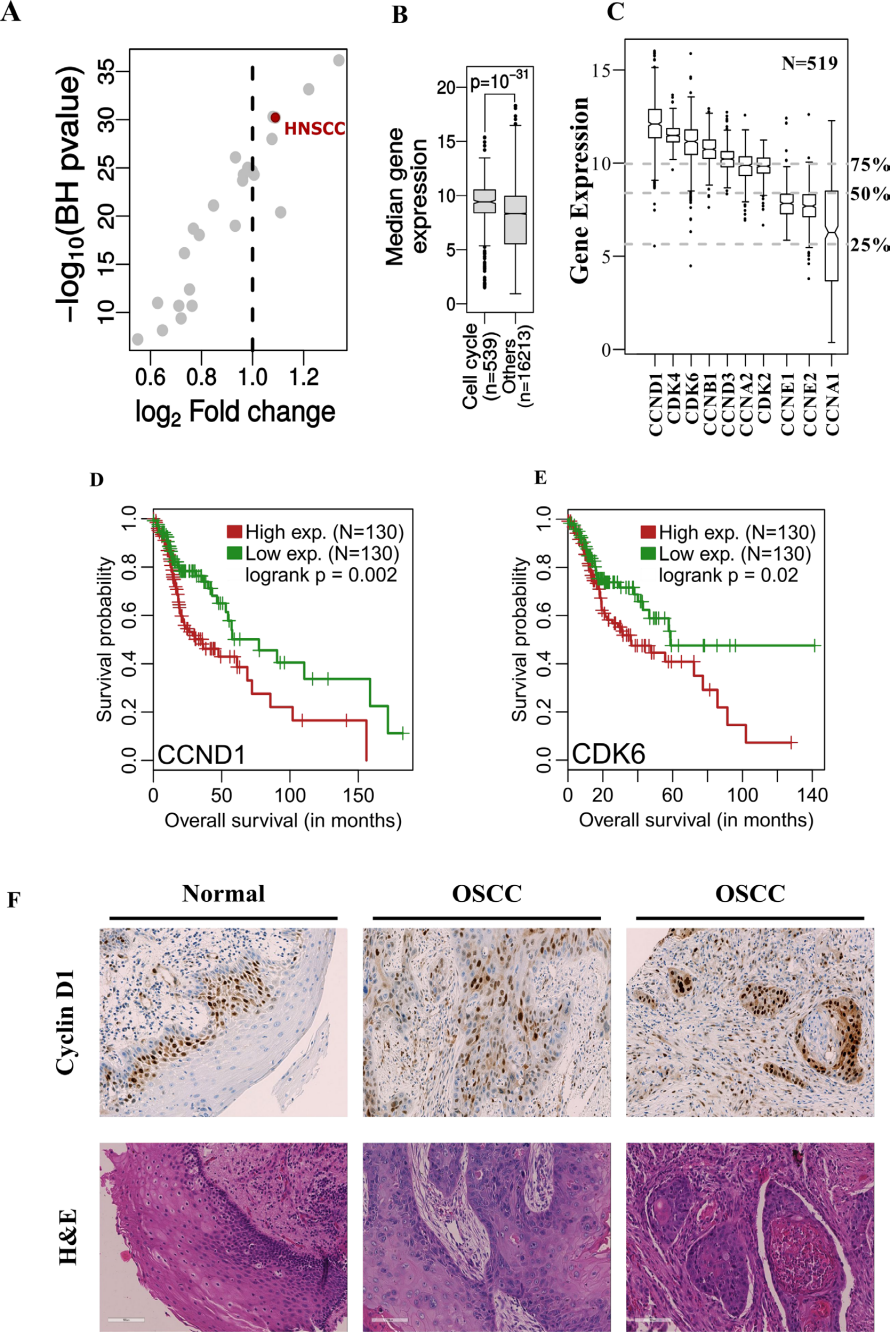
(**A**, **B**) The median expression of cell-cycle related genes in HNSC patient samples (*N* = 519) is significantly higher compared to other genes and this difference is among the highest across multiple tumor types (*N* = 24). (**C**) Gene expression of cyclins and CDK’s in HNSC patients. The grey dotted lines represent the 25%, 50% and 75% quantiles of median gene expression of all genes measured (*N* = 16752). (**D**, **E**) Kaplan-Meier’s survival curve showing patient survival related to over-expression of CCND1 and CDK6 when stratified by the top and bottom 25 percentile of respective gene expression. (**F**) IHC and H&E stains of HNSCC patient derived tumor and control samples. An increased intensity of CCND1 staining is seen in patient samples when compared to the control tissue samples.

In order to assess the extent of deregulation of cell cycle genes across different cancer types, a pan-cancer analysis of variation in average expression of cell cycle related genes across multiple cancer types (*N* = 24) was carried out. The median expression of cell cycle related genes (*N* = 539; using the gene ontology term GO: 0007049) was compared with the median expression of all other genes in each cancer type (Figure 1A, for details on the cancer types analyzed, see Supplementary Table 2). We observed that the median gene expression of cell cycle related genes was significantly increased over that of other genes. However, there was only a modest variation in the effect sizes observed (minimum and maximum log2 fold changes ranged from 0.55 to 1.34). Only 8 tumors were characterized with a unit fold change including head and neck squamous cell carcinoma (HNSCC), which ranked among the highest (*p* = 10^–31^, fold change = 1.09, Figure 1A and 1B). Permutation analysis was then carried out to verify the higher expression of cell cycle genes in HNSCC tumors and interestingly, the fold change distribution generated from the randomly labeled cell cycle genes was significantly lower than the real dataset (*p* = 10^–4^, Supplementary Figure 1A) suggesting that indeed there is higher expression of cell cycle genes in HNSCC tumors.

Intrigued by the finding that cell cycle genes were most deregulated in HNSCC among cancer types, the gene expression of CDKs and cyclins in HNSCC cancer patients was studied. Remarkably, cyclins and CDK’s generally exhibited high expression in HNSCC patient samples (*N* = 519), most of which showed a higher value than the median global gene expression (Figure 1C). Furthermore, we observed that the expression of cyclins and CDKs was significantly higher in tumors compared to their corresponding matched normal tissues in a smaller sub-cohort (*N* = 43) (Supplementary Figure 1B). In this respect, it is important to note that the average absolute expression for these genes in both normal and tumor samples is higher than the genomic average. Survival analysis was then carried out with all the genes listed in Figure 1C to decipher whether the higher expression of cyclins and CDK’s had any influence on patient survival. It was found that CCND1 and CDK6 affected patient survival. The overall survival probability between these groups was significantly different when stratified by the top and bottom 25 percentiles of respective gene expression levels (CCND1: *p* = 0.002 and CDK6: *p* = 0.02, Figure 1D and 1E). As CCND1 had a more significant effect on patient survival outcome, an IHC staining of 28 OSCC patient samples and matched controls was carried out to verify these findings. These stainings revealed increased expression of cyclin D1 in HNSCC patient tumor samples when compared with the control tissue (Figure 1F). In normal tissue, positivity of cyclin D1 was restricted to basal layers whereas in tumor samples positive nuclear staining was observed in all areas, with 80% of the cases showing >20–70% positive nuclear staining (Supplementary Figure 2).

### Erufosine causes inhibition of OSCC cells

**Figure 2:**
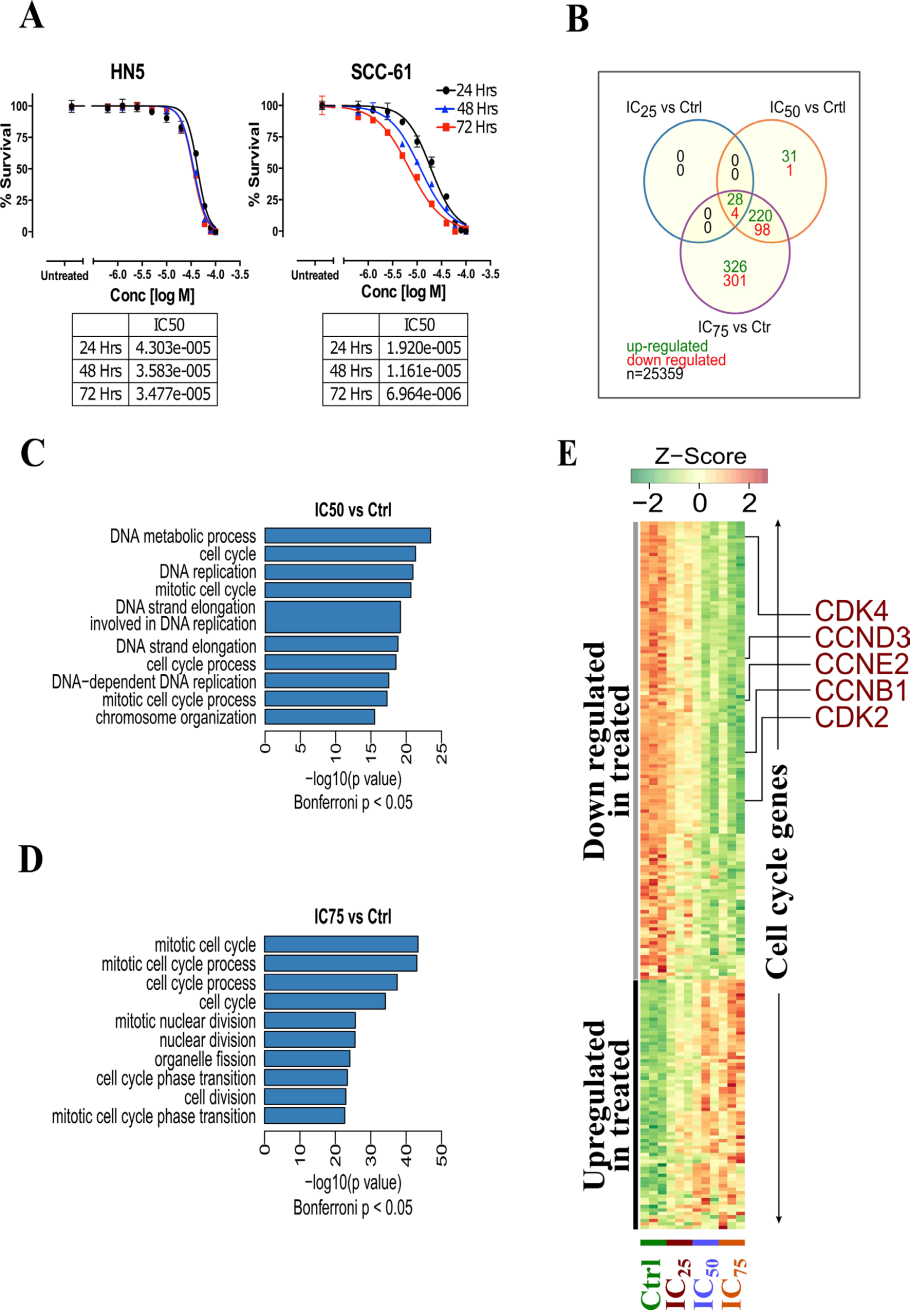
(**A**) Cytotoxicity of erufosine in two OSCC cell lines HN-5 and SCC-61 as determined by MTT assay at 24, 48 and 72 h. The IC50 values and its 95% confidence intervals are given below the graphs. (**B**) Transcriptomic analysis of erufosine treated and untreated HN5 cells at varying concentrations and the overlap among deregulated genes across various erufosine treatment conditions. (**C**) Highly enriched (top 10) GO terms found in significantly up-regulated genes identified in IC50 vs control and (**D**) IC75 vs control comparisons. (**E**) Heat map showing the expression dynamics of cell cycle related genes upon erufosine treatment at different concentrations.

In order to estimate the anti-proliferative activity of erufosine, a concentration-response study was performed in human HN-5 and SCC-61 OSCC cells. For both cell lines a concentration and time dependent inhibition of proliferation was seen in response to erufosine. The IC_50_ values ranged from 43–34 µM for HN-5 and from 19–7 µM for SCC-61 cells for time periods of 24–72 h (Figure 2A). SCC-61 cells were 2–5 fold more sensitive to erufosine than HN-5 cells. Based on the growth inhibition curves in response to erufosine exposure, the IC_25_, IC_50_ and IC_75_ values of HN-5 and SCC-61 cells were 27, 39, and 55 µM as well as 7.5, 15 and 30 µM, respectively, which were used for subsequent experiments.

### Cell cycle processes are downregulated by erufosine

To identify the mechanism of action of erufosine’s anti-neoplastic activity in OSCC cells, a gene expression profiling and enrichment analysis was performed. We investigated the global changes in gene expression (*N* = 25,359 genes) between erufosine treated HN-5 cells (at IC_25_, IC_50_ and IC_75_ concentrations) and untreated controls in triplicates (*N* = 12; 9 treated i.e. 3 for each concentration and 3 controls). The overlap among differentially expressed genes (at BH corrected *p* value < 0.05, and –1 > log2 fold change >1) is shown in Figure 2B. A total of 28 upregulated genes and 4 genes, which were downregulated, were observed among all concentrations (See Supplementary Table 3A–3C for complete list of deregulated genes). Furthermore, a gene enrichment analysis was performed to identify gene ontology terms being enriched with significantly down regulated genes. We observed that GO terms related to the cell cycle were significantly enriched in each treatment concentration (Bonferroni corrected *p*-value < 0.05, Figure 2C and 2D). The complete list of GO terms being enriched with down-regulated genes is given in Supplementary Table 4A and 4B. The heat map of all significantly altered cell cycle related genes is shown in Figure 2E. It illustrates the gradual decrease in expression of most cell cycle activating genes including CDK2, CDK4, CCNE2, CCND3 and CCNB1 upon increasing concentrations of erufosine treatment.

### Verification of cell cycle gene down regulation in OSCC cell lines

**Figure 3:**
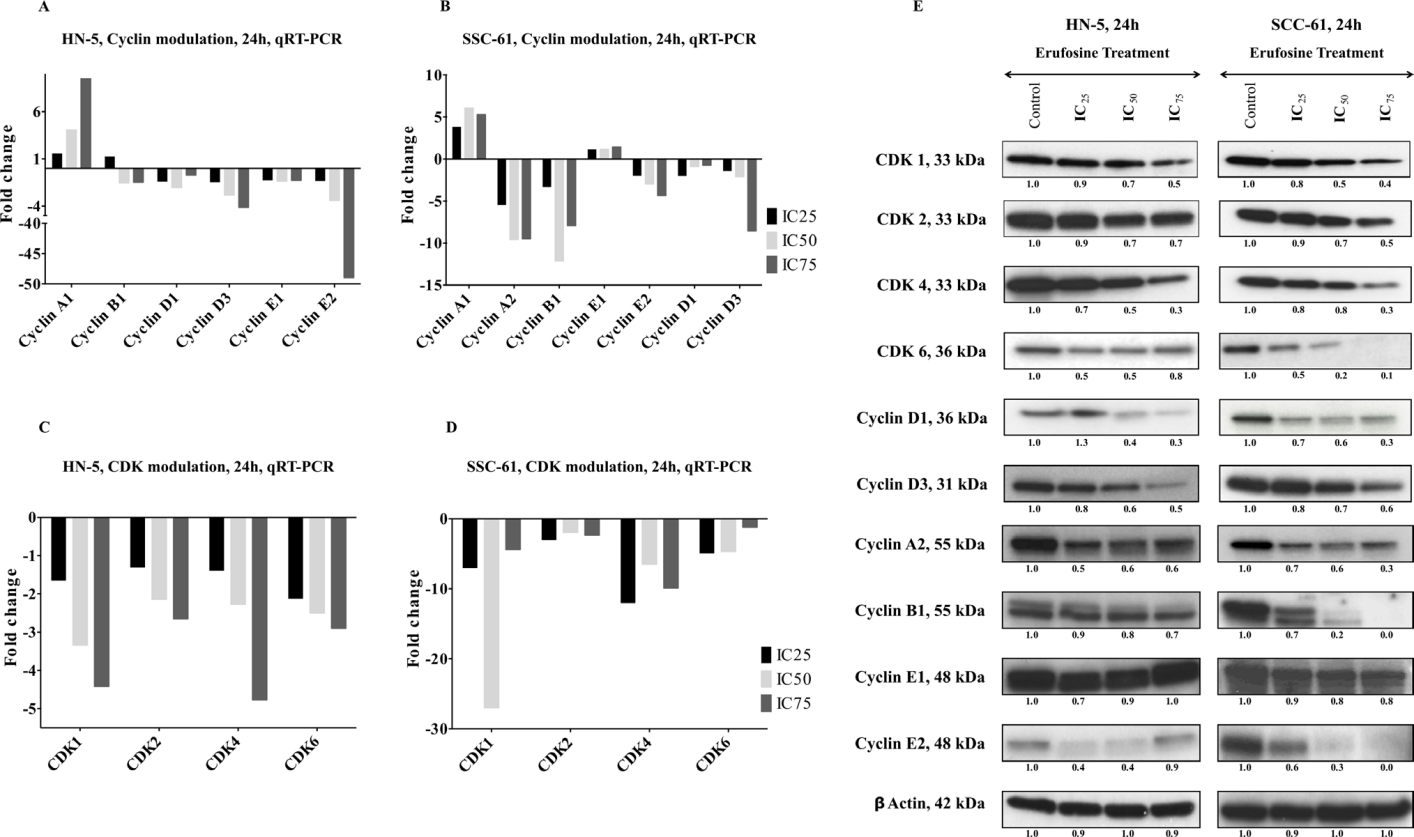
(**A**, **B**) qRT-PCR verification of cyclins in two OSCC cell lines, HN-5 and SCC-61. The expression of cyclin B1, cyclin D1, cyclin D3, cyclin E1 and E2 showed a dose dependent decrease in both cell lines post erufosine exposure. (**C**, **D**) qRT-PCR verification of CDks in HN-5 and SCC-61cell lines post erufosine exposure. A dose dependent decrease in all CDKs was observed Fold changes are depicted as averages of triplicate experiments. (**E**) Changes in protein levels determined by Western blotting. HN-5 and SCC-61 cells were exposed to erufosine concentrations for 24 h. A dose dependent decrease was observed in all the CDKs and the associated cyclins in both cell lines. Protein level changes were deduced by dividing the densitometry output for each band by that for the corresponding β-actin band.

In order to delineate the effect of erufosine on cell cycle progression in cells of OSCC cell lines, mRNA expression was determined by qRT-PCR of all cyclins and CDK’s in HN-5 and SCC-61 cells after treatment with respective IC_25_, IC_50_ and IC_75_ concentrations for 24 h. A dose dependent decrease was observed for all CDKs (CDK1, 2, 4 and 6) in both cell lines with CDK1 being most down regulated with 5 fold and 20 fold downregulation in HN-5 and SCC-61, respectively (Figure 3A and 3B). Additionally, decreased levels of all cyclins (CCNA1, A2, B1, E1, E2, D1, D2 and D3) were observed in both cell lines in response to erufosine exposure. A maximal downregulation of cyclin E2 by 50 fold was observed in HN-5 cells whereas Cyclin B1 showed 12 fold downregulation in SCC-61 cells when compared to the control cells (Figure 3C and 3D).

The down regulation of cyclins and CDKs upon exposure to erufosine at mRNA level was corroborated by immunoblotting the respective proteins. HN-5 and SCC-61 cells were exposed to IC_25_, IC_50_ and IC_75_ concentrations of erufosine for 24 h and lysed for protein blotting. In correlation with the mRNA findings, a dose dependent decrease was observed of CDK levels in both cell lines. Maximally reduced levels were seen for CDK4 in HN-5 (70%) and for CDK6 in SCC-61 (90%) cells, when compared to control cells. Next, when looking into the protein modulation of the respective cyclins, we found decreased cyclin D1, cyclin D3, cyclin A2, cyclin B1, cyclin E1 and cyclin E2 levels. Most pronounced decreases were observed at IC_75_ erufosine concentrations for cyclin D1 levels in HN-5 (70%) and up to complete inhibition of cyclin B1 and cyclin E2 in SCC-61 cells (Figure 3E).

### Erufosine causes G2/M block and reduced colony formation in OSCC cells

**Figure 4:**
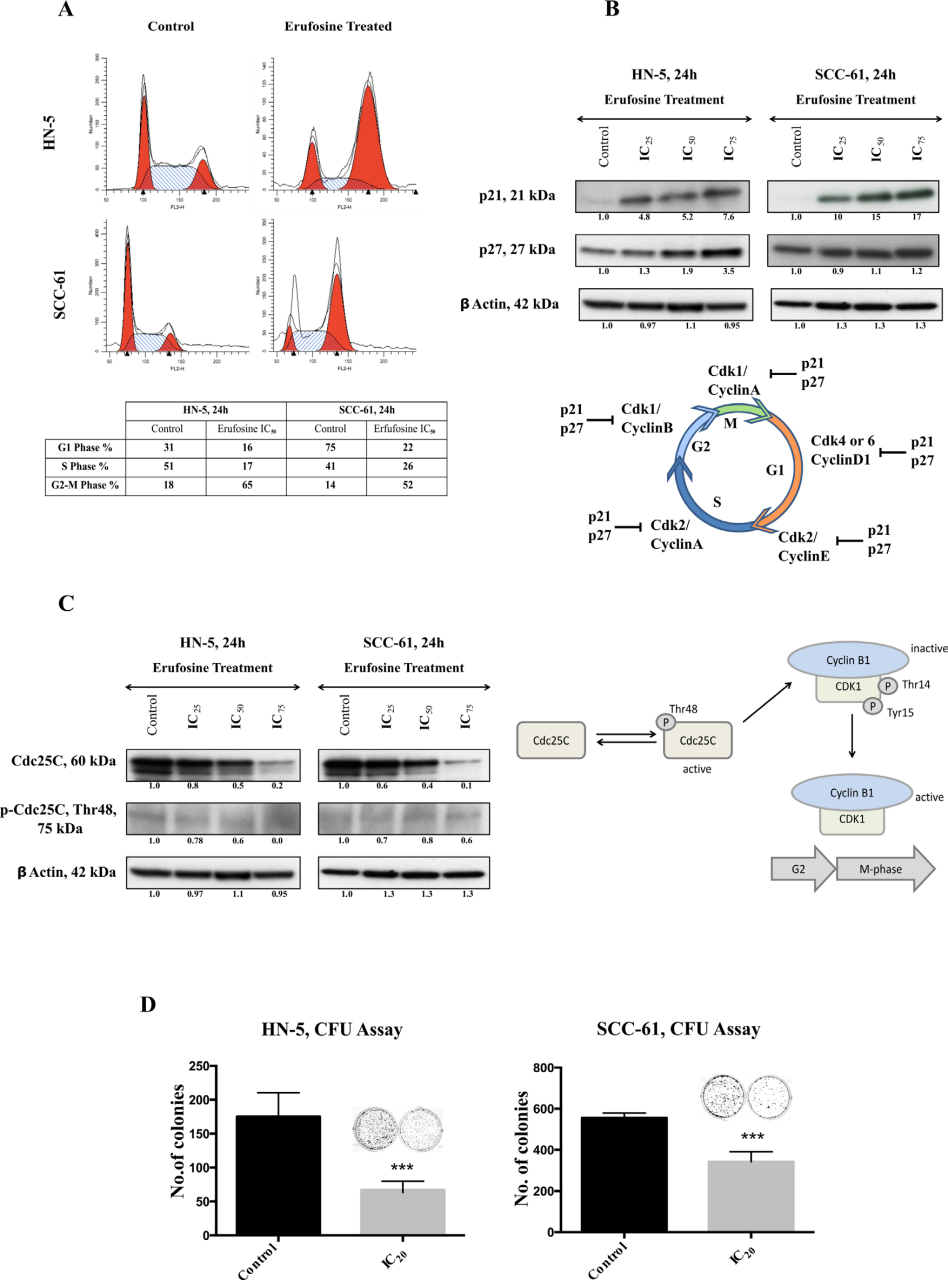
(**A**) Cell cycle analysis of HN-5 and SCC-61 post 24 h of erufosine treatment. Both cell lines were treated with IC50 concentration of the drug and analyzed by PI staining. Both cell lines show a G2/M block. Cell fractions (%) from different phases of the cell cycle are given in the table below the figure. Ten thousand cells (events) per sample were analyzed to study distribution of the cells. (**B**) The protein expression of p21 and p27 in response to erufosine exposure in HN-5 and SCC-61 cells. An increase in expression was seen in both p21 and p27 levels. (**C**) Protein expression of Cdc25C and p-Cdc25C Thr48 in response to erufosine exposure post 24 h in HN-5 and SCC-61 cells. A dose dependent decrease in protein levels was observed. Protein level changes were deduced by dividing the densitometry output for each band by that for the corresponding β-actin band. (**D**) Inhibition of colony formation by erufosine: Significant anti-clonogenic effects were observed in HN-5 and SCC-61 cells after exposure to erufosine at IC20 concentrations. Asterisks denote significant differences (*P* < 0.05) between the control and treated cells.

In order to correlate the downregulation of cyclins and CDK’s with effects on cell cycle distribution, the OSCC cells were stained with PI following exposure to erufosine. In both cell lines, a G2/M block was observed in response to erufosine. The share of G2/M phase cells in erufosine treated HN-5 cells (65%) was 3.8 fold higher than that in control cells (18%). Likewise, a G2/M arrest was seen in SCC-61 cells, with 52% cells stranded in the G2/M phase in erufosine treated cells compared to 14% in untreated cells, corresponding to a 3.7 fold increase over the control (Figure 4A). In conjecture with these findings, the levels of p21 and p27 showed a dramatic increase in response to erufosine in both cell lines (Figure 4B). As decreased complexes of cyclin D1/CDK4 or -6 would have favored a G1/S block, we checked for possible modulating factors. One factor was the dose-dependent decrease in total and phosphorylated Cdc25C levels (Figure 4C). Active Cdc25C will activate Cyclin B1/CDK1 complexes, which then will enable transition from G2 to M phase. Thus, lack of active Cdc25C probably contributed to the massive G2/M block observed.

Next, the potential to inhibit colony formation in OSCC cell lines was determined by using IC_20_ concentrations of erufosine. This treatment caused reduced colony formation as indicated by 60% inhibition in HN-5 cells and 40% inhibition in in SCC-61 cells, respectively (Figure 4D). These results show that in addition to inhibiting the expression of cyclins and CDK’s, erufosine also confers a strong functional block in cell cycle progression from G2 to M phases and inhibits the ability of cells to form colonies.

### Erufosine inhibits the CDKs and cyclins *in-vivo*

**Figure 5:**
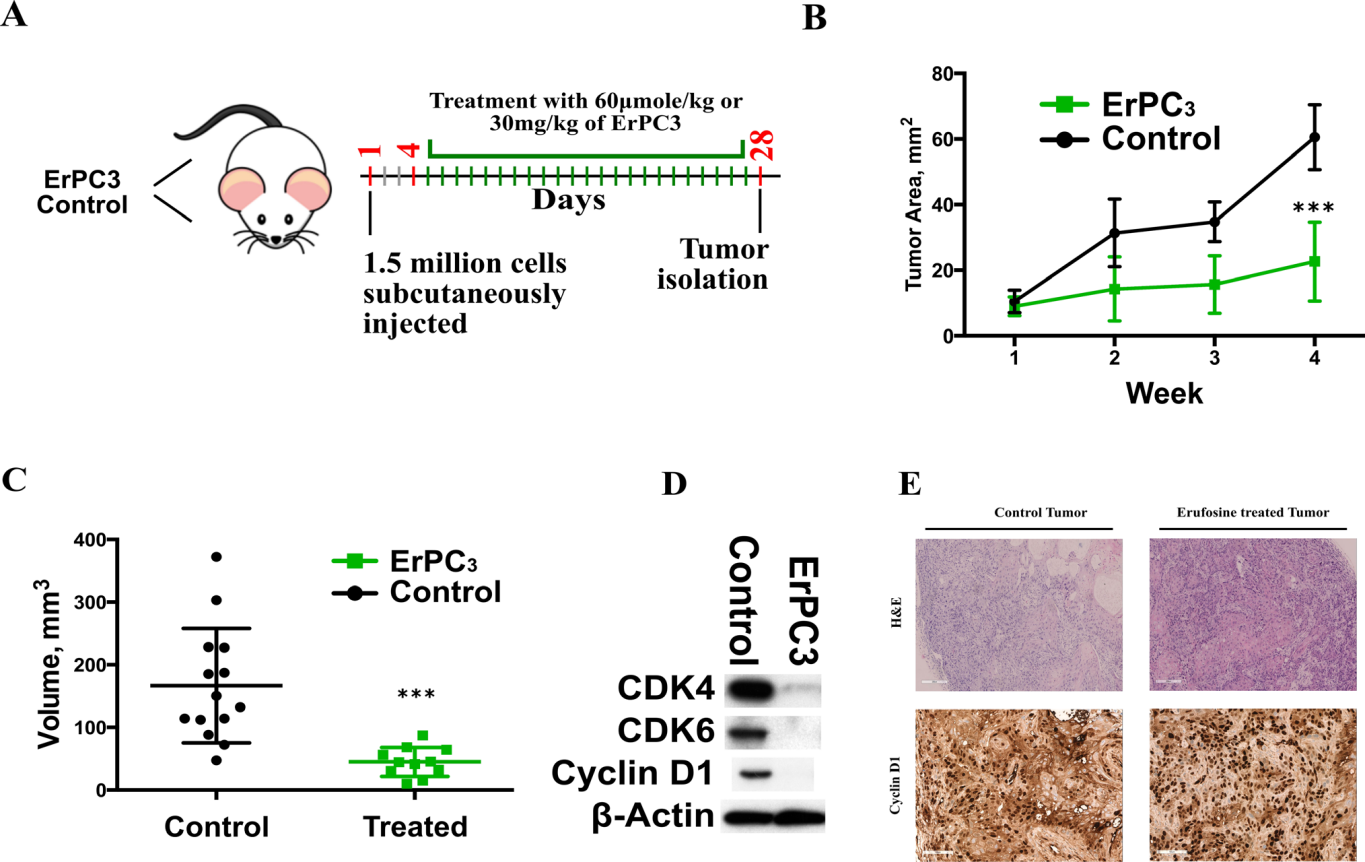
(**A**) Experimental design of the xenograft model used for assessing erufosine’s activity *in vivo*. Control and erufosine treated mice received twice weekly intraperitoneal injections of PBS or erufosine. (**B**) Change in the tumor area (mm^2^) between the control and erufosine treated groups during the course of experiment. A significant difference in favor of erufosine was observed in the tumor growth area between control and erufosine treated mice. (**C**) Mean tumor volume (mm^3^) of the control and erufosine treated groups at the end of the experiment. A significant reduction was seen in the tumor volume after erufosine treatment from 12 control and treated mice tumors. Asterisks denote significant differences (*P* < 0.05) between the control and treated cells. (**D**) Protein expression from excised control and treated lesions post erufosine treatment. A decrease in cyclin D1, CDK4 and CDK6 was observed in the excised treated samples when compared to the control lesions. (**E**) IHC (for Cyclin D1) and H&E stainings of erufosine treated and untreated tumor samples derived from the xenograft model. A reduced level of Cyclin D1 staining intensity is seen in the erufosine treated samples when compared to the untreated control groups.

Having demonstrated that erufosine causes down regulation of cell cycle processes *in-vitro* by inhibiting cyclins and CDKs, and causing a G2/M transition block, the anti-neoplastic efficacy of erufosine was tested in a xenograft mouse model. After 4 days of tumor establishment, the animals received either PBS or erufosine (60 µmol/kg) for a total period of 28 days (Figure 5A). The erufosine treatment was well tolerated in animals as indicated by absence of weight loss or any adverse changes seen during autopsy examination (data not shown). The treatment of xenograft bearing mice with erufosine caused a significantly reduced increase (22 mm^2^, ± 6) in the tumor area when compared to the control animals (60.6 mm^2^ ± 4; Figure 5B). In line with this, the tumor volume of all treated samples (18mm^3^, ± 6) was significantly (*p* < 0.01) lower than the volume of the control group (132mm^3^, ± 33) (Figure 5C). At the end of the experiments the tumors from control and treated animals were excised and either fixed in 4% formalin for IHC or in liquid nitrogen for protein extraction. Immune-blotting for cyclin D1, CDK4 and CDK6 was performed for lesions from control and treated animals. A decrease by 90% in the cyclin D1, CDK4 and CDK6 levels was observed in the erufosine treated tumor samples (Figure 5D). Our data revealed that erufosine not only decreases cyclins and CDKs *in-vitro* but also *in-vivo.* These data were confirmed for CCND1 by IHC staining, which was carried out in control and erufosine treated lesions. The lesions from erufosine-treated mice revealed reduced CCND1 staining intensity compared to control tumors, which corresponds to the decreased CCND1 protein levels. In line with this, H&E stains showed that adenoid structures as well as necrotic areas were reduced in erufosine treated compared to untreated tumors (Figure 5E).

## DISCUSSION

Deregulation of cell cycle has been implicated in most human cancers and leads to cell proliferation, chromosome instability and loss of genomic integrity [4, 29, 30]. Cancer cells show deregulated cell cycle progression with either overexpression of positive regulators and/or inhibition of negative regulators, which renders them with unrestrained replication potential [31]. In a recent study it was shown that aberration in cell cycle genes was seen in 39% of cancers analyzed from patient samples [32]. In the present study we analyzed the extent of deregulation of cell cycle processes, annotated with GO terms, across various cancers in the TCGA database by comparing the median gene expression of cell cycle related genes with all other genes in each cancer type. Our findings revealed that Head and Neck cancer ranks amongst the top four cancers with deregulated cell cycle processes. These results were further pivoted by a recent finding, where the cell cycle processes were over-represented in 498 HNSCC samples when compared to the controls [33]. It is known that components of the CDK4–cyclin and CDK6–cyclin complexes are frequently altered in cancer. We observed from HNSCC patient data (*N* = 519), that most of the cyclins and CDKs have a higher median gene expression when compared to the median global gene expression. This entails that most of the CDKs and cyclins tend to be altered in HNSCC, which may be caused by either overexpression of cyclins (mainly D1 and E1) and/or CDKs (mainly CDK4 and CDK6), as well as loss of CKI (mainly INK4A, INK4B and KIP1) and RB expression [34].

We then checked for potential changes in patient survival related to increased expression of CCND1, CDK4 and CDK6 in HNSCC patients. We found that increased expression of CCND1 and CDK6 significantly correlated to reduced probability of patient survival. These findings go on to suggest that CCND1 and CDK6 play a vital role in HNSCC, are involved in tumor progression and can be used as a clinical biomarkers for advanced stages of HNSCC. CCND1 has an oncogene status and substantial evidence exists for its involvement in amplification and overexpression in breast, lung, and oral squamous cell cancers as well as melanomas [35]. This is in line with our IHC findings from patient samples. We found increased expression of CCND1 in 28 HNSCC patient derived samples when compared to the control samples. In contrast, CDK4 amplification in head and neck cancers is not prominent with 2.1% of 900 cases showing amplification and 2.4% showing mutation of the gene (Supplementary Figure 3). Our IHC data also revealed no detectable expression of CDK4 in patient samples. Nevertheless, available reports suggest that CDK4 expression is crucial for the development of mammary [36, 37] and small-cell lung carcinomas [38] in mice. Enhanced levels of CDK6 have been detected in patients with squamous-cell esophageal carcinoma [39] and head and neck squamous cell carcinoma [40] with high nuclear CDK6 expression tending to have advanced tumor status.

It is hence no surprise that in recent years efforts have been made to develop new agents, which target the deregulated cell cycle and are therefore considered as an attractive strategy for cancer therapy. CDKs are actively being targeted due to their central role in the control of cell-cycle progression. The complexity of CDK regulation offers a number of possible routes to their therapeutic inhibition [34]. The most promising strategies involve designing of inhibitors that block cyclinD-CDK4/6 complex activities [41].

**Figure 6:**
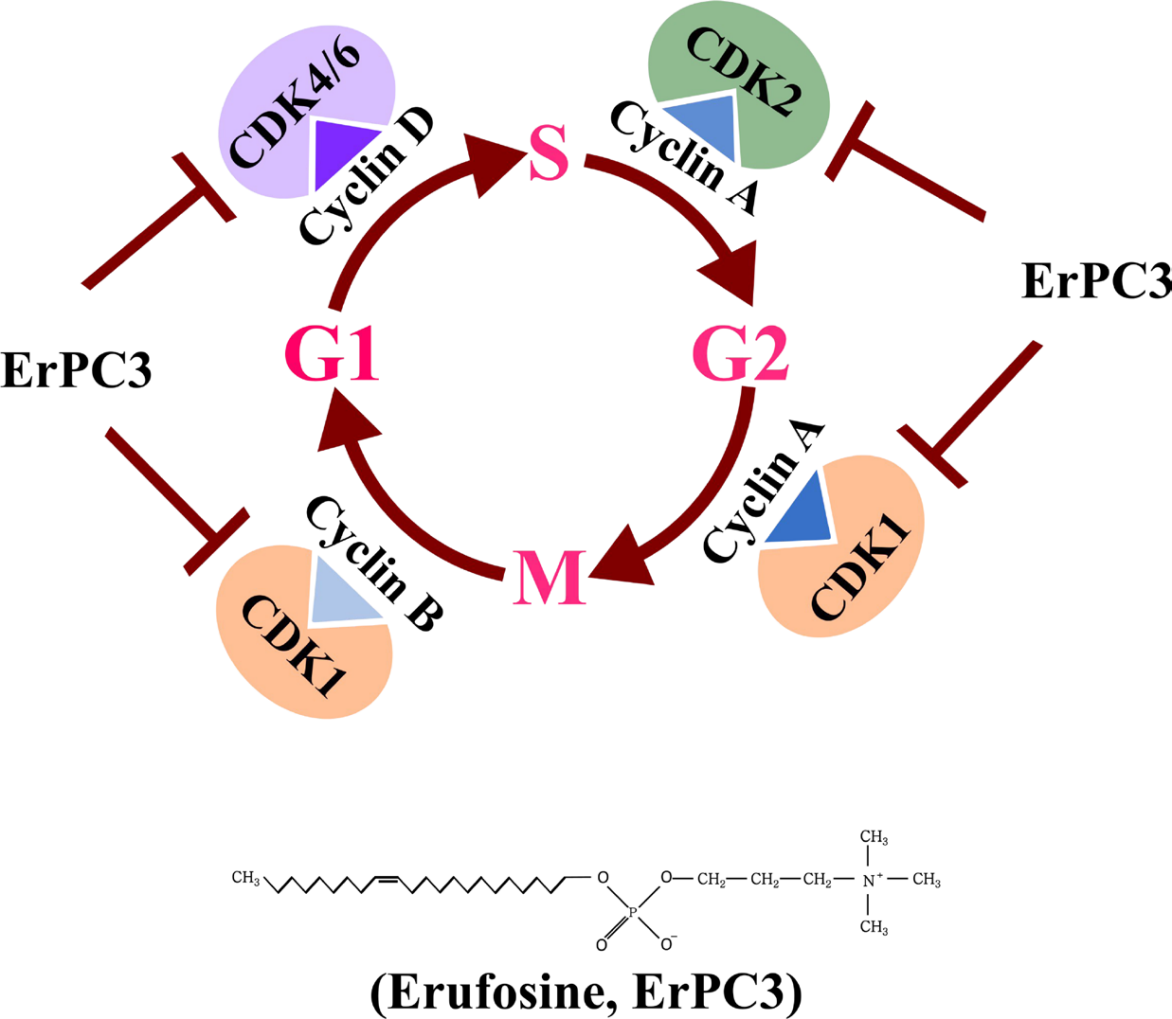
Graphical representation of Pan CDK inhibition activity by erufosine in OSCC cell lines and *in-vivo*

The alkylphosphocholine erufosine is a known inhibitor of the Akt/mTOR pathway and induces apoptosis in OSCC cells [28]. As expected, erufosine caused cytotoxicity in HN-5 and SCC-61 OSCC cells and for better understanding of its mode of action we investigated global changes in gene expression in response to erufosine exposure in HN-5 cells in a dose and time dependent manner. Interestingly, after gene enrichment analysis, we found that the downregulated genes included those for cell cycle processes, which were negatively enriched in a dose dependent manner. We observed a significant down regulation of CDK2, CDK4, CCNE2, CCND3 and CCNB1 from our microarray findings, which caused us to hypothesize that erufosine can inhibit the cell cycle progression in OSCC. Confirmatory investigations with qRT-PCR and Western blot showed that erufosine not only inhibited the expression of these genes but also caused inhibition of almost all other cyclins and CDK’s. The strength of this effect nevertheless varied in the two cell lines at protein level, as indicated by inhibition of the cyclin dependent kinases (CDK4>>CDK1>CDK2>CDK6) in HN-5 cells as compared to SCC-61 cells (CDK6>CDK4>CDK1>CDK2). These results show that erufosine acts like a pan-CDK inhibitor (Figure 6). We also observed that erufosine caused 70% inhibition of cyclin D1 at protein level in both cell lines. Interestingly, the mRNA expression of CCND1 as detected by qRT-PCR was not remarkably reduced, which is in a good concurrence with the microarray data. The reason for this apparent discrepancy between the mRNA and protein levels might be caused by cyclin D1 being a downstream target of the Akt pathway [42], which is in turn inhibited by erufosine [28].

When comparing the uniform downregulation of cell cycle related genes in response to erufosine with parallel effects after clinically used antineoplastic drugs, distinct differences are apparent. For drugs, which are not cell cycle specific, mainly changes linked to DNA damage can be observed, which in turn will cause changes in cell cycle distribution. However, these cell cycle changes are not necessarily caused by alterations in the expression of cell cycle related genes, as has been described recently for cisplatin [43].

For interpreting the observed cell cycle block at G2/M phases in response to erufosine, the following considerations may be valid. 1.) From Western blot analyses there was a concentration dependent reduction in levels of cyclinD1 and CDK4 in both cell lines. This combined reduction of the CylinD1/CDK4 complex explains the concomitant reduction of pRB levels, which is required for transition through G1 phase. Presumably because of the reduced pRB/RB levels (Supplementary Figure 4), the cells transited unrestrained from G1/S phases. 2.) Cyclin/CDK complexes formed from cyclin A/CDK4/6 and cyclin E/CDK2, which are also required during the transition from G1 to S phases, where likewise considerably reduced. This reduction coincided with increased levels of p21 and p27, which presumably further reduced the number of cells in G1 and S phases. The significant increase in p21 levels, which was accompanied by reduced cyclin B1 and CDK1 levels, may in turn have been responsible for the massive blockage of cells in G2/M phase. 3.) The massive block in G2/M phases was further intensified by decreased levels of Cdc25C, which was reduced in both, the phosphorylated and total protein levels. The reduced p-Cdc25C levels were insufficient to activate cyclin B1/ CDK1 complex and hence resulted in the observed G2/M block.

These changes presumably caused the OSCC cells to undergo apoptosis. The consequence of this massive cell cycle block manifested in all cell based assays including those for proliferation and colony formation, as well as in the anti-neoplastic activity *in-vivo*. Interestingly, the range of erufosine concentrations used in the cell based assays corresponded well to that, which was determined in human plasma (0.93–29.8 μM) obtained from clinical pharmacokinetic studies [44] and hence reinforces the use of erufosine in in a clinical setting

In nude mice, we showed that erufosine at 30 mg/kg dosage is able to inhibit tumor growth of HNSCC xenografts over a period of 4 weeks without causing toxic effects. This is the first study, which shows that erufosine inhibits HNSCC tumor growth *in-vivo*. In treated tumor tissues we also observed decreased protein levels of cyclin D1, CDK4 and CDK6 when compared to control tumors and the IHC staining of cyclin D1 showed reduced intensity in erufosine treated as compared to untreated tumors. This data correlates well with our *in-vitro* findings of decreased cyclin and CDKs levels. In addition, the correlation between our growth inhibition in xenografts with the clonogenic assay results from this study can be interpreted in the light of findings, which established a predictive role of clonogenic assays for the response of a given drug in tumor xenografts [45]. On the other hand, the response in tumor xenografts is known to have predictive value for the clinical outcome [46].

The findings of this study show a high correlation between erufosine exposure and cell cycle related genes, however, they do not provide a formal proof for this new aspect of erufosine’s mechanism of action. Erufosine, similar to ether lipids, inserts into the lipid bilayer of the plasma membrane. Sequentially it causes disturbance of the cell membrane leading to perturbation of the cellular signaling pathways, lipid and micro-domain turnover in order to induce the anti-neoplastic effects [47–49]. Although the decrease of certain cyclins and CDKs can be attributed to downregulation of certain signaling pathways (the Akt pathway controls the expression of cyclin D1), this does not fully explain the complete shutdown of all the cyclins and CDKs. It has been previously shown, that inhibition of the cell cycle regulator Rb via shRNA impeded the anti-neoplastic activity of erufosine [50]. Thus, this study gives a partial causal effect towards the role of erufosine on cyclins and CDKs and their responsibility in mediating the antineoplastic activity of erufosine through inhibition of the cell cycle arm.

However, for approving the primary and causal effects for e.g. inhibition of colony formation and cell viability, future studies will have to address the following challenges: Complete knockdown of cell cycle related genes, which could be seen as a tool to prove the importance of erufosine’s activity as pan-cyclin/CdK inhibitor, will be hard to achieve, as they are probably not compatible with cell survival. Only cells with specific mutations may tolerate such alterations, which in turn will not help in elucidating erufosine’s mechanism of action. Instead, a conditional knockdown strategy might help addressing this aspect, because a controlled decrease in target proteins could provide for both, reduced target gene levels and nevertheless maintained cell survival. In summary, for its low myelotoxic activity and inhibition of cell cycle processes both *in-vitro* and *in-vivo*, erufosine can be considered as a candidate for HNSCC treatment either alone or in combination with cisplatin, which is still the first line of therapy for HNSCC treatment.

## MATERIALS AND METHODS

### Pan cancer analysis of cell cycle related genes

Transcriptomics data and the corresponding patient overall survival information for 24 different cancer types studied by The Cancer Genome Atlas (TCGA) were obtained from UCSC cancer genomics browser (https://genome-cancer.ucsc.edu; TCGA Pan-Cancer data - version 2015-02-15). The gene ontology term “cell cycle” (GO: 0007049) was used to identify cell cycle related genes (data downloaded from http://amigo.geneontology.org/amigo/term/GO:0007049 on 15th April, 16). The gene expression levels of these were analyzed across multiple cancer types using standard functions in the R statistical programming environment (https://cran.r-project.org/). The survival package (http://CRAN.R-project.org/package=survival) was used for generating Kaplan Meier survival curves and the statistical significance of patient survival differences between stratified gene expression levels was calculated using the log rank test.

### Immunohistochemistry

28 paraffin-embedded blocks of oral squamous carcinoma tissues were obtained from Services Institute of Medical Sciences and King Edward Medical University, Lahore and the normal control stainings was provided by the tissue bank of the National Center for Tumor Diseases (Heidelberg, Germany). The informed consent was obtained from all patients. For immune-histochemical analyses, 4 µm sections were obtained. After heat-antigen retrieval in sodium citrate buffer, staining’s were performed using antibodies against cyclinD1 (Roche), using an automated immunostainer (Ventana BenchMark XT) and the UltraView Universal DAB detection kit. In addition, the excised tumors from control and treated animals were collected and processed for histology at the end of the study in order to perform H&E stains and cyclin D1 stains.

### Cell culture and reagents

Human oral squamous cell carcinoma cell lines, HN-5 and SCC-61, were obtained as a kind gift from Prof. Myers’ lab, MD Anderson Cancer Center, USA. HN-5 cells were cultured in DMEM:F-12 medium (Lonza, Germany) supplemented with 2 mM L-glutamine and SCC-61 cells were maintained in Dulbecco’s modified Eagle’s medium (DMEM) (Invitrogen, USA) supplemented with 20% fetal bovine serum (FBS), 2 mM L-glutamine, sodium pyruvate, nonessential amino acids and vitamin solution (Life Technologies, NY). Cell lines were maintained in a 37°C, 5% CO_2_ humidified incubator. The cell lines had been authenticated by MD Anderson center using short tandem repeat analysis and were routinely tested for mycoplasma contamination.

Erufosine (erucylphospho-N,N,N-trimethylpro-panolamine, ErPC3) was kindly provided by Prof. H. Eibl, Max Planck-Institute of Biophysical Chemistry, Goettingen, Germany. It was dissolved in saline at a concentration of 20 mM and stored at 4°C.

### MTT assay

The cell viability of OSCC cells in response to erufosine was determined by MTT [3-(4,5-dimethylthiazol-2-yl)-2,5-diphenyltetrazolium bromide] dye reduction assay as described previously [51].

### Microarray analysis and Gene enrichment

To analyze the gene modulation taking place in response to erufosine in HN-5 cells, a gene expression profiling assay was performed. The mRNA was collected to generate a time and concentration dependent profile of gene modulation. Briefly, HN-5 cells were treated with concentrations corresponding to IC_25_, IC_50_ and IC_75_ (27, 39 and 55 μM) of erufosine for 16, 24 and 48 h. mRNA was then extracted using the RNeasy Mini kit (Qiagen, Germany) following the manufacturer’s protocol and the extracted mRNA was subjected to microarray analysis by Illumina Chip array.

Background correction, variance stabilization and robust spline normalization of the expression data was performed using the lumi package from Bioconductor [52]. The normalized data was further analyzed for differential gene expression between erufosine treated and untreated cells using the limma package in R [53]. The heatmap for differentially expressed cell cycle genes was generated using the heatmap.2 function (gplots package) in R (www.r-project.org). Gene set enrichment analysis was performed separately for significantly up and down regulated genes (BH corrected *p* value < 0.05 and –1 > fold change > +1) using the GOstats package available in Bioconductor [54]. The raw data for the gene expression analysis has been submitted to the Gene Expression Omnibus database (GEO ID: GSE96599).

### cDNA synthesis and quantitative real time PCR (q-RT-PCR) analysis

Total RNA was isolated from both cell lines post 24 h of treatment as described before. Equal amounts of RNA (1 μg) were reverse transcribed using OligodT primers and Mu-MLV reverse transcriptase (Thermo Scientific) to obtain cDNA. qRT-PCR was carried out to verify the microarray findings, using 2× LC480 master mix along with an appropriate probe from the Universal probe library (Roche). Experiments were performed in triplicates and the expression level of glyceraldehyde 3-phosphate dehydrogenase (GAPDH) was used for normalization. The fold-changes were calculated by the 2^–∆∆CT^ method. (See Supplementary Table 1 for primer sequences).

### Immuno-blotting

Protein isolation and immune blotting was performed as previously described [51]. The following primary antibodies were used CDK1 (Abcam), CDK2, CDK4, CDK6, cyclin A, cyclin B1, cyclin D1, cyclin D3, cyclin E1, cyclin E2, p21, p27, Cdc25C, pCdc25C Thr48 (Cell Signaling Technologies), and Rb, pRb, β-actin (Santa Cruz). HRP-conjugated anti-mouse, anti-rabbit (Cell Signaling Technologies) or anti-goat (Santa Cruz) secondary antibodies were used and the immune blots were developed by ECL solution (Perkin Elmer). Densitometry was performed using the ImageJ software.

### Cell cycle analysis

Propidium iodide (PI) fluorescent staining and flow cytometry was carried out to study the cell cycle progression in the two cell lines after 24 h of erufosine exposure as described previously [51].

### Clonogenic assay

The colony forming ability of HN-5 and SCC-61 was determined after erufosine exposure. Briefly, after 24 h exposure to IC_20_ concentration of erufosine, 500 cells were seeded in 2ml of medium in a 6 well plate. Cells were incubated under normal cell culture conditions (37°C, 5% CO_2_ in humidified air) and colonies were counted microscopically after 8 days. Clusters of cells >30 cells were considered as colonies. Further, colonies were fixed with 1 ml of fixative (methanol/acetic acid, 3:1), washed in 1× PBS and stained with crystal violet dye (0.5% w/v in 1× PBS). All experiments were performed in triplicate and data was represented as percentage of colony forming units.

### Animal experiments

Female nude mice were obtained (Charles River, Germany) at an age of 4 to 6 weeks and kept under specific pathogen free (SPF) conditions in Macrolon-III-cages of a ventilated rack. Constant room temperature (22 ± 1°C), air humidity (50 ± 10%) and dark-light-rhythm (12 h) were maintained throughout. The animals had free access to autoclaved water and standard laboratory diet. After an adaptation period of a week, experiments were started. All animal experiments were performed in accordance with institutional and governmental regulations, and were approved by the Regierungspräsidium (Karlsruhe, Germany).

HN-5 cells (1.5 × 10^6^/100 µl) were injected subcutaneously into the flanks of the animals, with 6 animals being used in each experimental group. After 4–5 days of tumor development, mice received either PBS or erufosine as treatment, administered intraperitoneally twice a week (60 µmole/kg or 30 mg/kg). Animal weight and tumor dimensions were measured weekly and tumor volume was calculated as a × b^2^ × 0.5, where a > b. After 4 weeks, animals were sacrificed and tumors were isolated for western blotting and IHC staining’s. The experiments were performed in duplicates.

### Statistical analysis

In addition to statistics used in gene expression, gene enrichment analysis and Kaplan–Meier plots were used. Student’s *t*-test was used to determine statistical significance of differences between groups using the GraphPad Prism for other experiments. All the data was expressed as mean ± SD, while *P* values < 0.05 were considered as statistically significant.

## SUPPLEMENTARY MATERIALS FIGURES AND TABLES










