# Supplementary material for: Upregulation of cell cycle genes in head and neck cancer patients may be antagonized by erufosine’s down regulation of cell cycle processes in OSCC cells
Source: Oncotarget. 2017 Dec 20;9(5):5797–810. doi: 10.18632/oncotarget.23537 (PMC5814175; doi:10.18632/oncotarget.23537)
Supplement: Supplementary file 1 [file oncotarget-09-5797-s001.pdf]

Upregulation of cell cycle genes in head and neck cancer patients may be antagonized by erufosine's down regulation of cell cycle processes in OSCC cells

SUPPLEMENTARY MATERIALS

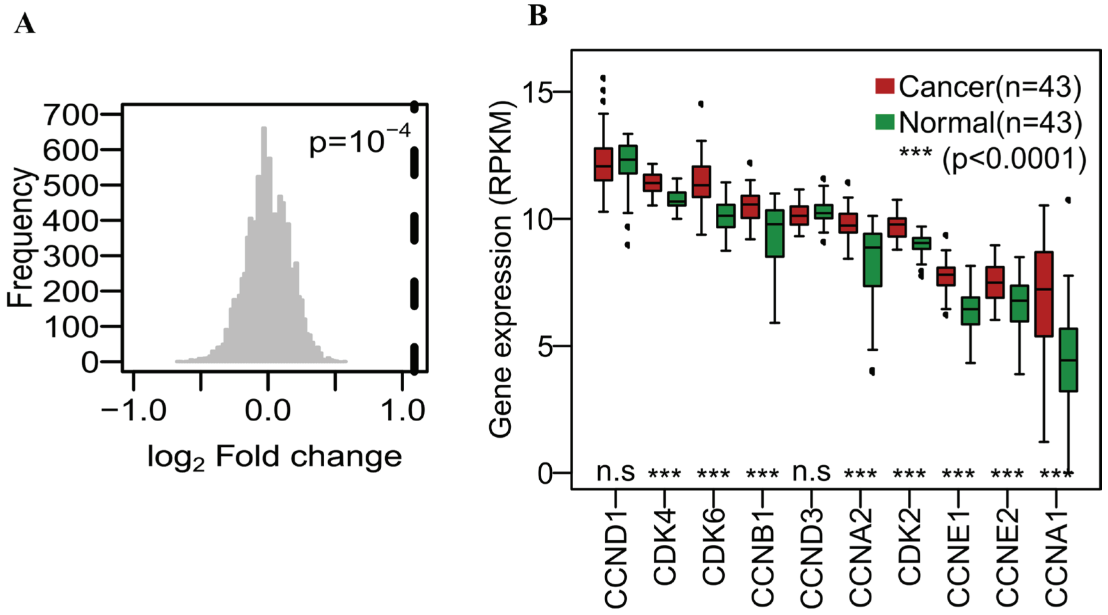

Supplementary Figure 1: (**A**) Increased expression of cell-cycle genes over median expression of all other genes is shown in HNSCC data from TCGA using permutation analysis. The histogram represents the distribution of fold differences (N = 10,000) between median gene expression of randomly assigned cell cycle genes and the remaining genes. The dotted line indicates the actual fold difference in expression observed between median gene expression of correctly assigned cell cycle genes and the rest. (**B**) The expression of cyclins and CDKs in HNSCC tumors and corresponding matched normal control samples.

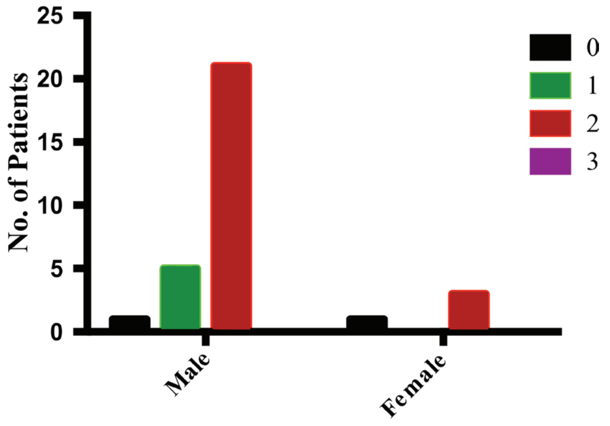

Supplementary Figure 2: Distribution of cyclin D1 nuclear staining intensity in 28 OSCC patients. The intensity of staining is categorized as 0 = negative staining, 1 = cells with up to 20% positive nuclear staining, 2 = cells with 20–70% % positive nuclear staining and, 3 = cells with minimum of 70% positive nuclear staining. More than 82% of the patients were found to belong to category 2.

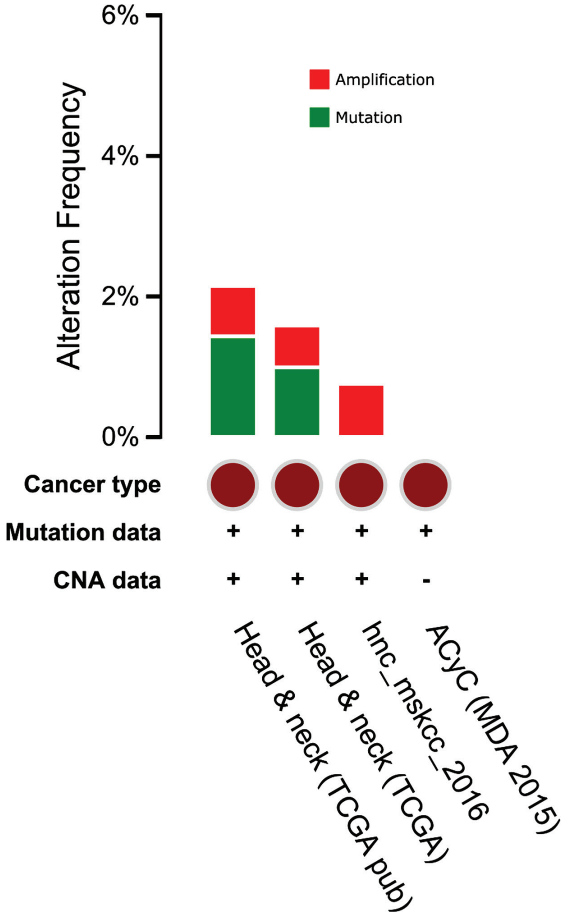

Supplementary Figure 3: Deregulation of CDK4 in head and neck cancer patients as revealed from samples available from cBioPortal.

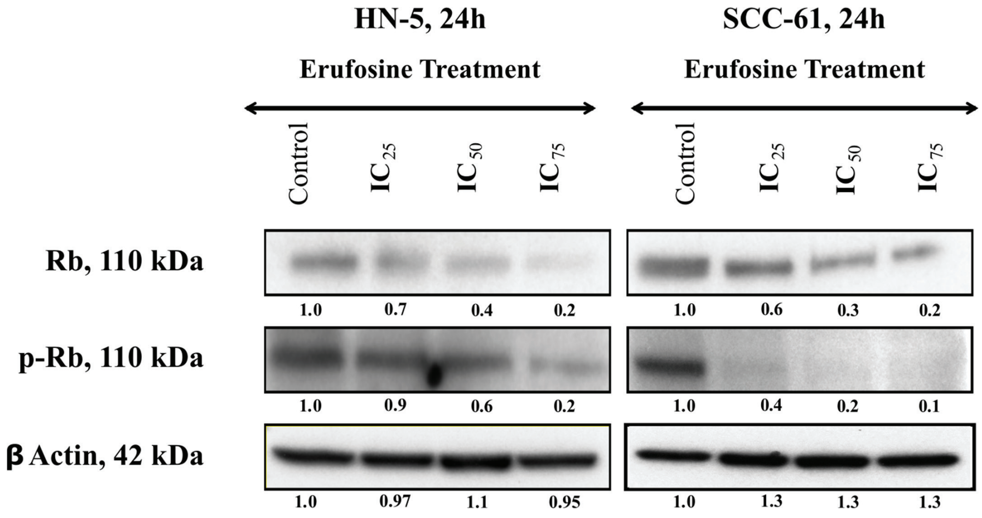

Supplementary Figure 4: Changes in the protein levels of Rb and p-Rb in two OSCC cell lines in response to erufosine exposure. A decrease in the levels of both Rb forms was seen in both cell lines in response to erufosine in a dose dependent manner.

Supplementary Table 1: Primer sequences for Cyclins and Cdks

Gene	Forward Primer	Reverse Primer	Probe Number[a]
CDK1	tggatctgaagaaatacttggattcta	tctggagatctgtaccagagtgtt	79
CDK2	tttggagtccctgttcgtactt	aaagatccggaagagctggt	80
CDK4	gtgcagtcggtggtacctg	ttcgcttgtgtgggttaaaa	25
CDK6	gaactaggcaaagacctacttctga	ggtgggaatccaggttttct	85
CCND1	gtgccggccttcctagtt	ggatttagggggtgaggtg	21
CCNA1	tagacaccggcacactcaag	ctaccagcataggggaaactgt	4, 18
CCNA2	ggtactgaagtccgggaacc	gaagatccttaaggggtgcaa\	84
CCNE1	cctcggattattgcaccatc	catgattttccagacttcctctc	61, 27
CCNE2	ggaagacagacataatatccagacac	cccccttttctgaaggtgtt	65, 67, 69
CCNB1	acatggtgcactttcctcct	aggtaatgttgtagagttggtgtcc	18
CCND3	gggatcactggcactgaag	cctgaggctctccctgagt	68

[a]Probe number recommended from Universal Probe Library, Roche.

Supplementary Table 2: Median expression of cell cycle genes verus all other genes across 24 cancer types

Cancer subtype	Cell cycle genes vs others genes fold change	Cell cycle genes vs others genes *p* value	Cell cycle genes vs others genes BH corrected	Sample size
testicular germ cell tumor	1.338	3e-38	7e-37	150
cervical & endocervical cancer	1.22	6e-35	7e-34	303
acute myeloid leukemia	1.11	2e-21	4e-21	173
head & neck squamous cell carcinoma	1.089	1e-31	6e-31	519
lung squamous cell carcinoma	1.08	6e-32	5e-31	502
thymoma	1.076	2e-29	1e-28	119
bladder urothelial carcinoma	1.006	2e-25	5e-25	407
rectum adenocarcinoma	0.999	6e-26	2e-25	94
colon adenocarcinoma	0.981	3e-26	1e-25	286
sarcoma	0.964	2e-25	5e-25	258
uterine corpus endometrioid carcinoma	0.961	1e-24	2e-24	174
ovarian serous cystadenocarcinoma	0.933	2e-27	8e-27	262
skin cutaneous melanoma	0.932	8e-20	1e-19	103
breast invasive carcinoma	0.847	4e-22	8e-22	1090
lung adenocarcinoma	0.791	6e-19	9e-19	508
glioblastoma multiforme	0.768	1e-19	2e-19	154
prostate adenocarcinoma	0.763	2e-11	2e-11	497
brain lower grade glioma	0.753	3e-13	4e-13	516
pancreatic adenocarcinoma	0.733	5e-17	7e-17	178
liver hepatocellular carcinoma	0.72	4e-10	4e-10	371
thyroid carcinoma	0.711	2e-11	2e-11	505
kidney papillary cell carcinoma	0.647	7e-09	7e-09	290
kidney clear cell carcinoma	0.628	1e-11	1e-11	529
pheochromocytoma & paraganglioma	0.55	6e-08	6e-08	179

Supplementary Table 3A: List of differentially regulated genes in HN-5 cells (IC25 vs control)

Symbol	Definition	Log FC	Avg. Expression	*t*-statistics	*p* value	Adj. *p*-value
ANGPTL4	Homo sapiens angiopoietin-like 4, trans. var. 1.	3,003	9,836	7,283	3,391E-05	0,023
HBEGF	Homo sapiens heparin-binding EGF-like growth factor.	2,817	10,497	6,490	8,609E-05	0,034
TM4SF19	Homo sapiens transmembrane 4 L six family member 19.	2,673	10,466	6,072	1,450E-04	0,043
KLF6	Homo sapiens Kruppel-like factor 6, trans. var. 2.	2,465	11,162	7,797	1,925E-05	0,019
SERPINE1	Homo sapiens serpin peptidase inhibitor, clade E (nexin. plasminogen activator inhibitor type 1), member 1.	2,417	10,078	8,099	1,400E-05	0,017
CDKN1A	Homo sapiens cyclin-dependent kinase inhibitor 1A (p21. Cip1), trans. var. 1.	2,302	11,510	6,204	1,228E-04	0,040
KLF6	Homo sapiens Kruppel-like factor 6, trans. var. 1.	2,192	10,386	6,516	8,336E-05	0,034
RHOB	Homo sapiens ras homolog gene family. member B.	2,168	10,151	7,776	1,969E-05	0,019
KLF2	Homo sapiens Kruppel-like factor 2 (lung).	1,839	9,619	8,297	1,140E-05	0,017
PHLDA1	Homo sapiens pleckstrin homology-like domain, family A, member 1.	1,732	12,250	7,131	4,027E-05	0,024
PLAU	Homo sapiens plasminogen activator, urokinase.	1,614	12,748	8,829	6,703E-06	0,015
FOSL1	Homo sapiens FOS-like antigen 1.	1,603	10,368	6,876	5,418E-05	0,029
EMP1	Homo sapiens epithelial membrane protein 1.	1,563	10,788	7,559	2,494E-05	0,020
LAMB3	Homo sapiens laminin b-3, trans. var. 1 .	1,481	12,851	6,353	1,018E-04	0,037
BTG1	Homo sapiens B-cell translocation gene 1, anti-proliferative.	1,392	10,939	5,936	1,729E-04	0,046
DUSP5	Homo sapiens dual specificity phosphatase 5.	1,332	10,463	5,845	1,946E-04	0,047
UPP1	Homo sapiens uridine phosphorylase 1, trans. var. 1.	1,260	11,053	7,396	2,987E-05	0,022
SLC16A3	Homo sapiens solute carrier family 16, member 3 (monocarboxylic acid transporter 4), trans. var. 2.	1,225	8,747	6,153	1,309E-04	0,040
CITED4	Homo sapiens Cbp/p300-interacting transactivator, with Glu/Asp-rich carboxy-terminal domain 4.	1,199	9,997	6,195	1,241E-04	0,040
SLC20A1	Homo sapiens solute carrier family 20 (phosphate transporter), member 1.	1,192	11,409	8,172	1,297E-05	0,017
FLNB	Homo sapiens filamin B, b (actin binding protein 278).	1,174	10,391	8,528	9,025E-06	0,017
PLEK2	Homo sapiens pleckstrin 2.	1,168	10,009	5,830	1,985E-04	0,047
MIR1974	Homo sapiens microRNA 1974,. microRNA.	1,096	11,829	9,095	5,189E-06	0,015
TUBB2A	Homo sapiens tubulin b-2A.	1,072	9,847	6,016	1,559E-04	0,044
ETV5	Homo sapiens ets variant gene 5 (ets-related molecule).	1,068	9,080	5,949	1,699E-04	0,046
MYADM	Homo sapiens myeloid-associated differentiation marker, trans. var. 4.	1,058	9,420	7,098	4,186E-05	0,024
LCP1	Homo sapiens lymphocyte cytosolic protein 1 (L-plastin).	1,021	9,934	8,379	1,049E-05	0,017
BCL2L1	Homo sapiens BCL2-like 1 nuclear gene encoding mitochondrial protein, trans. var. 1.	1,003	11,438	6,150	1,315E-04	0,040
LYPD1	Homo sapiens LY6/PLAUR domain containing 1, trans. var. 1.	–1,012	7,873	–6,163	1,293E-04	0,040
SOX2	Homo sapiens SRY (sex determining region Y)-box 2.	–1,062	7,790	–10,857	1,097E-06	0,005
LOC100134073	PREDICTED: Homo sapiens similar to LYPDC1 protein.	–1,198	7,936	–5,783	2,110E-04	0,048
SOX2	Homo sapiens SRY (sex determining region Y)-box 2.	–1,203	7,906	–10,949	1,017E-06	0,005

Supplementary Table 3B: List of differentially regulated genes in HN-5 cells (IC50 vs control). See Supplementary_Table_3B

Supplementary Table 3C: List of differentially regulated genes in HN-5 cells (IC75 vs control). See Supplementary_Table_3C

Supplementary Table 4A: List of GO terms enriched in IC50 vs control. See Supplementary_Table_4A

Supplementary Table 4B: List of GO terms enriched in IC75 vs control. See Supplementary_Table_4B
