# Supplementary material for: Upregulation of cell cycle genes in head and neck cancer patients may be antagonized by erufosine’s down regulation of cell cycle processes in OSCC cells
Source: Oncotarget. 2017 Dec 20;9(5):5797–810. doi: 10.18632/oncotarget.23537 (PMC5814175; doi:10.18632/oncotarget.23537)
Supplement: Supplementary file 2 [file oncotarget-09-5797-s002.docx]

**Supplementary Table 3B: List of differentially regulated genes in HN-5 cells (IC50 vs control)**

| **Symbol** | **Definition** | **Log FC** | **Avg. Expression** | **t-statistics** | **p value** | **Adj. p-value** |
| --- | --- | --- | --- | --- | --- | --- |
| HBEGF | Homo sapiens heparin-binding EGF-like growth factor. | 3,938 | 10,497 | 9,074 | 5,296E-06 | 0,002 |
| KLF6 | Homo sapiens Kruppel-like factor 6, trans. var. 2. | 3,670 | 11,162 | 11,610 | 6,023E-07 | 0,001 |
| CDKN1A | Homo sapiens cyclin-dependent kinase inhibitor 1A (p21. Cip1), trans. var. 1. | 3,559 | 11,510 | 9,592 | 3,269E-06 | 0,002 |
| KLF6 | Homo sapiens Kruppel-like factor 6, trans. var. 1. | 3,447 | 10,386 | 10,246 | 1,831E-06 | 0,001 |
| ANGPTL4 | Homo sapiens angiopoietin-like 4, trans. var. 1. | 3,399 | 9,836 | 8,242 | 1,207E-05 | 0,003 |
| TM4SF19 | Homo sapiens transmembrane 4 L six family member 19. | 3,365 | 10,466 | 7,644 | 2,271E-05 | 0,003 |
| CSF2 | Homo sapiens colony stimulating factor 2 (granulocyte-macrophage). | 3,331 | 9,656 | 7,918 | 1,692E-05 | 0,003 |
| FAM83A | Homo sapiens family with sequence similarity 83, member A, trans. var. 1. | 3,298 | 9,386 | 4,124 | 2,275E-03 | 0,029 |
| FAM83A | Homo sapiens family with sequence similarity 83, member A, trans. var. 2. | 3,292 | 9,395 | 3,841 | 3,545E-03 | 0,037 |
| RHOB | Homo sapiens ras homolog gene family, member B. | 3,205 | 10,151 | 11,498 | 6,567E-07 | 0,001 |
| IL1B | Homo sapiens interleukin 1 β. | 3,118 | 11,040 | 4,138 | 2,226E-03 | 0,028 |
| MMP10 | Homo sapiens matrix metallopeptidase 10 (stromelysin 2). | 3,079 | 10,764 | 6,097 | 1,406E-04 | 0,007 |
| TRIB1 | Homo sapiens tribbles homolog 1 (Drosophila). | 3,063 | 10,400 | 7,640 | 2,283E-05 | 0,003 |
| TM4SF19 | PREDICTED: Homo sapiens transmembrane 4 L six family member 19 trans. var. 2. | 2,834 | 9,571 | 5,775 | 2,132E-04 | 0,009 |
| KLF2 | Homo sapiens Kruppel-like factor 2 (lung). | 2,813 | 9,619 | 12,689 | 2,702E-07 | 0,001 |
| ODC1 | Homo sapiens ornithine decarboxylase 1. | 2,798 | 11,914 | 8,274 | 1,167E-05 | 0,003 |
| LAMC2 | Homo sapiens laminin gamma 2, trans. var. 1. | 2,771 | 11,266 | 6,569 | 7,811E-05 | 0,006 |
| LOC100132564 | PREDICTED: Homo sapiens hypothetical protein LOC100132564. | 2,586 | 11,239 | 6,490 | 8,610E-05 | 0,006 |
| LAMC2 | Homo sapiens laminin gamma 2, trans. var. 2. | 2,581 | 9,593 | 5,856 | 1,917E-04 | 0,008 |
| LAMC2 | Homo sapiens laminin gamma 2, trans. var. 1. | 2,581 | 11,919 | 5,671 | 2,445E-04 | 0,009 |
| TRIB3 | Homo sapiens tribbles homolog 3 (Drosophila). | 2,565 | 9,958 | 6,862 | 5,506E-05 | 0,005 |
| LOC650517 | PREDICTED: Homo sapiens hypothetical LOC650517. | 2,544 | 10,736 | 4,932 | 6,801E-04 | 0,015 |
| LOC100008589 | Homo sapiens 28S ribosomal RNA, non-coding RNA. | 2,494 | 11,080 | 5,529 | 2,962E-04 | 0,010 |
| DUSP5 | Homo sapiens dual specificity phosphatase 5. | 2,481 | 10,463 | 10,881 | 1,075E-06 | 0,001 |
| PHLDA1 | Homo sapiens pleckstrin homology-like domain family A, member 1. | 2,445 | 12,250 | 10,068 | 2,138E-06 | 0,001 |
| SERPINE1 | Homo sapiens serpin peptidase inhibitor clade E (nexin. plasminogen activator inhibitor type 1) member 1. | 2,414 | 10,078 | 8,090 | 1,413E-05 | 0,003 |
| PPP1R15A | Homo sapiens protein phosphatase 1 regulatory (inhibitor) subunit 15A. | 2,354 | 10,980 | 7,737 | 2,054E-05 | 0,003 |
| PLAUR | Homo sapiens plasminogen activator, urokinase receptor trans. var. 2 . | 2,352 | 9,575 | 7,122 | 4,071E-05 | 0,004 |
| SERPINB1 | Homo sapiens serpin peptidase inhibitor clade B (ovalbumin), member 1. | 2,347 | 8,933 | 4,013 | 2,702E-03 | 0,032 |
| NDRG1 | Homo sapiens N-myc downstream regulated gene 1. | 2,330 | 12,379 | 5,724 | 2,280E-04 | 0,009 |
| LOC441019 | PREDICTED: Homo sapiens hypothetical LOC441019. | 2,215 | 11,194 | 3,992 | 2,793E-03 | 0,032 |
| SPRY2 | Homo sapiens sprouty homolog 2 (Drosophila). | 2,162 | 9,110 | 4,209 | 1,995E-03 | 0,027 |
| PLAU | Homo sapiens plasminogen activator, urokinase. | 2,152 | 12,748 | 11,775 | 5,303E-07 | 0,001 |
| BTG1 | Homo sapiens B-cell translocation gene 1, anti-proliferative. | 2,150 | 10,939 | 9,165 | 4,856E-06 | 0,002 |
| GJB3 | Homo sapiens gap junction protein, β-3. 31kDa, trans. var. 2. | 2,137 | 10,128 | 7,103 | 4,163E-05 | 0,004 |
| LAMB3 | Homo sapiens laminin. β-3, trans. var. 1 . | 2,123 | 12,851 | 9,104 | 5,148E-06 | 0,002 |
| ISG20 | Homo sapiens interferon stimulated exonuclease gene 20kDa. | 2,104 | 9,916 | 5,198 | 4,665E-04 | 0,012 |
| PLAUR | Homo sapiens plasminogen activator, urokinase receptor trans. var. 1. | 2,102 | 9,191 | 6,956 | 4,935E-05 | 0,004 |
| LOC100134364 | PREDICTED: Homo sapiens hypothetical protein LOC100134364. | 2,077 | 10,937 | 5,520 | 2,996E-04 | 0,010 |
| LOC100132394 | PREDICTED: Homo sapiens hypothetical protein LOC100132394. | 2,058 | 11,375 | 5,699 | 2,358E-04 | 0,009 |
| AKAP12 | Homo sapiens A kinase (PRKA) anchor protein (gravin) 12, trans. var. 2. | 2,046 | 8,908 | 7,361 | 3,107E-05 | 0,004 |
| LAMA3 | Homo sapiens laminin, alpha 3, trans. var. 1. | 2,045 | 9,735 | 4,589 | 1,122E-03 | 0,020 |
| NT5E | Homo sapiens 5'-nucleotidase, ecto (CD73). | 2,041 | 9,161 | 4,178 | 2,091E-03 | 0,027 |
| C14orf78 | PREDICTED: Homo sapiens chromosome 14 open reading frame 78. | 2,031 | 9,358 | 6,526 | 8,240E-05 | 0,006 |
| AHNAK2 | Homo sapiens AHNAK nucleoprotein 2. | 1,973 | 9,115 | 6,432 | 9,243E-05 | 0,006 |
| KRT17P3 | PREDICTED: Homo sapiens misc_RNA (KRT17P3), miscRNA. | 1,956 | 11,880 | 4,331 | 1,654E-03 | 0,024 |
| ITGA2 | Homo sapiens integrin, alpha 2 (CD49B. α-2 subunit of VLA-2 receptor). | 1,956 | 10,847 | 7,169 | 3,857E-05 | 0,004 |
| IER3 | Homo sapiens immediate early response 3. | 1,939 | 12,800 | 6,143 | 1,326E-04 | 0,007 |
| ERRFI1 | Homo sapiens ERBB receptor feedback inhibitor 1. | 1,930 | 10,536 | 6,823 | 5,763E-05 | 0,005 |
| ARHGEF2 | Homo sapiens rho/rac guanine nucleotide exchange factor (GEF) 2. | 1,923 | 10,199 | 8,545 | 8,872E-06 | 0,002 |
| IL1A | Homo sapiens interleukin 1 α. | 1,918 | 11,288 | 7,744 | 2,039E-05 | 0,003 |
| EMP1 | Homo sapiens epithelial membrane protein 1. | 1,913 | 10,788 | 9,250 | 4,483E-06 | 0,002 |
| UPP1 | Homo sapiens uridine phosphorylase 1, trans. var. 1. | 1,906 | 11,053 | 11,187 | 8,396E-07 | 0,001 |
| TGFA | Homo sapiens transforming growth factor α. | 1,897 | 9,581 | 6,233 | 1,183E-04 | 0,007 |
| LOC387841 | PREDICTED: Homo sapiens similar to ribosomal protein L13a trans. var. 2. | 1,879 | 9,807 | 5,226 | 4,485E-04 | 0,012 |
| ULK1 | Homo sapiens unc-51-like kinase 1 (C. elegans). | 1,878 | 10,446 | 5,183 | 4,761E-04 | 0,013 |
| MT2A | Homo sapiens metallothionein 2A. | 1,876 | 12,664 | 4,726 | 9,172E-04 | 0,018 |
| MALL | Homo sapiens mal. T-cell differentiation protein-like. | 1,861 | 10,350 | 6,043 | 1,506E-04 | 0,007 |
| ECGF1 | Homo sapiens endothelial cell growth factor 1 (platelet-derived). | 1,856 | 10,409 | 9,880 | 2,523E-06 | 0,001 |
| CGB5 | Homo sapiens chorionic gonadotropin β, polypeptide 5. | 1,842 | 8,909 | 5,578 | 2,771E-04 | 0,010 |
| SLC16A3 | Homo sapiens solute carrier family 16, member 3 (monocarboxylic acid transporter 4), trans. var. 2. | 1,835 | 8,747 | 9,218 | 4,620E-06 | 0,002 |
| FOSL1 | Homo sapiens FOS-like antigen 1. | 1,829 | 10,368 | 7,847 | 1,825E-05 | 0,003 |
| PIM1 | Homo sapiens pim-1 oncogene. | 1,822 | 9,120 | 8,422 | 1,004E-05 | 0,002 |
| LOC100008589 | Homo sapiens 28S ribosomal RNA, non-coding RNA. | 1,819 | 12,951 | 8,257 | 1,188E-05 | 0,003 |
| ASNS | Homo sapiens asparagine synthetase, trans. var. 1. | 1,806 | 11,287 | 4,099 | 2,363E-03 | 0,029 |
| SAT1 | Homo sapiens spermidine/spermine N1-acetyltransferase 1. | 1,805 | 11,071 | 6,786 | 6,023E-05 | 0,005 |
| LAMA3 | Homo sapiens laminin α-3, trans. var. 1 . | 1,804 | 9,026 | 3,890 | 3,279E-03 | 0,036 |
| ULK1 | Homo sapiens unc-51-like kinase 1 (C. elegans). | 1,799 | 10,155 | 4,898 | 7,136E-04 | 0,016 |
| RAC2 | Homo sapiens ras-related C3 botulinum toxin substrate 2 (rho family, small GTP binding protein Rac2). | 1,794 | 11,657 | 6,821 | 5,778E-05 | 0,005 |
| ANTXR2 | Homo sapiens anthrax toxin receptor 2. | 1,772 | 8,807 | 6,151 | 1,312E-04 | 0,007 |
| TNFRSF25 | Homo sapiens tumor necrosis factor receptor superfamily, member 25, trans. var. 10. | 1,751 | 9,359 | 5,450 | 3,293E-04 | 0,011 |
| TMBIM1 | Homo sapiens transmembrane BAX inhibitor motif containing 1. | 1,749 | 9,555 | 4,796 | 8,272E-04 | 0,017 |
| RAP1GAP | Homo sapiens RAP1 GTPase activating protein. | 1,746 | 9,367 | 7,783 | 1,955E-05 | 0,003 |
| PLEC1 | Homo sapiens plectin 1, intermediate filament binding protein 500kDa, trans. var. 1. | 1,739 | 10,640 | 6,124 | 1,358E-04 | 0,007 |
| FLNB | Homo sapiens filamin B. beta (actin binding protein 278). | 1,735 | 10,391 | 12,604 | 2,871E-07 | 0,001 |
| GRB7 | Homo sapiens growth factor receptor-bound protein 7, trans. var. 2. | 1,729 | 9,219 | 7,140 | 3,987E-05 | 0,004 |
| KRT16 | Homo sapiens keratin 16 (focal non-epidermolytic palmoplantar keratoderma). | 1,722 | 11,085 | 3,620 | 5,051E-03 | 0,046 |
| MYADM | Homo sapiens myeloid-associated differentiation marker, trans. var. 4. | 1,721 | 9,420 | 11,550 | 6,306E-07 | 0,001 |
| CDCP1 | Homo sapiens CUB domain containing protein 1, trans. var. 1. | 1,714 | 9,314 | 6,958 | 4,919E-05 | 0,004 |
| TUBB2A | Homo sapiens tubulin beta 2A. | 1,709 | 9,847 | 9,586 | 3,286E-06 | 0,002 |
| KCNK1 | Homo sapiens potassium channel, subfamily K, member 1. | 1,700 | 9,743 | 4,881 | 7,322E-04 | 0,016 |
| FERMT1 | Homo sapiens fermitin family homolog 1 (Drosophila). | 1,690 | 10,082 | 4,867 | 7,470E-04 | 0,016 |
| DUSP6 | Homo sapiens dual specificity phosphatase 6, trans. var. 2. | 1,668 | 9,857 | 5,046 | 5,775E-04 | 0,014 |
| TGFA | Homo sapiens transforming growth factor α, trans. var. 2. | 1,665 | 9,149 | 5,999 | 1,593E-04 | 0,007 |
| ASNS | Homo sapiens asparagine synthetase, trans. var. 1. | 1,657 | 9,480 | 4,753 | 8,819E-04 | 0,017 |
| KRT17 | Homo sapiens keratin 17. | 1,654 | 12,238 | 4,422 | 1,440E-03 | 0,022 |
| MTSS1 | Homo sapiens metastasis suppressor 1. | 1,634 | 9,941 | 7,420 | 2,907E-05 | 0,004 |
| KCNK1 | Homo sapiens potassium channel, subfamily K, member 1. | 1,632 | 9,239 | 4,183 | 2,075E-03 | 0,027 |
| SPRR1A | Homo sapiens small proline-rich protein 1A. | 1,627 | 8,594 | 5,292 | 4,093E-04 | 0,012 |
| CAPRIN2 | Homo sapiens caprin family member 2, trans. var. 1. | 1,626 | 9,742 | 6,113 | 1,378E-04 | 0,007 |
| MMP9 | Homo sapiens matrix metallopeptidase 9 (gelatinase B, 92kDa gelatinase, 92kDa type IV collagenase). | 1,619 | 8,841 | 4,285 | 1,774E-03 | 0,025 |
| CITED4 | Homo sapiens Cbp/p300-interacting transactivator, with Glu/Asp-rich carboxy-terminal domain 4. | 1,612 | 9,997 | 8,329 | 1,104E-05 | 0,003 |
| MXD1 | Homo sapiens MAX dimerization protein 1. | 1,597 | 8,665 | 7,530 | 2,575E-05 | 0,004 |
| SH3PXD2A | Homo sapiens SH3 and PX domains 2A. | 1,583 | 10,304 | 4,782 | 8,453E-04 | 0,017 |
| SELS | Homo sapiens selenoprotein S, trans. var. 2. | 1,578 | 10,875 | 10,475 | 1,507E-06 | 0,001 |
| PHLDA1 | Homo sapiens pleckstrin homology-like domain, family A. member 1. | 1,571 | 8,780 | 4,240 | 1,902E-03 | 0,026 |
| STX1A | Homo sapiens syntaxin 1A (brain). | 1,567 | 8,663 | 6,423 | 9,344E-05 | 0,006 |
| S100A6 | Homo sapiens S100 calcium binding protein A6. | 1,567 | 11,316 | 3,795 | 3,816E-03 | 0,039 |
| PLEK2 | Homo sapiens pleckstrin 2. | 1,561 | 10,009 | 7,790 | 1,940E-05 | 0,003 |
| DUSP1 | Homo sapiens dual specificity phosphatase 1. | 1,560 | 9,044 | 6,835 | 5,682E-05 | 0,005 |
| LOC653506 | PREDICTED: Homo sapiens similar to meteorin, glial cell differentiation regulator-like. | 1,558 | 8,703 | 6,381 | 9,844E-05 | 0,006 |
| IRF9 | Homo sapiens interferon regulatory factor 9. | 1,555 | 8,952 | 5,047 | 5,773E-04 | 0,014 |
| CD68 | Homo sapiens CD68 antigen. | 1,545 | 10,183 | 3,638 | 4,907E-03 | 0,045 |
| BCAR3 | Homo sapiens breast cancer anti-estrogen resistance 3. | 1,545 | 10,164 | 6,043 | 1,505E-04 | 0,007 |
| CGB1 | Homo sapiens chorionic gonadotropin, beta polypeptide 1. | 1,538 | 8,681 | 5,260 | 4,277E-04 | 0,012 |
| WIPI1 | Homo sapiens WD repeat domain, phosphoinositide interacting 1. | 1,534 | 8,744 | 8,905 | 6,231E-06 | 0,002 |
| BHLHB2 | Homo sapiens basic helix-loop-helix domain containing, class B. 2. | 1,531 | 11,006 | 5,460 | 3,251E-04 | 0,010 |
| LOC100008588 | Homo sapiens 18S ribosomal RNA, non-coding RNA. | 1,526 | 11,305 | 4,187 | 2,064E-03 | 0,027 |
| MIR1974 | Homo sapiens microRNA 1974, microRNA. | 1,519 | 11,829 | 12,597 | 2,884E-07 | 0,001 |
| CEBPB | Homo sapiens CCAAT/enhancer binding protein (C/EBP)-β. | 1,501 | 12,103 | 7,743 | 2,040E-05 | 0,003 |
| LARP6 | Homo sapiens La ribonucleoprotein domain family, member 6, trans. var. 1. | 1,500 | 9,085 | 8,458 | 9,683E-06 | 0,002 |
| C5orf32 | Homo sapiens chromosome 5 open reading frame 32. | 1,498 | 9,239 | 5,672 | 2,445E-04 | 0,009 |
| OAF | Homo sapiens OAF homolog (Drosophila). | 1,486 | 9,682 | 4,421 | 1,444E-03 | 0,022 |
| PLAUR | Homo sapiens plasminogen activator, urokinase receptor, trans. var. 2. | 1,481 | 8,653 | 4,515 | 1,253E-03 | 0,021 |
| CSNK1E | Homo sapiens casein kinase 1 epsilon, trans. var. 1. | 1,473 | 10,624 | 8,549 | 8,842E-06 | 0,002 |
| IL11 | Homo sapiens interleukin 11. | 1,466 | 8,668 | 6,104 | 1,392E-04 | 0,007 |
| CPXM1 | Homo sapiens carboxypeptidase X (M14 family) member 1. | 1,457 | 8,893 | 5,199 | 4,659E-04 | 0,012 |
| JUN | Homo sapiens jun oncogene. | 1,452 | 10,798 | 7,102 | 4,164E-05 | 0,004 |
| OPLAH | Homo sapiens 5-oxoprolinase (ATP-hydrolysing). | 1,448 | 8,920 | 9,356 | 4,061E-06 | 0,002 |
| HPCAL1 | Homo sapiens hippocalcin-like 1, trans. var. 2. | 1,441 | 10,708 | 6,877 | 5,414E-05 | 0,005 |
| ZBED2 | Homo sapiens zinc finger, BED-type containing 2. | 1,428 | 8,840 | 3,670 | 4,658E-03 | 0,044 |
| F3 | Homo sapiens coagulation factor III (thromboplastin, tissue factor). | 1,428 | 10,706 | 4,860 | 7,547E-04 | 0,016 |
| IRF6 | Homo sapiens interferon regulatory factor 6. | 1,427 | 10,250 | 6,758 | 6,228E-05 | 0,005 |
| AHNAK | Homo sapiens AHNAK nucleoprotein, trans. var. 1. | 1,427 | 10,469 | 4,536 | 1,215E-03 | 0,020 |
| LRRC8A | Homo sapiens leucine rich repeat containing 8 family, member A. | 1,423 | 9,387 | 8,527 | 9,037E-06 | 0,002 |
| ANXA3 | Homo sapiens annexin A3. | 1,422 | 9,516 | 6,165 | 1,289E-04 | 0,007 |
| SPRY4 | Homo sapiens sprouty homolog 4 (Drosophila). | 1,419 | 8,234 | 5,344 | 3,812E-04 | 0,011 |
| ZFP36L2 | Homo sapiens zinc finger protein 36, C3H type-like 2. | 1,409 | 8,887 | 4,821 | 7,988E-04 | 0,016 |
| PCK2 | Homo sapiens phosphoenolpyruvate carboxykinase 2 (mitochondrial), nuclear gene encoding mitochondrial protein, trans. var. 1. | 1,408 | 8,858 | 3,888 | 3,289E-03 | 0,036 |
| NET1 | Homo sapiens neuroepithelial cell transforming 1, trans. var. 1. | 1,405 | 11,644 | 6,066 | 1,461E-04 | 0,007 |
| LCP1 | Homo sapiens lymphocyte cytosolic protein 1 (L-plastin). | 1,399 | 9,934 | 11,481 | 6,656E-07 | 0,001 |
| TSC22D1 | Homo sapiens TSC22 domain family member 1, trans. var. 2 | 1,399 | 10,135 | 4,363 | 1,576E-03 | 0,024 |
| ITPR3 | Homo sapiens inositol 1.4.5-triphosphate receptor, type 3. | 1,392 | 10,616 | 4,232 | 1,926E-03 | 0,026 |
| ATP9A | Homo sapiens ATPase, class II, type 9A. | 1,391 | 10,079 | 7,842 | 1,836E-05 | 0,003 |
| BCL2L1 | Homo sapiens BCL2-like 1, nuclear gene encoding mitochondrial protein trans. var. 1. | 1,386 | 11,438 | 8,499 | 9,293E-06 | 0,002 |
| SPHK1 | Homo sapiens sphingosine kinase 1, trans. var. 1. | 1,386 | 8,630 | 5,740 | 2,232E-04 | 0,009 |
| S100A16 | Homo sapiens S100 calcium binding protein A16. | 1,383 | 11,239 | 5,531 | 2,951E-04 | 0,010 |
| C1orf106 | Homo sapiens chromosome 1 open reading frame 106. | 1,381 | 9,543 | 5,262 | 4,264E-04 | 0,012 |
| NDEL1 | Homo sapiens nudE nuclear distribution gene E homolog (A. nidulans)-like 1, trans. var. 2. | 1,380 | 10,315 | 5,389 | 3,581E-04 | 0,011 |
| METRNL | PREDICTED: Homo sapiens meteorin, glial cell differentiation regulator-like. | 1,378 | 8,502 | 6,106 | 1,390E-04 | 0,007 |
| LIMA1 | Homo sapiens LIM domain and actin binding 1. | 1,377 | 10,187 | 5,941 | 1,718E-04 | 0,008 |
| SELS | Homo sapiens selenoprotein S (SELS), trans. var. 2. | 1,377 | 9,469 | 9,268 | 4,408E-06 | 0,002 |
| RHOC | Homo sapiens ras homolog gene family, member C, trans. var. 1. | 1,373 | 11,856 | 8,659 | 7,925E-06 | 0,002 |
| KLC3 | Homo sapiens kinesin light chain 3, trans. var. 1. | 1,365 | 8,839 | 5,484 | 3,145E-04 | 0,010 |
| HERPUD1 | Homo sapiens homocysteine-inducible, endoplasmic reticulum stress-inducible, ubiquitin-like domain member 1, trans. var. 3. | 1,363 | 9,744 | 7,438 | 2,850E-05 | 0,004 |
| TSC22D1 | Homo sapiens TSC22 domain family member 1, trans. var. 2. | 1,355 | 11,099 | 4,776 | 8,517E-04 | 0,017 |
| SH3KBP1 | Homo sapiens SH3-domain kinase binding protein 1, trans. var. 1. | 1,353 | 9,837 | 6,154 | 1,308E-04 | 0,007 |
| CLIC4 | Homo sapiens chloride intracellular channel 4, nuclear gene encoding mitochondrial protein. | 1,350 | 9,564 | 7,688 | 2,166E-05 | 0,003 |
| ACSS2 | Homo sapiens acyl-CoA synthetase short-chain family member 2 trans. var. 1. | 1,347 | 9,381 | 5,635 | 2,567E-04 | 0,009 |
| ITGB4 | Homo sapiens integrin. β-4, trans. var. 2. | 1,347 | 11,228 | 8,390 | 1,037E-05 | 0,002 |
| GNA15 | Homo sapiens guanine nucleotide binding protein (G protein). α15 (Gq class). | 1,346 | 9,645 | 6,972 | 4,842E-05 | 0,004 |
| ITGA5 | Homo sapiens integrin α-5 (fibronectin receptor. α-polypeptide). | 1,345 | 8,861 | 3,868 | 3,398E-03 | 0,036 |
| HERPUD1 | Homo sapiens homocysteine-inducible, endoplasmic reticulum stress-inducible, ubiquitin-like domain member 1, trans. var. 3. | 1,342 | 9,852 | 7,605 | 2,370E-05 | 0,003 |
| ACSS2 | Homo sapiens acyl-CoA synthetase short-chain family member 2, trans. var. 2 | 1,341 | 9,184 | 5,487 | 3,133E-04 | 0,010 |
| CAPRIN2 | Homo sapiens caprin family member 2, trans. var. 2. | 1,336 | 8,868 | 5,089 | 5,441E-04 | 0,013 |
| RIOK3 | Homo sapiens RIO kinase 3 (yeast). | 1,336 | 9,922 | 6,468 | 8,843E-05 | 0,006 |
| NPC1 | Homo sapiens Niemann-Pick disease, type C1. | 1,334 | 9,255 | 7,277 | 3,414E-05 | 0,004 |
| TMEM154 | Homo sapiens transmembrane protein 154. | 1,327 | 8,833 | 8,078 | 1,430E-05 | 0,003 |
| SH3BGRL3 | Homo sapiens SH3 domain binding glutamic acid-rich protein like 3. | 1,327 | 11,011 | 4,472 | 1,336E-03 | 0,022 |
| FAM84B | Homo sapiens family with sequence similarity 84, member B. | 1,318 | 10,189 | 5,915 | 1,775E-04 | 0,008 |
| ANXA2P1 | Homo sapiens annexin A2 pseudogene 1 on chromosome 4. | 1,310 | 11,728 | 6,347 | 1,027E-04 | 0,006 |
| SOX9 | Homo sapiens SRY (sex determining region Y)-box 9 (campomelic dysplasia. autosomal sex-reversal). | 1,298 | 9,079 | 3,953 | 2,972E-03 | 0,034 |
| CSNK1E | Homo sapiens casein kinase 1, epsilon, trans. var. 1. | 1,294 | 9,570 | 9,002 | 5,672E-06 | 0,002 |
| SLC20A1 | Homo sapiens solute carrier family 20 (phosphate transporter), member 1. | 1,294 | 11,409 | 8,867 | 6,462E-06 | 0,002 |
| RNF19B | Homo sapiens ring finger protein 19B. | 1,293 | 9,329 | 5,626 | 2,597E-04 | 0,009 |
| TSC22D3 | Homo sapiens TSC22 domain family member 3, trans. var. 1 | 1,293 | 9,224 | 5,852 | 1,926E-04 | 0,008 |
| EPAS1 | Homo sapiens endothelial PAS domain protein 1. | 1,286 | 9,724 | 5,447 | 3,309E-04 | 0,011 |
| ABCC3 | Homo sapiens ATP-binding cassette, sub-family C (CFTR/MRP), member 3. | 1,280 | 9,183 | 4,909 | 7,026E-04 | 0,015 |
| NP | Homo sapiens nucleoside phosphorylase. | 1,277 | 9,635 | 4,655 | 1,018E-03 | 0,018 |
| C12orf35 | Homo sapiens chromosome 12 open reading frame 35. | 1,275 | 8,997 | 3,865 | 3,412E-03 | 0,036 |
| PGM3 | Homo sapiens phosphoglucomutase 3. | 1,273 | 9,670 | 11,883 | 4,886E-07 | 0,001 |
| LRRC8C | Homo sapiens leucine rich repeat containing 8 family, member C. | 1,272 | 8,820 | 4,475 | 1,331E-03 | 0,021 |
| VIM | Homo sapiens vimentin. | 1,272 | 11,382 | 5,023 | 5,973E-04 | 0,014 |
| SMOX | Homo sapiens spermine oxidase, trans. var. 2. | 1,269 | 8,517 | 4,942 | 6,707E-04 | 0,015 |
| CD68 | Homo sapiens CD68 molecule, trans. var. 1. | 1,264 | 9,292 | 4,370 | 1,559E-03 | 0,023 |
| IRAK2 | Homo sapiens interleukin-1 receptor-associated kinase 2. | 1,263 | 8,552 | 5,319 | 3,942E-04 | 0,011 |
| NET1 | Homo sapiens neuroepithelial cell transforming 1, trans. var. 2. | 1,262 | 9,342 | 5,289 | 4,109E-04 | 0,012 |
| ZFP36 | Homo sapiens zinc finger protein 36, C3H type, homolog (mouse). | 1,260 | 9,957 | 5,253 | 4,323E-04 | 0,012 |
| TIMP1 | Homo sapiens TIMP metallopeptidase inhibitor 1. | 1,257 | 10,413 | 5,587 | 2,737E-04 | 0,010 |
| RRAS | Homo sapiens related RAS viral (r-ras) oncogene homolog. | 1,245 | 10,023 | 5,572 | 2,794E-04 | 0,010 |
| ITGB4 | Homo sapiens integrin beta 4, trans. var. 3. | 1,235 | 10,265 | 7,926 | 1,678E-05 | 0,003 |
| TMC6 | Homo sapiens transmembrane channel-like 6. | 1,231 | 8,474 | 5,351 | 3,773E-04 | 0,011 |
| S100A2 | Homo sapiens S100 calcium binding protein A2. | 1,230 | 11,005 | 4,480 | 1,321E-03 | 0,021 |
| KIAA1539 | Homo sapiens KIAA1539. | 1,226 | 8,363 | 5,885 | 1,846E-04 | 0,008 |
| VIM | Homo sapiens vimentin. | 1,222 | 12,173 | 4,723 | 9,209E-04 | 0,018 |
| ZFP36L1 | Homo sapiens zinc finger protein 36. C3H type-like 1. | 1,219 | 9,634 | 6,260 | 1,143E-04 | 0,007 |
| RHOC | Homo sapiens ras homolog gene family member C, transcript variant 2 | 1,216 | 10,122 | 4,969 | 6,451E-04 | 0,015 |
| SPIRE1 | Homo sapiens spire homolog 1 (Drosophila), trans. var. 2. | 1,213 | 10,431 | 6,799 | 5,934E-05 | 0,005 |
| PPP2R2C | Homo sapiens protein phosphatase 2 (formerly 2A), regulatory subunit B. gamma isoform, trans. var. 2. | 1,212 | 8,718 | 4,895 | 7,175E-04 | 0,016 |
| IL8 | Homo sapiens interleukin 8. | 1,212 | 8,672 | 6,241 | 1,172E-04 | 0,007 |
| RIOK3 | Homo sapiens RIO kinase 3 (yeast), trans. var. 1. | 1,211 | 9,804 | 5,013 | 6,054E-04 | 0,014 |
| KIAA1949 | Homo sapiens KIAA1949. | 1,209 | 9,885 | 5,667 | 2,459E-04 | 0,009 |
| HMGA1 | Homo sapiens high mobility group AT-hook 1, trans. var. 1. | 1,206 | 12,001 | 3,720 | 4,301E-03 | 0,042 |
| AMTN | Homo sapiens amelotin. | 1,206 | 8,409 | 5,339 | 3,837E-04 | 0,011 |
| ITGA3 | Homo sapiens integrin α-3 (antigen CD49C. alpha 3 subunit of VLA-3 receptor), trans. var. a. | 1,201 | 11,439 | 7,243 | 3,545E-05 | 0,004 |
| PGM3 | Homo sapiens phosphoglucomutase 3. | 1,197 | 9,085 | 11,531 | 6,404E-07 | 0,001 |
| ATP2B4 | Homo sapiens ATPase. Ca++ transporting, plasma membrane 4, trans. var. 2. | 1,194 | 8,751 | 6,399 | 9,623E-05 | 0,006 |
| TMEM44 | Homo sapiens transmembrane protein 44, trans. var. 1. | 1,194 | 8,669 | 4,479 | 1,322E-03 | 0,021 |
| PTPN12 | Homo sapiens protein tyrosine phosphatase, non-receptor type 12. | 1,182 | 9,652 | 4,958 | 6,549E-04 | 0,015 |
| KLF11 | PREDICTED: Homo sapiens Kruppel-like factor 11. | 1,177 | 8,866 | 4,757 | 8,768E-04 | 0,017 |
| SH3KBP1 | Homo sapiens SH3-domain kinase binding protein 1, trans. var. 1. | 1,175 | 9,478 | 6,057 | 1,479E-04 | 0,007 |
| FXYD5 | Homo sapiens FXYD domain containing ion transport regulator 5, trans. var. 2 | 1,173 | 12,429 | 3,878 | 3,342E-03 | 0,036 |
| P4HA2 | Homo sapiens prolyl 4-hydroxylase, α-polypeptide II, trans. var. 2. | 1,171 | 10,806 | 5,071 | 5,574E-04 | 0,014 |
| GFPT1 | Homo sapiens glutamine-fructose-6-phosphate transaminase 1. | 1,170 | 9,083 | 12,078 | 4,219E-07 | 0,001 |
| TNFAIP3 | Homo sapiens tumor necrosis factor α-induced protein 3. | 1,162 | 8,502 | 5,723 | 2,284E-04 | 0,009 |
| ATP2B4 | Homo sapiens ATPase. Ca++ transporting. plasma membrane 4, trans. var. 1 . | 1,159 | 8,785 | 5,940 | 1,719E-04 | 0,008 |
| PYGB | "Homo sapiens phosphorylase, glycogen; brain. | 1,159 | 9,904 | 5,309 | 3,996E-04 | 0,012 |
| C13orf15 | Homo sapiens chromosome 13 open reading frame 15. | 1,158 | 8,564 | 6,410 | 9,489E-05 | 0,006 |
| GSK3B | Homo sapiens glycogen synthase kinase 3 β. | 1,150 | 9,077 | 4,113 | 2,314E-03 | 0,029 |
| BPGM | Homo sapiens 2.3-bisphosphoglycerate mutase, trans. var. 1. | 1,145 | 8,481 | 3,857 | 3,454E-03 | 0,037 |
| IRS2 | Homo sapiens insulin receptor substrate 2. | 1,143 | 9,049 | 4,224 | 1,947E-03 | 0,026 |
| EHD1 | Homo sapiens EH-domain containing 1. | 1,142 | 9,610 | 7,872 | 1,777E-05 | 0,003 |
| HAS3 | Homo sapiens hyaluronan synthase 3, trans. var. 1. | 1,139 | 9,971 | 5,836 | 1,968E-04 | 0,008 |
| SH2D5 | PREDICTED: Homo sapiens SH2 domain containing 5. | 1,139 | 8,273 | 5,529 | 2,959E-04 | 0,010 |
| MTSS1 | Homo sapiens metastasis suppressor 1 (MTSS1). | 1,136 | 8,394 | 6,571 | 7,798E-05 | 0,006 |
| GFPT1 | Homo sapiens glutamine-fructose-6-phosphate transaminase 1. | 1,133 | 9,306 | 13,426 | 1,617E-07 | 0,001 |
| NIPA1 | Homo sapiens non imprinted in Prader-Willi/Angelman syndrome 1. | 1,131 | 8,602 | 4,566 | 1,161E-03 | 0,020 |
| NAGK | Homo sapiens N-acetylglucosamine kinase. | 1,129 | 8,926 | 7,070 | 4,320E-05 | 0,004 |
| GLIPR1 | Homo sapiens GLI pathogenesis-related 1. | 1,125 | 8,351 | 8,863 | 6,484E-06 | 0,002 |
| FAM129B | Homo sapiens family with sequence similarity 129, member B, trans. var. 1. | 1,125 | 11,628 | 4,553 | 1,184E-03 | 0,020 |
| TGFB1I1 | Homo sapiens transforming growth factor β1 induced transcript 1, trans. var. 2 | 1,118 | 8,346 | 7,588 | 2,415E-05 | 0,003 |
| AKAP12 | Homo sapiens A kinase (PRKA) anchor protein (gravin) 12, trans. var. 1. | 1,111 | 8,036 | 4,939 | 6,730E-04 | 0,015 |
| LOC729768 | PREDICTED: Homo sapiens misc_RNA, miscRNA. | 1,109 | 11,673 | 8,477 | 9,499E-06 | 0,002 |
| JUP | Homo sapiens junction plakoglobin, trans. var. 1. | 1,105 | 11,848 | 6,213 | 1,213E-04 | 0,007 |
| DGKA | Homo sapiens diacylglycerol kinase- α, 80kDa, trans. var. 4. | 1,105 | 8,866 | 3,885 | 3,304E-03 | 0,036 |
| MCL1 | Homo sapiens myeloid cell leukemia sequence 1 (BCL2-related), trans. var. 1. | 1,103 | 9,721 | 5,976 | 1,640E-04 | 0,008 |
| DNAJB2 | Homo sapiens DnaJ (Hsp40) homolog, subfamily B. member 2, trans. var. 2. | 1,097 | 10,656 | 4,385 | 1,524E-03 | 0,023 |
| CHIC2 | Homo sapiens cysteine-rich hydrophobic domain 2. | 1,096 | 9,640 | 5,458 | 3,258E-04 | 0,010 |
| P4HA2 | Homo sapiens prolyl 4-hydroxylase, alpha polypeptide I, trans. var. 3. | 1,095 | 9,746 | 4,414 | 1,458E-03 | 0,022 |
| SMOX | Homo sapiens spermine oxidase (SMOX) trans. var. 4. | 1,093 | 8,407 | 4,698 | 9,556E-04 | 0,018 |
| KIAA0363 | PREDICTED: Homo sapiens KIAA0363 protein. | 1,091 | 8,031 | 8,512 | 9,168E-06 | 0,002 |
| LOC143666 | PREDICTED: Homo sapiens hypothetical protein LOC143666. | 1,091 | 8,247 | 7,321 | 3,247E-05 | 0,004 |
| F3 | Homo sapiens coagulation factor III (thromboplastin. tissue factor). | 1,088 | 10,180 | 4,093 | 2,387E-03 | 0,030 |
| DAB2 | Homo sapiens disabled homolog 2, mitogen-responsive phosphoprotein (Drosophila). | 1,085 | 8,301 | 4,038 | 2,598E-03 | 0,031 |
| GADD45A | Homo sapiens growth arrest and DNA-damage-inducible α. | 1,083 | 9,896 | 5,133 | 5,112E-04 | 0,013 |
| GADD45A | Homo sapiens growth arrest and DNA-damage-inducible α. | 1,082 | 9,613 | 4,662 | 1,008E-03 | 0,018 |
| KCNK6 | Homo sapiens potassium channel, subfamily K, member 6. | 1,079 | 8,869 | 8,365 | 1,064E-05 | 0,002 |
| PTPN12 | Homo sapiens protein tyrosine phosphatase, non-receptor type 12. | 1,079 | 9,535 | 4,987 | 6,281E-04 | 0,015 |
| ETV5 | Homo sapiens ets variant gene 5 (ets-related molecule). | 1,078 | 9,080 | 6,003 | 1,585E-04 | 0,007 |
| ANXA2P1 | Homo sapiens annexin A2 pseudogene 1 on chromosome 4. | 1,077 | 9,004 | 4,112 | 2,316E-03 | 0,029 |
| KLF13 | Homo sapiens Kruppel-like factor 13. | 1,076 | 9,205 | 4,629 | 1,057E-03 | 0,019 |
| ARFGAP3 | Homo sapiens ADP-ribosylation factor GTPase activating protein 3. | 1,073 | 9,222 | 8,187 | 1,277E-05 | 0,003 |
| FOXD1 | Homo sapiens forkhead box D1. | 1,070 | 8,726 | 4,353 | 1,599E-03 | 0,024 |
| VIL2 | Homo sapiens villin 2 (ezrin). | 1,067 | 12,483 | 10,575 | 1,385E-06 | 0,001 |
| KDELR3 | Homo sapiens KDEL (Lys-Asp-Glu-Leu) endoplasmic reticulum protein retention receptor 3, trans. var. 2. | 1,066 | 8,939 | 5,079 | 5,512E-04 | 0,014 |
| KLF9 | Homo sapiens Kruppel-like factor 9. | 1,063 | 8,544 | 3,566 | 5,511E-03 | 0,049 |
| C9orf21 | Homo sapiens chromosome 9 open reading frame 21. | 1,063 | 8,817 | 4,076 | 2,450E-03 | 0,030 |
| PTPRE | Homo sapiens protein tyrosine phosphatase. receptor type. E, trans. var. 2. | 1,056 | 8,474 | 7,530 | 2,575E-05 | 0,004 |
| GPSM1 | PREDICTED: Homo sapiens G-protein signalling modulator 1 (AGS3-like. C. elegans). | 1,054 | 8,098 | 6,775 | 6,102E-05 | 0,005 |
| XBP1 | Homo sapiens X-box binding protein 1, trans. var. 1. | 1,051 | 10,855 | 6,712 | 6,579E-05 | 0,005 |
| ETS1 | Homo sapiens v-ets erythroblastosis virus E26 oncogene homolog 1 (avian). | 1,050 | 8,998 | 7,161 | 3,893E-05 | 0,004 |
| STC2 | Homo sapiens stanniocalcin 2. | 1,047 | 8,973 | 6,044 | 1,503E-04 | 0,007 |
| GARS | Homo sapiens glycyl-tRNA synthetase. | 1,045 | 12,352 | 6,156 | 1,304E-04 | 0,007 |
| GJB5 | Homo sapiens gap junction protein, beta 5. 31.1kDa. | 1,038 | 8,257 | 5,626 | 2,599E-04 | 0,009 |
| PDLIM7 | Homo sapiens PDZ and LIM domain 7 (enigma), trans. var. 4. | 1,037 | 9,343 | 3,606 | 5,164E-03 | 0,047 |
| CD55 | Homo sapiens CD55 molecule, decay accelerating factor for complement (Cromer blood group). | 1,035 | 8,117 | 5,902 | 1,807E-04 | 0,008 |
| TMEM17 | Homo sapiens transmembrane protein 17. | 1,034 | 10,839 | 3,601 | 5,212E-03 | 0,047 |
| LOC100130154 | PREDICTED: Homo sapiens similar to thymosin. β-10. | 1,034 | 9,010 | 5,172 | 4,841E-04 | 0,013 |
| FAM129B | Homo sapiens family with sequence similarity 129. member B, trans. var. 2 . | 1,033 | 9,675 | 5,126 | 5,158E-04 | 0,013 |
| MAP1LC3B | Homo sapiens microtubule-associated protein 1 light chain 3-β. | 1,033 | 9,021 | 7,435 | 2,859E-05 | 0,004 |
| TRIM8 | Homo sapiens tripartite motif-containing 8. | 1,031 | 10,104 | 5,569 | 2,804E-04 | 0,010 |
| FOXO3 | Homo sapiens forkhead box O3, trans. var. 2. | 1,029 | 10,228 | 5,470 | 3,205E-04 | 0,010 |
| CDK5R1 | Homo sapiens cyclin-dependent kinase 5. regulatory subunit 1 (p35). | 1,027 | 8,798 | 4,163 | 2,140E-03 | 0,028 |
| CLDN12 | Homo sapiens claudin 12. | 1,025 | 8,995 | 3,699 | 4,451E-03 | 0,043 |
| TMEM154 | Homo sapiens transmembrane protein 154. | 1,025 | 8,461 | 6,491 | 8,599E-05 | 0,006 |
| FAM20C | Homo sapiens family with sequence similarity 20. member C. | 1,024 | 8,583 | 3,616 | 5,085E-03 | 0,046 |
| OXSR1 | Homo sapiens oxidative-stress responsive 1. | 1,023 | 10,044 | 5,083 | 5,481E-04 | 0,013 |
| S100A11 | Homo sapiens S100 calcium binding protein A11. | 1,022 | 11,272 | 4,239 | 1,903E-03 | 0,026 |
| XBP1 | Homo sapiens X-box binding protein 1, trans. var. 2. | 1,020 | 10,794 | 5,359 | 3,733E-04 | 0,011 |
| YPEL5 | Homo sapiens yippee-like 5 (Drosophila). | 1,020 | 9,746 | 5,266 | 4,243E-04 | 0,012 |
| LOC730278 | PREDICTED: Homo sapiens hypothetical LOC730278. | 1,016 | 12,563 | 4,594 | 1,114E-03 | 0,020 |
| LRP10 | Homo sapiens low density lipoprotein receptor-related protein 10. | 1,015 | 10,120 | 5,059 | 5,677E-04 | 0,014 |
| CLCF1 | Homo sapiens cardiotrophin-like cytokine factor 1, transcript variant 1 | 1,014 | 8,470 | 4,409 | 1,469E-03 | 0,023 |
| SLC38A2 | Homo sapiens solute carrier family 38, member 2. | 1,013 | 10,029 | 5,471 | 3,202E-04 | 0,010 |
| TMEM16A | Homo sapiens transmembrane protein 16A. | 1,012 | 9,303 | 3,918 | 3,136E-03 | 0,035 |
| LOC399748 | PREDICTED: Homo sapiens misc_RNA, miscRNA. | 1,010 | 11,095 | 6,574 | 7,771E-05 | 0,006 |
| KDELR3 | Homo sapiens KDEL (Lys-Asp-Glu-Leu) endoplasmic reticulum protein retention receptor 3, trans. var. 1. | 1,010 | 8,693 | 7,219 | 3,645E-05 | 0,004 |
| SOCS1 | Homo sapiens suppressor of cytokine signaling 1. | 1,003 | 7,937 | 3,631 | 4,965E-03 | 0,046 |
| ADRB2 | Homo sapiens adrenergic β-2-. Receptor, surface. | 1,001 | 8,232 | 4,439 | 1,405E-03 | 0,022 |
| NASP | Homo sapiens nuclear autoantigenic sperm protein (histone-binding), trans. var. 2. | -1,002 | 7,808 | -9,133 | 5,007E-06 | 0,002 |
| CYBASC3 | Homo sapiens cytochrome b. ascorbate dependent 3. | -1,006 | 8,498 | -4,532 | 1,222E-03 | 0,020 |
| LOC100132299 | PREDICTED: Homo sapiens similar to MSTP075. | -1,010 | 9,064 | -5,501 | 3,074E-04 | 0,010 |
| DNMT1 | Homo sapiens DNA (cytosine-5-)-methyltransferase 1. | -1,012 | 10,887 | -3,794 | 3,822E-03 | 0,039 |
| POLE2 | Homo sapiens polymerase (DNA directed) epsilon 2 (p59 subunit). | -1,017 | 8,064 | -9,093 | 5,200E-06 | 0,002 |
| PRSS23 | Homo sapiens protease serine 23. | -1,021 | 10,165 | -3,786 | 3,870E-03 | 0,039 |
| SUPT16H | Homo sapiens suppressor of Ty 16 homolog (S. cerevisiae). | -1,025 | 10,316 | -4,722 | 9,227E-04 | 0,018 |
| FOXM1 | Homo sapiens forkhead box M1, trans. var. 2. | -1,027 | 8,405 | -3,576 | 5,426E-03 | 0,048 |
| PNPO | Homo sapiens pyridoxamine 5'-phosphate oxidase. | -1,032 | 9,260 | -3,726 | 4,256E-03 | 0,042 |
| DEK | Homo sapiens DEK oncogene (DNA binding). | -1,041 | 9,832 | -4,028 | 2,639E-03 | 0,031 |
| MSH6 | Homo sapiens mutS homolog 6 (E. coli). | -1,041 | 9,752 | -5,496 | 3,094E-04 | 0,010 |
| THOC4 | PREDICTED: Homo sapiens THO complex 4. | -1,043 | 10,021 | -5,872 | 1,879E-04 | 0,008 |
| KIF11 | Homo sapiens kinesin family member 11. | -1,044 | 8,213 | -4,458 | 1,364E-03 | 0,022 |
| PTDSS1 | Homo sapiens phosphatidylserine synthase 1. | -1,045 | 9,876 | -4,256 | 1,855E-03 | 0,026 |
| SIVA1 | Homo sapiens SIVA1 apoptosis-inducing factor, trans. var. 2. | -1,052 | 10,808 | -4,871 | 7,428E-04 | 0,016 |
| MCM10 | Homo sapiens minichromosome maintenance complex component 10, trans. var. 2. | -1,061 | 8,085 | -7,435 | 2,859E-05 | 0,004 |
| GMNN | Homo sapiens geminin, DNA replication inhibitor. | -1,062 | 8,397 | -9,040 | 5,471E-06 | 0,002 |
| ATP1B1 | Homo sapiens ATPase. Na+/K+ transporting. β-1 polypeptide, trans. var. 1. | -1,065 | 9,539 | -4,689 | 9,682E-04 | 0,018 |
| RDH16 | Homo sapiens retinol dehydrogenase 16 (all-trans). | -1,065 | 7,665 | -4,078 | 2,442E-03 | 0,030 |
| RFC4 | Homo sapiens replication factor C (activator 1) 4, 37kDa, trans. var. 1. | -1,067 | 10,597 | -3,565 | 5,519E-03 | 0,049 |
| PYCARD | Homo sapiens PYD and CARD domain containing, trans. var. 1. | -1,070 | 9,809 | -4,295 | 1,748E-03 | 0,025 |
| EXO1 | Homo sapiens exonuclease 1, trans. var. 1. | -1,075 | 8,216 | -4,672 | 9,928E-04 | 0,018 |
| COQ3 | Homo sapiens coenzyme Q3 homolog methyltransferase (S. cerevisiae). | -1,076 | 8,698 | -4,675 | 9,885E-04 | 0,018 |
| RPA1 | Homo sapiens replication protein A1. 70kDa. | -1,077 | 10,418 | -4,454 | 1,374E-03 | 0,022 |
| STRA13 | Homo sapiens stimulated by retinoic acid 13 homolog (mouse). | -1,083 | 10,043 | -7,523 | 2,595E-05 | 0,004 |
| SKA2 | Homo sapiens spindle and kinetochore associated complex subunit 2 trans. var. 1. | -1,085 | 9,351 | -5,366 | 3,696E-04 | 0,011 |
| TNS3 | Homo sapiens tensin 3. | -1,089 | 9,174 | -4,812 | 8,091E-04 | 0,017 |
| CPA4 | Homo sapiens carboxypeptidase A4. | -1,090 | 7,777 | -6,420 | 9,381E-05 | 0,006 |
| ZWINT | Homo sapiens ZW10 interactor, trans. var. 3. | -1,091 | 8,097 | -7,254 | 3,503E-05 | 0,004 |
| TIMELESS | Homo sapiens timeless homolog (Drosophila). | -1,094 | 8,743 | -4,066 | 2,487E-03 | 0,030 |
| MGC40489 | PREDICTED: Homo sapiens hypothetical protein MGC40489, misc RNA. | -1,096 | 9,533 | -4,188 | 2,058E-03 | 0,027 |
| RAD51C | Homo sapiens RAD51 homolog C (S. cerevisiae), trans. var. 2 | -1,108 | 8,613 | -4,801 | 8,223E-04 | 0,017 |
| NUSAP1 | Homo sapiens nucleolar and spindle associated protein 1, trans. var. 2. | -1,110 | 8,356 | -3,826 | 3,630E-03 | 0,038 |
| PTPLB | Homo sapiens protein tyrosine phosphatase-like (proline instead of catalytic arginine), member b. | -1,121 | 10,121 | -3,639 | 4,900E-03 | 0,045 |
| ARHGAP23 | PREDICTED: Homo sapiens Rho GTPase activating protein 23, trans. var. 1. | -1,122 | 8,358 | -5,672 | 2,445E-04 | 0,009 |
| KPNA2 | Homo sapiens karyopherin α-2 (RAG cohort 1. importin alpha 1). | -1,122 | 9,457 | -3,890 | 3,282E-03 | 0,036 |
| MYLIP | Homo sapiens myosin regulatory light chain interacting protein. | -1,127 | 7,932 | -6,749 | 6,297E-05 | 0,005 |
| RRM2 | Homo sapiens ribonucleotide reductase M2 polypeptide. | -1,130 | 8,537 | -4,132 | 2,247E-03 | 0,029 |
| CCDC34 | Homo sapiens coiled-coil domain containing 34, trans. var. 1 | -1,132 | 8,356 | -3,626 | 5,000E-03 | 0,046 |
| PTPLB | Homo sapiens protein tyrosine phosphatase-like (proline instead of catalytic arginine), member b. | -1,135 | 9,799 | -4,210 | 1,990E-03 | 0,027 |
| RPA1 | Homo sapiens replication protein A1, 70kDa. | -1,136 | 9,222 | -6,361 | 1,009E-04 | 0,006 |
| SNHG3-RCC1 | Homo sapiens SNHG3-RCC1 readthrough transcript, trans. var. 1. | -1,137 | 9,188 | -7,226 | 3,614E-05 | 0,004 |
| STMN1 | Homo sapiens stathmin 1, trans. var. 1. | -1,138 | 9,341 | -3,668 | 4,677E-03 | 0,044 |
| DUT | Homo sapiens deoxyuridine triphosphatase, nuclear gene encoding mitochondrial protein, trans. var. 1. | -1,147 | 8,167 | -4,322 | 1,678E-03 | 0,024 |
| RAD51AP1 | Homo sapiens RAD51 associated protein 1. | -1,156 | 8,446 | -4,012 | 2,708E-03 | 0,032 |
| PBK | Homo sapiens PDZ binding kinase. | -1,161 | 8,486 | -3,544 | 5,709E-03 | 0,050 |
| MCM5 | Homo sapiens minichromosome maintenance complex component 5 (MCM5) | -1,169 | 8,885 | -4,606 | 1,094E-03 | 0,019 |
| C16orf33 | Homo sapiens chromosome 16 open reading frame 33. | -1,177 | 9,175 | -5,820 | 2,010E-04 | 0,008 |
| E2F2 | Homo sapiens E2F transcription factor 2. | -1,180 | 7,715 | -13,974 | 1,122E-07 | 0,001 |
| HADH | Homo sapiens hydroxyacyl-Coenzyme A dehydrogenase, nuclear gene encoding mitochondrial protein. | -1,184 | 8,553 | -3,920 | 3,129E-03 | 0,035 |
| FAM111A | Homo sapiens family with sequence similarity 111, member A, trans. var. 1. | -1,192 | 9,010 | -5,401 | 3,522E-04 | 0,011 |
| CCNF | Homo sapiens cyclin F. | -1,196 | 8,792 | -3,810 | 3,723E-03 | 0,038 |
| LOC728873 | PREDICTED: Homo sapiens misc_RNA, miscRNA. | -1,199 | 11,085 | -3,788 | 3,856E-03 | 0,039 |
| DPYSL3 | Homo sapiens dihydropyrimidinase-like 3. | -1,200 | 9,288 | -3,759 | 4,040E-03 | 0,040 |
| HMGB2 | Homo sapiens high-mobility group box 2. | -1,210 | 8,725 | -3,885 | 3,305E-03 | 0,036 |
| MCM4 | Homo sapiens minichromosome maintenance complex component 4, trans. var. 2. | -1,214 | 8,696 | -7,316 | 3,265E-05 | 0,004 |
| WNT10A | Homo sapiens wingless-type MMTV integration site family, member 10A. | -1,214 | 7,860 | -5,059 | 5,671E-04 | 0,014 |
| ARHGDIB | Homo sapiens Rho GDP dissociation inhibitor (GDI) β. | -1,227 | 8,231 | -5,370 | 3,676E-04 | 0,011 |
| LYPD1 | Homo sapiens LY6/PLAUR domain containing 1, trans. var. 1. | -1,232 | 7,873 | -7,503 | 2,651E-05 | 0,004 |
| RHOBTB3 | Homo sapiens Rho-related BTB domain containing 3. | -1,246 | 8,478 | -4,688 | 9,690E-04 | 0,018 |
| MCM7 | Homo sapiens minichromosome maintenance complex component 7, trans. var. 2. | -1,261 | 11,811 | -4,181 | 2,083E-03 | 0,027 |
| SNCA | Homo sapiens synuclein α (non A4 component of amyloid precursor) trans. var. NACP112. | -1,280 | 9,072 | -4,290 | 1,760E-03 | 0,025 |
| DLGAP5 | Homo sapiens discs. large (Drosophila) homolog-associated protein 5. | -1,307 | 8,535 | -3,707 | 4,388E-03 | 0,042 |
| MCM4 | Homo sapiens minichromosome maintenance complex component 4 trans. var. 1. | -1,336 | 10,244 | -5,299 | 4,053E-04 | 0,012 |
| FEN1 | Homo sapiens flap structure-specific endonuclease 1. | -1,344 | 9,977 | -7,435 | 2,859E-05 | 0,004 |
| CXXC5 | Homo sapiens CXXC finger 5. | -1,345 | 8,947 | -3,871 | 3,380E-03 | 0,036 |
| ID3 | Homo sapiens inhibitor of DNA binding 3, dominant negative helix-loop-helix protein. | -1,346 | 7,882 | -5,430 | 3,384E-04 | 0,011 |
| SOX2 | Homo sapiens SRY (sex determining region Y)-box 2. | -1,347 | 7,790 | -13,773 | 1,281E-07 | 0,001 |
| UBE2T | Homo sapiens ubiquitin-conjugating enzyme E2T (putative). | -1,382 | 9,210 | -4,217 | 1,969E-03 | 0,026 |
| C18orf56 | Homo sapiens chromosome 18 open reading frame 56. | -1,388 | 8,683 | -4,366 | 1,567E-03 | 0,024 |
| LOC100134073 | PREDICTED: Homo sapiens similar to LYPDC1 protein. | -1,398 | 7,936 | -6,744 | 6,333E-05 | 0,005 |
| FBXO5 | Homo sapiens F-box protein 5. | -1,401 | 8,629 | -6,458 | 8,949E-05 | 0,006 |
| PRIM1 | Homo sapiens primase, DNA, polypeptide 1 (49kDa). | -1,402 | 8,594 | -5,854 | 1,923E-04 | 0,008 |
| CENPM | Homo sapiens centromere protein M, trans. var. 2. | -1,402 | 8,364 | -5,438 | 3,347E-04 | 0,011 |
| PRIM1 | Homo sapiens primase, DNA, polypeptide 1 (49kDa). | -1,406 | 8,580 | -5,814 | 2,026E-04 | 0,008 |
| DLL1 | Homo sapiens delta-like 1 (Drosophila). | -1,422 | 9,640 | -5,914 | 1,779E-04 | 0,008 |
| SPC24 | Homo sapiens SPC24, NDC80 kinetochore complex component, homolog (S. cerevisiae). | -1,456 | 8,601 | -5,487 | 3,132E-04 | 0,010 |
| PCNA | Homo sapiens proliferating cell nuclear antigen, trans. var. 2. | -1,464 | 9,932 | -5,254 | 4,316E-04 | 0,012 |
| UHRF1 | Homo sapiens ubiquitin-like with PHD and ring finger domains 1, trans. var. 1. | -1,469 | 9,288 | -5,981 | 1,631E-04 | 0,008 |
| RFC5 | Homo sapiens replication factor C (activator 1) 5, 36.5kDa, trans. var. 1. | -1,470 | 9,215 | -4,070 | 2,472E-03 | 0,030 |
| NUSAP1 | Homo sapiens nucleolar and spindle associated protein 1, trans. var. 2. | -1,470 | 8,864 | -3,540 | 5,750E-03 | 0,050 |
| SOX2 | Homo sapiens SRY (sex determining region Y)-box 2. | -1,505 | 7,906 | -13,695 | 1,349E-07 | 0,001 |
| OIP5 | Homo sapiens Opa interacting protein 5. | -1,508 | 8,896 | -3,814 | 3,703E-03 | 0,038 |
| HSPE1 | Homo sapiens heat shock 10kDa protein 1 (chaperonin 10). | -1,515 | 9,314 | -4,003 | 2,744E-03 | 0,032 |
| ASF1B | Homo sapiens ASF1 anti-silencing function 1 homolog B (S. cerevisiae). | -1,563 | 8,549 | -5,537 | 2,930E-04 | 0,010 |
| MCM6 | Homo sapiens minichromosome maintenance complex component 6 | -1,564 | 10,055 | -4,797 | 8,268E-04 | 0,017 |
| CXXC5 | Homo sapiens CXXC finger 5. | -1,571 | 9,239 | -3,836 | 3,575E-03 | 0,037 |
| VSNL1 | Homo sapiens visinin-like 1. | -1,591 | 8,498 | -4,802 | 8,206E-04 | 0,017 |
| MCM2 | Homo sapiens minichromosome maintenance complex component 2. | -1,616 | 8,834 | -6,183 | 1,261E-04 | 0,007 |
| CDCA7 | Homo sapiens cell division cycle associated 7, trans. var. 1. | -1,619 | 8,873 | -4,565 | 1,164E-03 | 0,020 |
| BIRC5 | Homo sapiens baculoviral IAP repeat-containing 5, transcript variant 1. | -1,628 | 8,922 | -3,871 | 3,378E-03 | 0,036 |
| MCM3 | Homo sapiens minichromosome maintenance complex component 3. | -1,630 | 11,671 | -4,777 | 8,515E-04 | 0,017 |
| SKP2 | Homo sapiens S-phase kinase-associated protein 2 (p45), trans. var. 2. | -1,632 | 9,342 | -5,321 | 3,933E-04 | 0,011 |
| LOC731314 | PREDICTED: Homo sapiens similar to H2A histone family, member X. | -1,676 | 9,787 | -5,813 | 2,029E-04 | 0,008 |
| H2AFX | Homo sapiens H2A histone family, member X. | -1,714 | 8,952 | -7,455 | 2,798E-05 | 0,004 |
| UNG | Homo sapiens uracil-DNA glycosylase, nuclear gene encoding mitochondrial protein, trans. var. 1. | -1,721 | 9,962 | -3,852 | 3,484E-03 | 0,037 |
| KIAA0101 | Homo sapiens KIAA0101, trans. var. 1. | -1,774 | 9,433 | -3,677 | 4,610E-03 | 0,044 |
| KIF20A | Homo sapiens kinesin family member 20A. | -1,805 | 8,721 | -4,033 | 2,618E-03 | 0,031 |
| LFNG | Homo sapiens LFNG O-fucosylpeptide 3-β-N-acetylglucosaminyltransferase, trans. var. 1. | -1,808 | 8,313 | -3,561 | 5,559E-03 | 0,049 |
| GINS2 | Homo sapiens GINS complex subunit 2 (Psf2 homolog). | -1,815 | 9,505 | -4,614 | 1,081E-03 | 0,019 |
| FAM83D | Homo sapiens family with sequence similarity 83, member D. | -1,854 | 9,120 | -4,123 | 2,276E-03 | 0,029 |
| MCM3 | Homo sapiens minichromosome maintenance complex component 3. | -1,865 | 9,837 | -6,315 | 1,068E-04 | 0,006 |
| TYMS | Homo sapiens thymidylate synthetase. | -1,934 | 10,235 | -3,936 | 3,049E-03 | 0,034 |
