# Supplementary material for: Upregulation of cell cycle genes in head and neck cancer patients may be antagonized by erufosine’s down regulation of cell cycle processes in OSCC cells
Source: Oncotarget. 2017 Dec 20;9(5):5797–810. doi: 10.18632/oncotarget.23537 (PMC5814175; doi:10.18632/oncotarget.23537)
Supplement: Supplementary file 3 [file oncotarget-09-5797-s003.docx]

**Table 3C: List of differentially regulated genes in HN-5 cells (IC75 vs Control)**

| **Symbol** | **Definition** | **Log FC** | **Avg. Expression** | **t-statistics** | **p value** | **Adj. p-value** |
| --- | --- | --- | --- | --- | --- | --- |
| RN5S9 | Homo sapiens RNA, 5S ribosomal 9, ribosomal RNA. | 4,389 | 9,316 | 5,667 | 0,000 | 0,004 |
| CSF2 | Homo sapiens colony stimulating factor 2 (granulocyte-macrophage). | 4,170 | 9,656 | 9,913 | 0,000 | 0,000 |
| HBEGF | Homo sapiens heparin-binding EGF-like growth factor | 4,165 | 10,497 | 9,595 | 0,000 | 0,001 |
| KLF6 | Homo sapiens Kruppel-like factor 6, trans. var. 2. | 4,133 | 11,162 | 13,075 | 0,000 | 0,000 |
| KLF6 | Homo sapiens Kruppel-like factor 6, trans. var. 1. | 4,120 | 10,386 | 12,247 | 0,000 | 0,000 |
| LOC100008589 | Homo sapiens 28S ribosomal RNA, non-coding RNA. | 4,027 | 11,080 | 8,928 | 0,000 | 0,001 |
| RN7SK | Homo sapiens RNA, 7SK small nuclear, non-coding RNA. | 3,982 | 9,085 | 6,486 | 0,000 | 0,002 |
| CDKN1A | Homo sapiens cyclin-dependent kinase inhibitor 1A (p21. Cip1), trans. var. 1. | 3,852 | 11,510 | 10,382 | 0,000 | 0,000 |
| LOC100132564 | PREDICTED: Homo sapiens hypothetical protein LOC100132564. | 3,795 | 11,239 | 9,522 | 0,000 | 0,001 |
| LOC100134364 | PREDICTED: Homo sapiens hypothetical protein LOC100134364. | 3,755 | 10,937 | 9,979 | 0,000 | 0,000 |
| SNORD3D | Homo sapiens small nucleolar RNA, C/D box 3D, small nucleolar RNA. | 3,621 | 8,938 | 5,992 | 0,000 | 0,003 |
| RN7SK | Homo sapiens RNA, 7SK small nuclear, non-coding RNA. | 3,483 | 8,485 | 5,794 | 0,000 | 0,003 |
| LOC100132394 | PREDICTED: Homo sapiens hypothetical protein LOC100132394. | 3,460 | 11,375 | 9,583 | 0,000 | 0,001 |
| LOC650517 | PREDICTED: Homo sapiens hypothetical LOC650517. | 3,437 | 10,736 | 6,663 | 0,000 | 0,002 |
| MMP10 | Homo sapiens matrix metallopeptidase 10 (stromelysin 2). | 3,400 | 10,764 | 6,731 | 0,000 | 0,002 |
| TRIB1 | Homo sapiens tribbles homolog 1 (Drosophila). | 3,351 | 10,400 | 8,358 | 0,000 | 0,001 |
| ANGPTL4 | Homo sapiens angiopoietin-like 4, trans. var. 1. | 3,328 | 9,836 | 8,071 | 0,000 | 0,001 |
| RHOB | Homo sapiens ras homolog gene family, member B. | 3,270 | 10,151 | 11,730 | 0,000 | 0,000 |
| LOC100008588 | Homo sapiens 18S ribosomal RNA, non-coding RNA. | 3,228 | 11,305 | 8,854 | 0,000 | 0,001 |
| KLF2 | Homo sapiens Kruppel-like factor 2 (lung). | 3,209 | 9,619 | 14,476 | 0,000 | 0,000 |
| ODC1 | Homo sapiens ornithine decarboxylase 1. | 3,207 | 11,914 | 9,485 | 0,000 | 0,001 |
| DUSP1 | Homo sapiens dual specificity phosphatase 1. | 3,093 | 9,044 | 13,551 | 0,000 | 0,000 |
| PLAUR | Homo sapiens plasminogen activator, urokinase receptor, trans. var. 2. | 3,076 | 9,575 | 9,314 | 0,000 | 0,001 |
| MMP1 | Homo sapiens matrix metallopeptidase 1 (interstitial collagenase). | 3,063 | 9,288 | 3,352 | 0,008 | 0,034 |
| PPP1R15A | Homo sapiens protein phosphatase 1, regulatory (inhibitor) subunit 15A. | 3,032 | 10,980 | 9,966 | 0,000 | 0,000 |
| DUSP5 | Homo sapiens dual specificity phosphatase 5. | 3,030 | 10,463 | 13,292 | 0,000 | 0,000 |
| IL8 | Homo sapiens interleukin 8. | 3,003 | 8,672 | 15,457 | 0,000 | 0,000 |
| RNU1A3 | Homo sapiens RNA, U1A3 small nuclear, small nuclear RNA. | 2,950 | 9,166 | 7,716 | 0,000 | 0,001 |
| SNORD3A | Homo sapiens small nucleolar RNA. C/D box 3A, small nucleolar RNA. | 2,791 | 8,505 | 6,049 | 0,000 | 0,003 |
| IL1B | Homo sapiens interleukin 1- β. | 2,788 | 11,040 | 3,700 | 0,004 | 0,022 |
| AKR1C4 | Homo sapiens aldo-keto reductase family 1, member C4 (chlordecone reduc-tase; 3-α-hydroxysteroid dehydrogenase, type I; dihydrodiol dehydrogenase 4). | 2,769 | 9,373 | 3,980 | 0,003 | 0,016 |
| ATF3 | Homo sapiens activating transcription factor 3, trans. var. 4. | 2,743 | 8,589 | 18,528 | 0,000 | 0,000 |
| TM4SF19 | Homo sapiens transmembrane 4 L six family member 19. | 2,727 | 10,466 | 6,196 | 0,000 | 0,002 |
| PLAUR | Homo sapiens plasminogen activator, urokinase receptor, trans. var. 1. | 2,710 | 9,191 | 8,965 | 0,000 | 0,001 |
| GRB7 | Homo sapiens growth factor receptor-bound protein 7, trans. var. 2. | 2,681 | 9,219 | 11,071 | 0,000 | 0,000 |
| PHLDA1 | Homo sapiens pleckstrin homology-like domain, family A. member 1. | 2,669 | 12,250 | 10,991 | 0,000 | 0,000 |
| RNU1F1 | Homo sapiens RNA, U1F1 small nuclear, small nuclear RNA. | 2,669 | 8,543 | 6,851 | 0,000 | 0,002 |
| ZFP36 | Homo sapiens zinc finger protein 36. C3H type, homolog (mouse). | 2,625 | 9,957 | 10,946 | 0,000 | 0,000 |
| RNU1-5 | Homo sapiens RNA, U1 small nuclear 5, small nuclear RNA. | 2,619 | 8,669 | 7,471 | 0,000 | 0,001 |
| LAMC2 | Homo sapiens laminin gamma 2, trans. var. 1. | 2,604 | 11,266 | 6,175 | 0,000 | 0,003 |
| RNU1-3 | Homo sapiens RNA, U1 small nuclear 3, small nuclear RNA. | 2,582 | 8,541 | 8,061 | 0,000 | 0,001 |
| BTG1 | Homo sapiens B-cell translocation gene 1, anti-proliferative. | 2,579 | 10,939 | 10,997 | 0,000 | 0,000 |
| IL11 | Homo sapiens interleukin 11. | 2,539 | 8,668 | 10,572 | 0,000 | 0,000 |
| KRT17P3 | PREDICTED: Homo sapiens misc_RNA, miscRNA. | 2,481 | 11,880 | 5,492 | 0,000 | 0,004 |
| SNORD3C | Homo sapiens small nucleolar RNA, C/D box 3C, small nucleolar RNA. | 2,460 | 8,149 | 4,407 | 0,001 | 0,011 |
| RNU1G2 | Homo sapiens RNA, U1G2 small nuclear, small nuclear RNA. | 2,445 | 8,463 | 7,600 | 0,000 | 0,001 |
| KRT16 | Homo sapiens keratin 16 (focal non-epidermolytic palmoplantar keratoderma). | 2,431 | 11,085 | 5,110 | 0,001 | 0,006 |
| GJB3 | Homo sapiens gap junction protein β-3, 31kDa, trans. var. 2. | 2,428 | 10,128 | 8,068 | 0,000 | 0,001 |
| KCNK1 | Homo sapiens potassium channel, subfamily K, member 1. | 2,400 | 9,743 | 6,890 | 0,000 | 0,002 |
| TRIB3 | Homo sapiens tribbles homolog 3 (Drosophila). | 2,373 | 9,958 | 6,348 | 0,000 | 0,002 |
| PIM1 | Homo sapiens pim-1 oncogene. | 2,365 | 9,120 | 10,934 | 0,000 | 0,000 |
| SPRY2 | Homo sapiens sprouty homolog 2 (Drosophila). | 2,360 | 9,110 | 4,593 | 0,001 | 0,009 |
| LAMC2 | Homo sapiens laminin gamma 2, trans. var. 1. | 2,349 | 11,919 | 5,162 | 0,000 | 0,005 |
| KCNK1 | Homo sapiens potassium channel, subfamily K, member 1. | 2,343 | 9,239 | 6,004 | 0,000 | 0,003 |
| AKAP12 | Homo sapiens A kinase (PRKA) anchor protein (gravin) 12, trans. var. 2. | 2,331 | 8,908 | 8,385 | 0,000 | 0,001 |
| ISG20 | Homo sapiens interferon stimulated exonuclease gene 20kDa. | 2,327 | 9,916 | 5,748 | 0,000 | 0,003 |
| C12orf35 | Homo sapiens chromosome 12 open reading frame 35. | 2,315 | 8,997 | 7,020 | 0,000 | 0,002 |
| SAT1 | Homo sapiens spermidine/spermine N1-acetyltransferase 1. | 2,303 | 11,071 | 8,657 | 0,000 | 0,001 |
| AKR1C2 | Homo sapiens aldo-keto reductase family 1, member C2 (dihydrodiol dehydro-genase 2; bile acid binding protein; 3-α-hydroxysteroid dehydrogenase, type III), trans. var. 1. | 2,299 | 9,809 | 4,247 | 0,002 | 0,012 |
| ULK1 | Homo sapiens unc-51-like kinase 1 (C. elegans). | 2,273 | 10,155 | 6,187 | 0,000 | 0,002 |
| NDRG1 | Homo sapiens N-myc downstream regulated gene 1. | 2,253 | 12,379 | 5,535 | 0,000 | 0,004 |
| ULK1 | Homo sapiens unc-51-like kinase 1 (C. elegans). | 2,248 | 10,446 | 6,207 | 0,000 | 0,002 |
| JUN | Homo sapiens jun oncogene. | 2,243 | 10,798 | 10,976 | 0,000 | 0,000 |
| MMP9 | Homo sapiens matrix metallopeptidase 9 (gelatinase B, 92kDa gelatinase. 92kDa type IV collagenase). | 2,242 | 8,841 | 5,934 | 0,000 | 0,003 |
| LAMC2 | Homo sapiens laminin, gamma 2, trans. var. 2. | 2,236 | 9,593 | 5,074 | 0,001 | 0,006 |
| ERRFI1 | Homo sapiens ERBB receptor feedback inhibitor 1. | 2,233 | 10,536 | 7,895 | 0,000 | 0,001 |
| ITGA2 | Homo sapiens integrin α-2 (CD49B, α-2 subunit of VLA-2 receptor). | 2,222 | 10,847 | 8,145 | 0,000 | 0,001 |
| TMBIM1 | Homo sapiens transmembrane BAX inhibitor motif containing 1. | 2,179 | 9,555 | 5,978 | 0,000 | 0,003 |
| TM4SF19 | PREDICTED: Homo sapiens transmembrane 4 L six family member 19, trans. var. 2. | 2,161 | 9,571 | 4,402 | 0,001 | 0,011 |
| RAP1GAP | Homo sapiens RAP1 GTPase activating protein. | 2,159 | 9,367 | 9,626 | 0,000 | 0,000 |
| LAMB3 | Homo sapiens laminin β-3, trans. var. 1. | 2,159 | 12,851 | 9,259 | 0,000 | 0,001 |
| TNFRSF25 | Homo sapiens tumor necrosis factor receptor superfamily, member 25, trans. var. 10. | 2,152 | 9,359 | 6,699 | 0,000 | 0,002 |
| PLAU | Homo sapiens plasminogen activator, urokinase. | 2,149 | 12,748 | 11,757 | 0,000 | 0,000 |
| EMP1 | Homo sapiens epithelial membrane protein 1. | 2,127 | 10,788 | 10,286 | 0,000 | 0,000 |
| MYADM | Homo sapiens myeloid-associated differentiation marker, trans. var. 4. | 2,119 | 9,420 | 14,218 | 0,000 | 0,000 |
| KRT17 | Homo sapiens keratin 17. | 2,113 | 12,238 | 5,650 | 0,000 | 0,004 |
| C1orf106 | Homo sapiens chromosome 1 open reading frame 106. | 2,104 | 9,543 | 8,017 | 0,000 | 0,001 |
| PRDM1 | Homo sapiens PR domain containing 1 with ZNF domain, trans. var. 1. | 2,102 | 8,258 | 3,940 | 0,003 | 0,017 |
| SERPINE1 | Homo sapiens serpin peptidase inhibitor, clade E (nexin, plasminogen activator inhibitor type 1), member 1. | 2,093 | 10,078 | 7,015 | 0,000 | 0,002 |
| RNU4-2 | Homo sapiens RNA, U4 small nuclear 2, small nuclear RNA. | 2,073 | 7,999 | 5,095 | 0,001 | 0,006 |
| RNF19B | Homo sapiens ring finger protein 19B. | 2,040 | 9,329 | 8,874 | 0,000 | 0,001 |
| MIR1974 | Homo sapiens microRNA 1974, microRNA. | 2,035 | 11,829 | 16,879 | 0,000 | 0,000 |
| C14orf78 | PREDICTED: Homo sapiens chromosome 14 open reading frame 78. | 2,031 | 9,358 | 6,528 | 0,000 | 0,002 |
| ARHGEF2 | Homo sapiens rho/rac guanine nucleotide exchange factor (GEF) 2. | 2,023 | 10,199 | 8,988 | 0,000 | 0,001 |
| MXD1 | Homo sapiens MAX dimerization protein 1. | 1,994 | 8,665 | 9,397 | 0,000 | 0,001 |
| IER3 | Homo sapiens immediate early response 3. | 1,981 | 12,800 | 6,276 | 0,000 | 0,002 |
| TSC22D1 | Homo sapiens TSC22 domain family, member 1, trans. var. 2. | 1,959 | 10,135 | 6,108 | 0,000 | 0,003 |
| SERPINB1 | Homo sapiens serpin peptidase inhibitor, clade B (ovalbumin), member 1. | 1,959 | 8,933 | 3,349 | 0,008 | 0,034 |
| IL8 | Homo sapiens interleukin 8. | 1,949 | 8,133 | 15,466 | 0,000 | 0,000 |
| ARID3B | Homo sapiens AT rich interactive domain 3B (BRIGHT-like). | 1,942 | 8,452 | 6,234 | 0,000 | 0,002 |
| LOC441763 | PREDICTED: Homo sapiens hypothetical LOC441763. | 1,932 | 9,419 | 5,638 | 0,000 | 0,004 |
| LOC100008589 | Homo sapiens 28S ribosomal RNA, non-coding RNA. | 1,928 | 12,951 | 8,750 | 0,000 | 0,001 |
| PLAUR | Homo sapiens plasminogen activator, urokinase receptor, trans. var. 2. | 1,924 | 8,653 | 5,866 | 0,000 | 0,003 |
| LOC100133565 | PREDICTED: Homo sapiens similar to hCG23738. | 1,922 | 9,616 | 5,601 | 0,000 | 0,004 |
| PMAIP1 | Homo sapiens phorbol-12-myristate-13-acetate-induced protein 1. | 1,909 | 8,584 | 5,553 | 0,000 | 0,004 |
| PHLDA1 | Homo sapiens pleckstrin homology-like domain, family A, member 1. | 1,905 | 8,780 | 5,141 | 0,001 | 0,005 |
| AHNAK2 | Homo sapiens AHNAK nucleoprotein 2. | 1,903 | 9,115 | 6,203 | 0,000 | 0,002 |
| STX1A | Homo sapiens syntaxin 1A (brain). | 1,902 | 8,663 | 7,795 | 0,000 | 0,001 |
| IRF6 | Homo sapiens interferon regulatory factor 6. | 1,892 | 10,250 | 8,962 | 0,000 | 0,001 |
| S100A6 | Homo sapiens S100 calcium binding protein A6. | 1,880 | 11,316 | 4,553 | 0,001 | 0,009 |
| HPCAL1 | Homo sapiens hippocalcin-like 1, trans. var. 2. | 1,849 | 10,708 | 8,821 | 0,000 | 0,001 |
| HIST2H2AA3 | Homo sapiens histone cluster 2, H2aa3. | 1,848 | 8,309 | 4,607 | 0,001 | 0,009 |
| CGB5 | Homo sapiens chorionic gonadotropin, β-polypeptide 5. | 1,848 | 8,909 | 5,595 | 0,000 | 0,004 |
| SMOX | Homo sapiens spermine oxidase, trans. var. 2. | 1,844 | 8,517 | 7,180 | 0,000 | 0,001 |
| CSNK1E | Homo sapiens casein kinase 1 epsilon, trans. var. 1. | 1,842 | 10,624 | 10,690 | 0,000 | 0,000 |
| IRAK2 | Homo sapiens interleukin-1 receptor-associated kinase 2. | 1,840 | 8,552 | 7,750 | 0,000 | 0,001 |
| CEBPB | Homo sapiens CCAAT/enhancer binding protein (C/EBP)-β. | 1,837 | 12,103 | 9,474 | 0,000 | 0,001 |
| TGFA | Homo sapiens transforming growth factor α (TGFA). | 1,834 | 9,581 | 6,026 | 0,000 | 0,003 |
| KLF9 | Homo sapiens Kruppel-like factor 9. | 1,829 | 8,544 | 6,133 | 0,000 | 0,003 |
| ECGF1 | Homo sapiens endothelial cell growth factor 1 (platelet-derived). | 1,826 | 10,409 | 9,723 | 0,000 | 0,000 |
| TMEM158 | Homo sapiens transmembrane protein 158. | 1,822 | 9,525 | 3,430 | 0,007 | 0,031 |
| RNU6-1 | Homo sapiens RNA, U6 small nuclear 1, small nuclear RNA. | 1,821 | 10,283 | 6,015 | 0,000 | 0,003 |
| KLF11 | PREDICTED: Homo sapiens Kruppel-like factor 11. | 1,808 | 8,866 | 7,304 | 0,000 | 0,001 |
| BCAR3 | Homo sapiens breast cancer anti-estrogen resistance 3. | 1,800 | 10,164 | 7,043 | 0,000 | 0,002 |
| GABARAPL1 | Homo sapiens GABA(A) receptor-associated protein like 1. | 1,779 | 9,364 | 6,434 | 0,000 | 0,002 |
| FOSL1 | Homo sapiens FOS-like antigen 1. | 1,774 | 10,368 | 7,609 | 0,000 | 0,001 |
| SPSB3 | Homo sapiens splA/ryanodine receptor domain and SOCS box containing 3. | 1,768 | 8,699 | 7,168 | 0,000 | 0,001 |
| RIOK3 | Homo sapiens RIO kinase 3 (yeast), trans. var. 1. | 1,768 | 9,804 | 7,318 | 0,000 | 0,001 |
| CAPRIN2 | Homo sapiens caprin family member 2, trans. var. 1. | 1,758 | 9,742 | 6,606 | 0,000 | 0,002 |
| TUBB2A | Homo sapiens tubulin β-2A. | 1,756 | 9,847 | 9,849 | 0,000 | 0,000 |
| HERPUD1 | Homo sapiens homocysteine-inducible, endoplasmic reticulum stress-inducible, ubiquitin-like domain member 1, trans. var. 3. | 1,747 | 9,744 | 9,533 | 0,000 | 0,001 |
| UPP1 | Homo sapiens uridine phosphorylase 1, trans. var. 1. | 1,746 | 11,053 | 10,248 | 0,000 | 0,000 |
| OAF | Homo sapiens OAF homolog (Drosophila). | 1,734 | 9,682 | 5,157 | 0,000 | 0,005 |
| WIPI1 | Homo sapiens WD repeat domain, phosphoinositide interacting 1. | 1,731 | 8,744 | 10,046 | 0,000 | 0,000 |
| NPC1 | Homo sapiens Niemann-Pick disease, type C1. | 1,729 | 9,255 | 9,431 | 0,000 | 0,001 |
| LOC441019 | PREDICTED: Homo sapiens hypothetical LOC441019. | 1,729 | 11,194 | 3,116 | 0,012 | 0,044 |
| TNFAIP3 | Homo sapiens tumor necrosis factor α-induced protein 3. | 1,726 | 8,502 | 8,498 | 0,000 | 0,001 |
| RNU6-15 | Homo sapiens RNA U6 small nuclear 15, small nuclear RNA. | 1,713 | 10,354 | 5,938 | 0,000 | 0,003 |
| TSC22D1 | Homo sapiens TSC22 domain family, member 1, trans. var. 2. | 1,710 | 11,099 | 6,029 | 0,000 | 0,003 |
| HERPUD1 | Homo sapiens homocysteine-inducible, endoplasmic reticulum stress-inducible, ubiquitin-like domain member 1, trans. var. 3. | 1,709 | 9,852 | 9,687 | 0,000 | 0,000 |
| C12orf35 | Homo sapiens chromosome 12 open reading frame 35. | 1,708 | 8,290 | 5,197 | 0,000 | 0,005 |
| LOC387841 | PREDICTED: Homo sapiens similar to ribosomal protein L13a, trans. var. 2. | 1,705 | 9,807 | 4,744 | 0,001 | 0,008 |
| TGFA | Homo sapiens transforming growth factor α, trans. var. 2. | 1,705 | 9,149 | 6,144 | 0,000 | 0,003 |
| ITGA5 | Homo sapiens integrin α-5 (fibronectin receptor. alpha polypeptide). | 1,701 | 8,861 | 4,892 | 0,001 | 0,007 |
| SLC38A2 | Homo sapiens solute carrier family 38, member 2. | 1,699 | 10,029 | 9,180 | 0,000 | 0,001 |
| LARP6 | Homo sapiens La ribonucleoprotein domain family, member 6, trans. var. 1. | 1,696 | 9,085 | 9,563 | 0,000 | 0,001 |
| LRRC8A | Homo sapiens leucine rich repeat containing 8 family member A. | 1,693 | 9,387 | 10,151 | 0,000 | 0,000 |
| KLC3 | Homo sapiens kinesin light chain 3, trans. var. 1. | 1,685 | 8,839 | 6,770 | 0,000 | 0,002 |
| IL1A | Homo sapiens interleukin 1 α. | 1,666 | 11,288 | 6,726 | 0,000 | 0,002 |
| FLNB | Homo sapiens filamin B β (actin binding protein 278). | 1,663 | 10,391 | 12,082 | 0,000 | 0,000 |
| CPXM1 | Homo sapiens carboxypeptidase X (M14 family), member 1. | 1,660 | 8,893 | 5,923 | 0,000 | 0,003 |
| MAGT1 | Homo sapiens magnesium transporter 1. | 1,654 | 9,333 | 8,431 | 0,000 | 0,001 |
| CD55 | Homo sapiens CD55 molecule, decay accelerating factor for complement (Cromer blood group). | 1,653 | 8,117 | 9,426 | 0,000 | 0,001 |
| PLEK2 | Homo sapiens pleckstrin 2. | 1,652 | 10,009 | 8,241 | 0,000 | 0,001 |
| LOC653506 | PREDICTED: Homo sapiens similar to meteorin, glial cell differentiation regulator-like. | 1,647 | 8,703 | 6,745 | 0,000 | 0,002 |
| NT5E | Homo sapiens 5'-nucleotidase, ecto (CD73). | 1,646 | 9,161 | 3,369 | 0,008 | 0,033 |
| NFKBIA | Homo sapiens nuclear factor of kappa light polypeptide gene enhancer in B-cells inhibitor α. | 1,645 | 10,813 | 9,165 | 0,000 | 0,001 |
| C5orf32 | Homo sapiens chromosome 5 open reading frame 32. | 1,642 | 9,239 | 6,220 | 0,000 | 0,002 |
| ANTXR2 | Homo sapiens anthrax toxin receptor 2. | 1,636 | 8,807 | 5,678 | 0,000 | 0,004 |
| SELS | Homo sapiens selenoprotein S, trans. var. 2. | 1,631 | 9,469 | 10,983 | 0,000 | 0,000 |
| C17orf91 | Homo sapiens chromosome 17 open reading frame 91, trans. var. 2. | 1,619 | 8,417 | 3,933 | 0,003 | 0,017 |
| NDEL1 | Homo sapiens nudE nuclear distribution gene E homolog (A. nidulans)-like 1, trans. var. 2. | 1,617 | 10,315 | 6,314 | 0,000 | 0,002 |
| FOXO3 | Homo sapiens forkhead box O3, trans. var. 2. | 1,615 | 10,228 | 8,587 | 0,000 | 0,001 |
| SMOX | Homo sapiens spermine oxidase, trans. var. 4. | 1,614 | 8,407 | 6,937 | 0,000 | 0,002 |
| LRRFIP1 | Homo sapiens leucine rich repeat (in FLII) interacting protein 1. | 1,605 | 10,181 | 7,959 | 0,000 | 0,001 |
| IRF9 | Homo sapiens interferon regulatory factor 9. | 1,603 | 8,952 | 5,203 | 0,000 | 0,005 |
| RAC2 | Homo sapiens ras-related C3 botulinum toxin substrate 2 (rho family. small GTP binding protein Rac2). | 1,594 | 11,657 | 6,061 | 0,000 | 0,003 |
| ITPR3 | Homo sapiens inositol 1.4.5-triphosphate receptor. type 3. | 1,589 | 10,616 | 4,829 | 0,001 | 0,007 |
| ABCC3 | Homo sapiens ATP-binding cassette, sub-family C (CFTR/MRP), member 3. | 1,587 | 9,183 | 6,088 | 0,000 | 0,003 |
| MCART1 | Homo sapiens mitochondrial carrier triple repeat 1. | 1,587 | 10,396 | 5,698 | 0,000 | 0,004 |
| DEDD2 | Homo sapiens death effector domain containing 2. | 1,584 | 9,854 | 9,629 | 0,000 | 0,000 |
| FAM84B | Homo sapiens family with sequence similarity 84, member B. | 1,581 | 10,189 | 7,096 | 0,000 | 0,001 |
| SELS | Homo sapiens selenoprotein S, trans. var. 2. | 1,580 | 10,875 | 10,485 | 0,000 | 0,000 |
| SERTAD1 | Homo sapiens SERTA domain containing 1. | 1,568 | 9,551 | 5,785 | 0,000 | 0,003 |
| BHLHB2 | Homo sapiens basic helix-loop-helix domain containing class B 2. | 1,566 | 11,006 | 5,585 | 0,000 | 0,004 |
| GRB7 | Homo sapiens growth factor receptor-bound protein 7, trans. var. 1. | 1,556 | 8,132 | 8,174 | 0,000 | 0,001 |
| CGB1 | Homo sapiens chorionic gonadotropin-β polypeptide 1. | 1,547 | 8,681 | 5,290 | 0,000 | 0,005 |
| CAPRIN2 | Homo sapiens caprin family member 2, trans. var. 2. | 1,542 | 8,868 | 5,870 | 0,000 | 0,003 |
| TICAM1 | Homo sapiens toll-like receptor adaptor molecule 1, trans. var. 2. | 1,541 | 8,792 | 6,218 | 0,000 | 0,002 |
| PLEC1 | Homo sapiens plectin 1, intermediate filament binding protein 500kDa, trans. var. 1. | 1,538 | 10,640 | 5,416 | 0,000 | 0,004 |
| LOC100130992 | PREDICTED: Homo sapiens similar to hCG2017625. | 1,537 | 7,842 | 5,550 | 0,000 | 0,004 |
| ISG15 | Homo sapiens ISG15 ubiquitin-like modifier. | 1,537 | 9,061 | 4,635 | 0,001 | 0,008 |
| IRS2 | Homo sapiens insulin receptor substrate 2. | 1,525 | 9,049 | 5,640 | 0,000 | 0,004 |
| VGF | Homo sapiens VGF nerve growth factor inducible. | 1,519 | 8,525 | 6,837 | 0,000 | 0,002 |
| MT2A | Homo sapiens metallothionein 2A. | 1,518 | 12,664 | 3,825 | 0,004 | 0,019 |
| TSC22D3 | Homo sapiens TSC22 domain family, member 3, trans. var. 1. | 1,518 | 9,224 | 6,874 | 0,000 | 0,002 |
| TMEM17 | Homo sapiens transmembrane protein 17. | 1,517 | 10,839 | 5,284 | 0,000 | 0,005 |
| CSNK1E | Homo sapiens casein kinase 1 epsilon, trans. var. 1. | 1,517 | 9,570 | 10,552 | 0,000 | 0,000 |
| PTAFR | Homo sapiens platelet-activating factor receptor (PTAFR) . | 1,517 | 8,338 | 3,148 | 0,011 | 0,042 |
| GNA15 | Homo sapiens guanine nucleotide binding protein (G protein) α-15 (Gq class). | 1,510 | 9,645 | 7,820 | 0,000 | 0,001 |
| PDLIM7 | Homo sapiens PDZ and LIM domain 7 (enigma), trans. var. 4. | 1,507 | 9,343 | 5,243 | 0,000 | 0,005 |
| KLF4 | Homo sapiens Kruppel-like factor 4 (gut). | 1,507 | 8,524 | 6,514 | 0,000 | 0,002 |
| HMOX1 | Homo sapiens heme oxygenase (decycling) 1. | 1,506 | 7,858 | 4,718 | 0,001 | 0,008 |
| CDCP1 | Homo sapiens CUB domain containing protein 1, trans. var. 1. | 1,505 | 9,314 | 6,107 | 0,000 | 0,003 |
| IL1RN | Homo sapiens interleukin 1 receptor antagonist, trans. var. 4. | 1,502 | 8,050 | 3,021 | 0,014 | 0,050 |
| EPAS1 | Homo sapiens endothelial PAS domain protein 1. | 1,491 | 9,724 | 6,314 | 0,000 | 0,002 |
| FKBP14 | Homo sapiens FK506 binding protein 14, 22 kDa. | 1,487 | 9,460 | 5,254 | 0,000 | 0,005 |
| SPIRE1 | Homo sapiens spire homolog 1 (Drosophila), trans. var. 2. | 1,486 | 10,431 | 8,331 | 0,000 | 0,001 |
| AHNAK | Homo sapiens AHNAK nucleoprotein, trans. var. 1. | 1,474 | 10,469 | 4,684 | 0,001 | 0,008 |
| OXSR1 | Homo sapiens oxidative-stress responsive 1. | 1,472 | 10,044 | 7,315 | 0,000 | 0,001 |
| SLC20A1 | Homo sapiens solute carrier family 20 (phosphate transporter), member 1. | 1,468 | 11,409 | 10,061 | 0,000 | 0,000 |
| SPAG9 | Homo sapiens sperm associated antigen 9. | 1,468 | 9,423 | 6,948 | 0,000 | 0,002 |
| YPEL5 | Homo sapiens yippee-like 5 (Drosophila). | 1,465 | 9,746 | 7,563 | 0,000 | 0,001 |
| LOC143666 | PREDICTED: Homo sapiens hypothetical protein LOC143666. | 1,464 | 8,247 | 9,826 | 0,000 | 0,000 |
| GSK3B | Homo sapiens glycogen synthase kinase 3-β. | 1,462 | 9,077 | 5,230 | 0,000 | 0,005 |
| SNORD12C | Homo sapiens small nucleolar RNA. C/D box 12C, small nucleolar RNA. | 1,461 | 8,134 | 4,981 | 0,001 | 0,006 |
| LRRC8C | Homo sapiens leucine rich repeat containing 8 family member C. | 1,461 | 8,820 | 5,138 | 0,001 | 0,005 |
| DMC1 | Homo sapiens DMC1 dosage suppressor of mck1 homolog, meiosis-specific homologous recombination (yeast). | 1,455 | 9,923 | 5,643 | 0,000 | 0,004 |
| ASNS | Homo sapiens asparagine synthetase, trans. var. 1. | 1,453 | 11,287 | 3,297 | 0,009 | 0,036 |
| ZFP36L1 | Homo sapiens zinc finger protein 36, C3H type-like 1. | 1,452 | 9,634 | 7,453 | 0,000 | 0,001 |
| LIMA1 | Homo sapiens LIM domain and actin binding 1. | 1,451 | 10,187 | 6,260 | 0,000 | 0,002 |
| KIAA1666 | PREDICTED: Homo sapiens KIAA1666 protein. | 1,448 | 7,698 | 4,362 | 0,002 | 0,011 |
| ZFP36L2 | Homo sapiens zinc finger protein 36, C3H type-like 2. | 1,447 | 8,887 | 4,950 | 0,001 | 0,006 |
| METRNL | PREDICTED: Homo sapiens meteorin, glial cell differentiation regulator-like. | 1,447 | 8,502 | 6,409 | 0,000 | 0,002 |
| CLIC4 | Homo sapiens chloride intracellular channel 4, nuclear gene encoding mitochondrial protein. | 1,446 | 9,564 | 8,238 | 0,000 | 0,001 |
| KIAA1949 | Homo sapiens KIAA1949. | 1,443 | 9,885 | 6,764 | 0,000 | 0,002 |
| LOC399900 | Homo sapiens hypothetical gene supported by AK093779. | 1,433 | 11,428 | 7,397 | 0,000 | 0,001 |
| OASL | Homo sapiens 2'-5'-oligoadenylate synthetase-like, trans. var. 2. | 1,433 | 8,048 | 3,279 | 0,009 | 0,036 |
| C20orf111 | Homo sapiens chromosome 20 open reading frame 111. | 1,430 | 10,526 | 7,611 | 0,000 | 0,001 |
| C10orf116 | Homo sapiens chromosome 10 open reading frame 116. | 1,427 | 9,679 | 3,342 | 0,008 | 0,034 |
| C8orf45 | Homo sapiens chromosome 8 open reading frame 45. | 1,418 | 10,092 | 6,162 | 0,000 | 0,003 |
| PNPT1 | Homo sapiens polyribonucleotide nucleotidyltransferase 1. | 1,418 | 10,864 | 5,942 | 0,000 | 0,003 |
| PTPN12 | Homo sapiens protein tyrosine phosphatase, non-receptor type 12. | 1,417 | 9,652 | 5,943 | 0,000 | 0,003 |
| PANX2 | Homo sapiens pannexin 2. | 1,416 | 8,835 | 4,096 | 0,002 | 0,014 |
| LOC728809 | PREDICTED: Homo sapiens hypothetical LOC728809. | 1,413 | 10,550 | 5,081 | 0,001 | 0,006 |
| CHIC2 | Homo sapiens cysteine-rich hydrophobic domain 2. | 1,410 | 9,640 | 7,025 | 0,000 | 0,002 |
| ATP9A | Homo sapiens ATPase, class II, type 9A. | 1,408 | 10,079 | 7,938 | 0,000 | 0,001 |
| CLK1 | Homo sapiens CDC-like kinase 1. | 1,405 | 9,201 | 4,956 | 0,001 | 0,006 |
| SH3PXD2A | Homo sapiens SH3 and PX domains 2A. | 1,396 | 10,304 | 4,217 | 0,002 | 0,013 |
| NLRP8 | Homo sapiens NLR family, pyrin domain containing 8. | 1,391 | 8,888 | 5,398 | 0,000 | 0,004 |
| LOC100133516 | PREDICTED: Homo sapiens hypothetical protein LOC10013351. | 1,389 | 8,661 | 7,420 | 0,000 | 0,001 |
| TMC6 | Homo sapiens transmembrane channel-like 6. | 1,389 | 8,474 | 6,037 | 0,000 | 0,003 |
| LOC255167 | Homo sapiens hypothetical LOC255167, non-coding RNA. | 1,388 | 9,362 | 4,999 | 0,001 | 0,006 |
| SPAG9 | Homo sapiens sperm associated antigen 9. | 1,387 | 9,168 | 5,405 | 0,000 | 0,004 |
| FLJ40504 | Homo sapiens hypothetical protein FLJ40504. | 1,381 | 9,533 | 4,653 | 0,001 | 0,008 |
| CD68 | Homo sapiens CD68 antigen. | 1,380 | 10,183 | 3,251 | 0,009 | 0,037 |
| CDCP1 | Homo sapiens CUB domain containing protein 1, trans. var. 2. | 1,380 | 8,288 | 4,554 | 0,001 | 0,009 |
| ARL16 | Homo sapiens ADP-ribosylation factor-like 16. | 1,374 | 10,336 | 5,833 | 0,000 | 0,003 |
| AMTN | Homo sapiens amelotin. | 1,374 | 8,409 | 6,080 | 0,000 | 0,003 |
| KLF13 | Homo sapiens Kruppel-like factor 13. | 1,371 | 9,205 | 5,896 | 0,000 | 0,003 |
| LOC100130168 | PREDICTED: Homo sapiens hypothetical protein LOC100130168. | 1,368 | 10,729 | 5,716 | 0,000 | 0,004 |
| SH3KBP1 | Homo sapiens SH3-domain kinase binding protein 1, trans. var. 1. | 1,368 | 9,837 | 6,224 | 0,000 | 0,002 |
| IL1RL1 | Homo sapiens interleukin 1 receptor-like 1, trans. var. 2. | 1,367 | 7,743 | 3,771 | 0,004 | 0,020 |
| BIRC3 | Homo sapiens baculoviral IAP repeat-containing 3, trans. var. 2. | 1,361 | 8,143 | 5,424 | 0,000 | 0,004 |
| ZBTB43 | Homo sapiens zinc finger and BTB domain containing 43. | 1,358 | 8,222 | 4,905 | 0,001 | 0,007 |
| ZNF682 | Homo sapiens zinc finger protein 682, trans. var. 1. | 1,356 | 9,469 | 4,739 | 0,001 | 0,008 |
| GFPT1 | Homo sapiens glutamine-fructose-6-phosphate transaminase 1. | 1,356 | 9,083 | 13,990 | 0,000 | 0,000 |
| S100A16 | Homo sapiens S100 calcium binding protein A16. | 1,355 | 11,239 | 5,418 | 0,000 | 0,004 |
| ANG | Homo sapiens angiogenin, ribonuclease, RNase A family 5, trans. var. 2. | 1,354 | 8,488 | 5,481 | 0,000 | 0,004 |
| TSC22D1 | Homo sapiens TSC22 domain family, member 1, trans. var. 1. | 1,350 | 8,458 | 4,462 | 0,001 | 0,010 |
| ASS1 | Homo sapiens argininosuccinate synthetase 1, trans. var. 1. | 1,348 | 8,700 | 5,054 | 0,001 | 0,006 |
| MMP3 | Homo sapiens matrix metallopeptidase 3 (stromelysin 1. progelatinase). | 1,348 | 7,662 | 3,781 | 0,004 | 0,020 |
| RNU4-1 | Homo sapiens RNA, U4 small nuclear 1, small nuclear RNA. | 1,347 | 7,642 | 3,660 | 0,005 | 0,023 |
| FAM129B | Homo sapiens family with sequence similarity 129, member B, trans. var. 1. | 1,347 | 11,628 | 5,450 | 0,000 | 0,004 |
| FOXO3 | Homo sapiens forkhead box O3, trans. var. 2. | 1,346 | 9,208 | 6,028 | 0,000 | 0,003 |
| CLCF1 | Homo sapiens cardiotrophin-like cytokine factor 1, trans. var. 1. | 1,346 | 8,470 | 5,853 | 0,000 | 0,003 |
| SNORD46 | Homo sapiens small nucleolar RNA, C/D box 46, small nucleolar RNA. | 1,345 | 7,733 | 4,689 | 0,001 | 0,008 |
| NP | Homo sapiens nucleoside phosphorylase. | 1,344 | 9,635 | 4,898 | 0,001 | 0,007 |
| NIPA1 | Homo sapiens non imprinted in Prader-Willi/Angelman syndrome 1. | 1,340 | 8,602 | 5,410 | 0,000 | 0,004 |
| HNRPDL | Homo sapiens heterogeneous nuclear ribonucleoprotein D-like, trans. var. 3, transcribed RNA. | 1,340 | 9,147 | 8,049 | 0,000 | 0,001 |
| SLC16A3 | Homo sapiens solute carrier family 16, member 3 (monocarboxylic acid transporter 4), trans. var. 2. | 1,340 | 8,747 | 6,733 | 0,000 | 0,002 |
| DUSP6 | Homo sapiens dual specificity phosphatase 6, trans. var. 2. | 1,339 | 9,857 | 4,050 | 0,003 | 0,015 |
| UGCG | Homo sapiens UDP-glucose ceramide glucosyltransferase. | 1,338 | 8,796 | 6,446 | 0,000 | 0,002 |
| RNU4ATAC | Homo sapiens RNA. U4atac small nuclear (U12-dependent splicing), small nuclear RNA. | 1,337 | 7,766 | 5,519 | 0,000 | 0,004 |
| ATF4 | Homo sapiens activating transcription factor 4 (tax-responsive enhancer element B67), trans. var. 2. | 1,336 | 9,158 | 7,117 | 0,000 | 0,001 |
| RIOK3 | Homo sapiens RIO kinase 3 (yeast). | 1,333 | 9,922 | 6,451 | 0,000 | 0,002 |
| LOC90586 | Homo sapiens AOC3 pseudogene, non-coding RNA. | 1,331 | 9,214 | 4,480 | 0,001 | 0,010 |
| MAP1LC3B | Homo sapiens microtubule-associated protein 1 light chain 3 β. | 1,329 | 9,021 | 9,570 | 0,000 | 0,001 |
| PPP2R2C | Homo sapiens protein phosphatase 2 (formerly 2A), regulatory subunit B, gamma isoform, trans. var. 2. | 1,328 | 8,718 | 5,361 | 0,000 | 0,005 |
| ZFAND2A | Homo sapiens zinc finger, AN1-type domain 2A. | 1,326 | 9,253 | 10,670 | 0,000 | 0,000 |
| POFUT1 | Homo sapiens protein O-fucosyltransferase 1, trans. var. 1. | 1,324 | 10,955 | 5,963 | 0,000 | 0,003 |
| LOC730313 | PREDICTED: Homo sapiens hypothetical LOC730313. | 1,324 | 10,968 | 5,420 | 0,000 | 0,004 |
| MCL1 | Homo sapiens myeloid cell leukemia sequence 1 (BCL2-related), trans. var. 1. | 1,323 | 9,721 | 7,168 | 0,000 | 0,001 |
| DNAJB2 | Homo sapiens DnaJ (Hsp40) homolog, subfamily B, member 2, trans. var. 2. | 1,322 | 10,656 | 5,283 | 0,000 | 0,005 |
| PRIC285 | Homo sapiens peroxisomal proliferator-activated receptor A interacting complex 285, trans. var. 2. | 1,321 | 8,443 | 7,802 | 0,000 | 0,001 |
| SPRR1A | Homo sapiens small proline-rich protein 1A. | 1,321 | 8,594 | 4,296 | 0,002 | 0,012 |
| FOXQ1 | Homo sapiens forkhead box Q1. | 1,321 | 9,682 | 3,583 | 0,005 | 0,025 |
| KRT16 | Homo sapiens keratin 16 (focal non-epidermolytic palmoplantar keratoderma). | 1,320 | 8,976 | 3,436 | 0,007 | 0,030 |
| LARP1B | Homo sapiens La ribonucleoprotein domain family, member 1B, trans. var. 3. | 1,320 | 9,128 | 10,310 | 0,000 | 0,000 |
| MALL | Homo sapiens mal. T-cell differentiation protein-like. | 1,320 | 10,350 | 4,286 | 0,002 | 0,012 |
| XRCC2 | Homo sapiens X-ray repair complementing defective repair in Chinese hamster cells 2. | 1,317 | 9,071 | 5,633 | 0,000 | 0,004 |
| SPHK1 | Homo sapiens sphingosine kinase 1, trans. var. 1. | 1,316 | 8,630 | 5,452 | 0,000 | 0,004 |
| ZNF486 | PREDICTED: Homo sapiens zinc finger protein 486. | 1,315 | 10,146 | 5,059 | 0,001 | 0,006 |
| OPLAH | Homo sapiens 5-oxoprolinase (ATP-hydrolysing). | 1,314 | 8,920 | 8,490 | 0,000 | 0,001 |
| GADD45A | Homo sapiens growth arrest and DNA-damage-inducible-α. | 1,313 | 9,613 | 5,654 | 0,000 | 0,004 |
| GADD45A | Homo sapiens growth arrest and DNA-damage-inducible-α. | 1,312 | 9,896 | 6,217 | 0,000 | 0,002 |
| KIAA1751 | Homo sapiens KIAA1751. | 1,307 | 9,048 | 5,619 | 0,000 | 0,004 |
| SDHALP1 | Homo sapiens succinate dehydrogenase complex, subunit A, flavoprotein pseudogene 1 on chromosome 3. | 1,307 | 8,465 | 5,050 | 0,001 | 0,006 |
| C8orf37 | Homo sapiens chromosome 8 open reading frame 37. | 1,307 | 10,511 | 6,543 | 0,000 | 0,002 |
| TGFB1I1 | Homo sapiens transforming growth factor β1 induced transcript 1, trans. var. 2. | 1,303 | 8,346 | 8,841 | 0,000 | 0,001 |
| SHROOM4 | Homo sapiens shroom family member 4. | 1,302 | 8,962 | 6,517 | 0,000 | 0,002 |
| RHOC | Homo sapiens ras homolog gene family, member C, trans. var. 2. | 1,302 | 10,122 | 5,320 | 0,000 | 0,005 |
| KIAA0913 | Homo sapiens KIAA0913. | 1,300 | 8,750 | 5,030 | 0,001 | 0,006 |
| SNORA80 | Homo sapiens small nucleolar RNA. H/ACA box 80, small nucleolar RNA. | 1,299 | 7,829 | 3,871 | 0,003 | 0,018 |
| PDE4C | Homo sapiens phosphodiesterase 4C, cAMP-specific (phosphodiesterase E1 dunce homolog. Drosophila). | 1,297 | 11,281 | 4,344 | 0,002 | 0,011 |
| DUSP10 | Homo sapiens dual specificity phosphatase 10, trans. var. 3. | 1,295 | 8,316 | 7,074 | 0,000 | 0,001 |
| C9orf80 | Homo sapiens chromosome 9 open reading frame 80. | 1,288 | 10,156 | 5,370 | 0,000 | 0,005 |
| PTGS2 | Homo sapiens prostaglandin-endoperoxide synthase 2 (prostaglandin G/H synthase and cyclooxygenase). | 1,288 | 7,972 | 3,279 | 0,009 | 0,036 |
| ZNF165 | Homo sapiens zinc finger protein 165. | 1,287 | 8,609 | 6,887 | 0,000 | 0,002 |
| ZNF483 | Homo sapiens zinc finger protein 483, trans. var. 2. | 1,287 | 8,546 | 5,127 | 0,001 | 0,006 |
| TIPARP | Homo sapiens TCDD-inducible poly(ADP-ribose) polymerase. | 1,287 | 9,978 | 4,058 | 0,003 | 0,015 |
| LOC401098 | PREDICTED: Homo sapiens misc_RNA, miscRNA. | 1,286 | 8,409 | 5,550 | 0,000 | 0,004 |
| HIST2H2AA4 | Homo sapiens histone cluster 2. H2aa4. | 1,278 | 7,958 | 3,811 | 0,004 | 0,020 |
| LOC645452 | PREDICTED: Homo sapiens similar to hCG1782414. | 1,278 | 10,500 | 4,809 | 0,001 | 0,007 |
| KIAA1539 | Homo sapiens KIAA1539. | 1,277 | 8,363 | 6,130 | 0,000 | 0,003 |
| ID2 | Homo sapiens inhibitor of DNA binding 2, dominant negative helix-loop-helix protein. | 1,276 | 7,688 | 5,467 | 0,000 | 0,004 |
| UBAP1 | Homo sapiens ubiquitin associated protein 1. | 1,269 | 10,178 | 8,912 | 0,000 | 0,001 |
| ARFGAP3 | Homo sapiens ADP-ribosylation factor GTPase activating protein 3. | 1,268 | 9,222 | 9,671 | 0,000 | 0,000 |
| LOC645452 | PREDICTED: Homo sapiens similar to hCG1782414. | 1,268 | 9,341 | 4,878 | 0,001 | 0,007 |
| PPA2 | Homo sapiens pyrophosphatase (inorganic) 2, nuclear gene encoding mitochondrial protein, trans. var. 2. | 1,267 | 9,588 | 4,829 | 0,001 | 0,007 |
| EIF1 | Homo sapiens eukaryotic translation initiation factor 1. | 1,266 | 12,149 | 10,667 | 0,000 | 0,000 |
| C3orf52 | Homo sapiens chromosome 3 open reading frame 52. | 1,266 | 8,466 | 5,626 | 0,000 | 0,004 |
| KIAA0363 | PREDICTED: Homo sapiens KIAA0363 protein. | 1,265 | 8,031 | 9,869 | 0,000 | 0,000 |
| LOC644132 | PREDICTED: Homo sapiens misc_RNA, miscRNA. | 1,263 | 8,832 | 6,516 | 0,000 | 0,002 |
| MOBKL2C | Homo sapiens MOB1, Mps One Binder kinase activator-like 2C (yeast), trans. var. 1. | 1,262 | 8,804 | 4,728 | 0,001 | 0,008 |
| YOD1 | Homo sapiens YOD1 OTU deubiquinating enzyme 1 homolog (S. cerevisiae). | 1,259 | 8,854 | 4,244 | 0,002 | 0,012 |
| ASNS | Homo sapiens asparagine synthetase, trans. var. 1. | 1,258 | 9,480 | 3,609 | 0,005 | 0,025 |
| RHOC | Homo sapiens ras homolog gene family, member C, trans. var. 1. | 1,258 | 11,856 | 7,930 | 0,000 | 0,001 |
| C13orf15 | Homo sapiens chromosome 13 open reading frame 15. | 1,257 | 8,564 | 6,960 | 0,000 | 0,002 |
| DUXAP3 | Homo sapiens double homeobox A pseudogene 3 on chromosome 10. | 1,254 | 10,169 | 4,754 | 0,001 | 0,008 |
| PTPN12 | Homo sapiens protein tyrosine phosphatase, non-receptor type 12. | 1,254 | 9,535 | 5,794 | 0,000 | 0,003 |
| PRKCD | Homo sapiens protein kinase C delta, trans. var. 1. | 1,253 | 8,956 | 7,385 | 0,000 | 0,001 |
| KCNK6 | Homo sapiens potassium channel, subfamily K, member 6. | 1,251 | 8,869 | 9,700 | 0,000 | 0,000 |
| ALPP | Homo sapiens alkaline phosphatase, placental (Regan isozyme). | 1,251 | 9,511 | 5,105 | 0,001 | 0,006 |
| ZNF430 | Homo sapiens zinc finger protein 430. | 1,250 | 11,202 | 5,623 | 0,000 | 0,004 |
| KRT18P13 | PREDICTED: Homo sapiens keratin 18 pseudogene 13. | 1,248 | 8,910 | 5,671 | 0,000 | 0,004 |
| AKAP12 | Homo sapiens A kinase (PRKA) anchor protein (gravin) 12, trans. var. 1. | 1,248 | 8,036 | 5,550 | 0,000 | 0,004 |
| ATP2B4 | Homo sapiens ATPase, Ca++ transporting, plasma membrane 4, trans. var. 2. | 1,244 | 8,751 | 6,666 | 0,000 | 0,002 |
| ZNF14 | Homo sapiens zinc finger protein 14. | 1,244 | 10,390 | 5,799 | 0,000 | 0,003 |
| LOC100132391 | PREDICTED: Homo sapiens hypothetical protein LOC100132391. | 1,243 | 10,006 | 5,991 | 0,000 | 0,003 |
| LOC389517 | Homo sapiens Williams Beuren syndrome chromosome region 19 pseudogene on chromosome 7. | 1,242 | 11,129 | 4,588 | 0,001 | 0,009 |
| ZNF549 | Homo sapiens zinc finger protein 549. | 1,242 | 11,036 | 5,838 | 0,000 | 0,003 |
| JUND | Homo sapiens jun D proto-oncogene. | 1,241 | 12,262 | 7,040 | 0,000 | 0,002 |
| LOC646463 | PREDICTED: Homo sapiens similar to Ubiquitin-conjugating enzyme E2 H (Ubiquitin-protein ligase H) (Ubiquitin carrier protein H) (UBCH2) (E2-20K). | 1,240 | 8,566 | 7,045 | 0,000 | 0,002 |
| CITED4 | Homo sapiens Cbp/p300-interacting transactivator with Glu/Asp-rich carboxy-terminal domain 4. | 1,239 | 9,997 | 6,404 | 0,000 | 0,002 |
| LRRC37B2 | Homo sapiens leucine rich repeat containing 37, member B2, non-coding RNA. | 1,239 | 9,424 | 5,176 | 0,000 | 0,005 |
| ZNF69 | Homo sapiens zinc finger protein 69. | 1,238 | 9,842 | 6,211 | 0,000 | 0,002 |
| CD68 | Homo sapiens CD68 molecule, trans. var. 1. | 1,236 | 9,292 | 4,271 | 0,002 | 0,012 |
| XBP1 | Homo sapiens X-box binding protein 1, trans. var. 1. | 1,235 | 10,855 | 7,889 | 0,000 | 0,001 |
| C14orf153 | Homo sapiens chromosome 14 open reading frame 153. | 1,232 | 9,792 | 6,536 | 0,000 | 0,002 |
| CLDN12 | Homo sapiens claudin 12. | 1,232 | 8,995 | 4,442 | 0,001 | 0,010 |
| LOC100128288 | Homo sapiens hypothetical protein LOC100128288, non-coding RNA. | 1,231 | 9,877 | 5,171 | 0,000 | 0,005 |
| DENND2C | Homo sapiens DENN/MADD domain containing 2C. | 1,231 | 8,348 | 4,420 | 0,001 | 0,010 |
| ZNF394 | Homo sapiens zinc finger protein 394. | 1,229 | 10,358 | 6,502 | 0,000 | 0,002 |
| LMOD3 | Homo sapiens leiomodin 3 (fetal). | 1,228 | 8,583 | 5,678 | 0,000 | 0,004 |
| TSC22D3 | Homo sapiens TSC22 domain family, member 3, trans. var. 2. | 1,227 | 8,441 | 7,464 | 0,000 | 0,001 |
| TNIP1 | Homo sapiens TNFAIP3 interacting protein 1. | 1,227 | 9,269 | 5,014 | 0,001 | 0,006 |
| EFNA1 | Homo sapiens ephrin-A1, trans. var. 1. | 1,225 | 9,478 | 5,300 | 0,000 | 0,005 |
| SPRY4 | Homo sapiens sprouty homolog 4 (Drosophila). | 1,224 | 8,234 | 4,609 | 0,001 | 0,009 |
| RALGDS | Homo sapiens ral guanine nucleotide dissociation stimulator, trans. var. 1. | 1,223 | 9,570 | 6,116 | 0,000 | 0,003 |
| GPSM1 | PREDICTED: Homo sapiens G-protein signalling modulator 1 (AGS3-like, C. elegans). | 1,223 | 8,098 | 7,860 | 0,000 | 0,001 |
| DAPP1 | Homo sapiens dual adaptor of phosphotyrosine and 3-phosphoinositides. | 1,223 | 10,261 | 7,648 | 0,000 | 0,001 |
| MFSD10 | Homo sapiens major facilitator superfamily domain containing 10. | 1,221 | 11,000 | 7,700 | 0,000 | 0,001 |
| PI4KAP2 | Homo sapiens phosphatidylinositol 4-kinase, catalytic α-polypeptide pseudogene 2. | 1,221 | 9,216 | 8,185 | 0,000 | 0,001 |
| LOC100132585 | PREDICTED: Homo sapiens hypothetical protein LOC100132585. | 1,220 | 8,500 | 4,722 | 0,001 | 0,008 |
| LOC646723 | PREDICTED: Homo sapiens similar to Keratin, type I cytoskeletal 18 (Cytokeratin-18) (CK-18) (Keratin-18) (K18). | 1,219 | 11,426 | 4,080 | 0,002 | 0,015 |
| CATSPER2 | Homo sapiens cation channel, sperm associated 2, trans. var. 4. | 1,218 | 9,435 | 4,842 | 0,001 | 0,007 |
| BAMBI | Homo sapiens BMP and activin membrane-bound inhibitor homolog (Xenopus laevis). | 1,218 | 8,499 | 6,808 | 0,000 | 0,002 |
| ITGB4 | Homo sapiens integrin β-4, trans. var. 2. | 1,217 | 11,228 | 7,581 | 0,000 | 0,001 |
| RRAS | Homo sapiens related RAS viral (r-ras) oncogene homolog. | 1,215 | 10,023 | 5,439 | 0,000 | 0,004 |
| LOC100128505 | PREDICTED: Homo sapiens similar to hCG2021201. | 1,214 | 11,983 | 5,765 | 0,000 | 0,003 |
| ATP2B4 | Homo sapiens ATPase. Ca++ transporting. plasma membrane 4, trans. var. 1. | 1,214 | 8,785 | 6,218 | 0,000 | 0,002 |
| LCP1 | Homo sapiens lymphocyte cytosolic protein 1 (L-plastin). | 1,214 | 9,934 | 9,956 | 0,000 | 0,000 |
| AIRE | Homo sapiens autoimmune regulator (autoimmune polyendocrinopathy candidiasis ectodermal dystrophy), trans. var. AIRE-1. | 1,212 | 10,343 | 5,465 | 0,000 | 0,004 |
| ITGB4 | Homo sapiens integrin beta 4, trans. var. 3. | 1,212 | 10,265 | 7,777 | 0,000 | 0,001 |
| LOC728903 | PREDICTED: Homo sapiens hypothetical LOC728903 trans. var. 1. | 1,211 | 9,154 | 5,354 | 0,000 | 0,005 |
| BLZF1 | Homo sapiens basic leucine zipper nuclear factor 1. | 1,211 | 9,326 | 5,035 | 0,001 | 0,006 |
| FAM175A | Homo sapiens family with sequence similarity 175. member A. | 1,210 | 9,154 | 4,935 | 0,001 | 0,006 |
| STK17B | Homo sapiens serine/threonine kinase 17b. | 1,210 | 8,333 | 6,320 | 0,000 | 0,002 |
| LOC100190938 | Homo sapiens hypothetical LOC100190938, trans. var. 2, non-coding RNA. | 1,207 | 11,349 | 4,436 | 0,001 | 0,010 |
| NUMB | Homo sapiens numb homolog (Drosophila), trans. var. 3. | 1,207 | 9,594 | 6,343 | 0,000 | 0,002 |
| GFPT1 | Homo sapiens glutamine-fructose-6-phosphate transaminase 1. | 1,207 | 9,306 | 14,309 | 0,000 | 0,000 |
| WSB1 | Homo sapiens WD repeat and SOCS box-containing 1, trans. var. 2. | 1,204 | 9,867 | 5,966 | 0,000 | 0,003 |
| RNF44 | Homo sapiens ring finger protein 44. | 1,204 | 8,572 | 9,651 | 0,000 | 0,000 |
| ACSS2 | Homo sapiens acyl-CoA synthetase short-chain family member 2, trans. var. 1. | 1,204 | 9,381 | 5,036 | 0,001 | 0,006 |
| SQSTM1 | Homo sapiens sequestosome 1. | 1,204 | 12,843 | 8,662 | 0,000 | 0,001 |
| LOC643031 | PREDICTED: Homo sapiens similar to NADH dehydrogenase subunit 5. | 1,203 | 11,464 | 5,144 | 0,001 | 0,005 |
| USP36 | Homo sapiens ubiquitin specific peptidase 36. | 1,198 | 8,135 | 5,002 | 0,001 | 0,006 |
| PIGA | Homo sapiens phosphatidylinositol glycan anchor biosynthesis, class A, trans. var. 3. | 1,198 | 8,602 | 8,970 | 0,000 | 0,001 |
| ANXA3 | Homo sapiens annexin A3. | 1,197 | 9,516 | 5,190 | 0,000 | 0,005 |
| ASAM | Homo sapiens adipocyte-specific adhesion molecule. | 1,197 | 8,115 | 3,403 | 0,007 | 0,032 |
| MTSS1 | Homo sapiens metastasis suppressor 1. | 1,196 | 9,941 | 5,432 | 0,000 | 0,004 |
| PGM3 | Homo sapiens phosphoglucomutase 3. | 1,196 | 9,670 | 11,161 | 0,000 | 0,000 |
| EHD1 | Homo sapiens EH-domain containing 1. | 1,195 | 9,610 | 8,238 | 0,000 | 0,001 |
| HIATL2 | Homo sapiens hippocampus abundant transcript-like 2, non-coding RNA. | 1,195 | 9,810 | 5,185 | 0,000 | 0,005 |
| KLHL21 | Homo sapiens kelch-like 21 (Drosophila). | 1,195 | 8,432 | 4,333 | 0,002 | 0,011 |
| NET1 | Homo sapiens neuroepithelial cell transforming 1, trans. var. 2. | 1,195 | 9,342 | 5,008 | 0,001 | 0,006 |
| WDR45 | Homo sapiens WD repeat domain 45, trans. var. 1 | 1,194 | 8,093 | 9,315 | 0,000 | 0,001 |
| SPATA2L | Homo sapiens spermatogenesis associated 2-like. | 1,193 | 8,316 | 9,668 | 0,000 | 0,000 |
| KCNH6 | Homo sapiens potassium voltage-gated channel, subfamily H (eag-related). member 6, trans. var. 1. | 1,193 | 11,311 | 5,563 | 0,000 | 0,004 |
| FLJ36131 | PREDICTED: Homo sapiens hypothetical protein FLJ36131, trans. var. 2. | 1,192 | 8,906 | 5,843 | 0,000 | 0,003 |
| DAB2 | Homo sapiens disabled homolog 2, mitogen-responsive phosphoprotein (Drosophila). | 1,191 | 8,301 | 4,432 | 0,001 | 0,010 |
| ITGA3 | Homo sapiens integrin α-3 (antigen CD49C. α-3 subunit of VLA-3 receptor) trans. var. a. | 1,191 | 11,439 | 7,183 | 0,000 | 0,001 |
| PGM3 | Homo sapiens phosphoglucomutase 3. | 1,191 | 9,085 | 11,465 | 0,000 | 0,000 |
| PSME4 | Homo sapiens proteasome (prosome macropain) activator subunit 4. | 1,189 | 10,139 | 3,955 | 0,003 | 0,017 |
| HNRNPU | Homo sapiens heterogeneous nuclear ribonucleoprotein U (scaffold attachment factor A), trans. var. 2. | 1,189 | 8,486 | 5,222 | 0,000 | 0,005 |
| LOC100128084 | PREDICTED: Homo sapiens hypothetical protein LOC100128084. | 1,188 | 10,818 | 5,774 | 0,000 | 0,003 |
| TXNRD1 | Homo sapiens thioredoxin reductase 1, trans. var. 5. | 1,188 | 9,793 | 4,433 | 0,001 | 0,010 |
| MXD4 | Homo sapiens MAX dimerization protein 4. | 1,188 | 8,372 | 3,128 | 0,011 | 0,044 |
| UBR4 | Homo sapiens ubiquitin protein ligase E3 component n-recognin 4. | 1,188 | 8,804 | 6,796 | 0,000 | 0,002 |
| GRIPAP1 | Homo sapiens GRIP1 associated protein 1, trans. var. 2. | 1,187 | 9,419 | 5,737 | 0,000 | 0,003 |
| ACSS2 | Homo sapiens acyl-CoA synthetase short-chain family member 2, trans. var. 2. | 1,185 | 9,184 | 4,850 | 0,001 | 0,007 |
| HGS | Homo sapiens hepatocyte growth factor-regulated tyrosine kinase substrate. | 1,184 | 10,579 | 5,441 | 0,000 | 0,004 |
| CRCP | Homo sapiens CGRP receptor component, trans. var. 1. | 1,184 | 9,153 | 4,020 | 0,003 | 0,016 |
| PPP2R2C | Homo sapiens protein phosphatase 2 (formerly 2A), regulatory subunit B, gamma isoform, trans. var. 2. | 1,184 | 8,453 | 4,633 | 0,001 | 0,008 |
| HCG2P7 | Homo sapiens HLA complex group 2 pseudogene 7, non-coding RNA. | 1,183 | 11,063 | 5,585 | 0,000 | 0,004 |
| NET1 | Homo sapiens neuroepithelial cell transforming 1, trans. var. 1. | 1,181 | 11,644 | 5,101 | 0,001 | 0,006 |
| MCM8 | Homo sapiens minichromosome maintenance complex component 8, trans. var. 1. | 1,180 | 10,717 | 4,374 | 0,002 | 0,011 |
| LOC644250 | PREDICTED: Homo sapiens hypothetical protein LOC644250. | 1,178 | 8,427 | 7,610 | 0,000 | 0,001 |
| BMS1P5 | Homo sapiens BMS1 pseudogene 5, non-coding RNA. | 1,176 | 10,437 | 5,066 | 0,001 | 0,006 |
| METRNL | PREDICTED: Homo sapiens meteorin, glial cell differentiation regulator-like. | 1,174 | 7,976 | 9,009 | 0,000 | 0,001 |
| DEM1 | Homo sapiens defects in morphology 1 homolog (S. cerevisiae). | 1,174 | 8,747 | 5,636 | 0,000 | 0,004 |
| AXUD1 | Homo sapiens AXIN1 up-regulated 1. | 1,174 | 8,431 | 4,076 | 0,002 | 0,015 |
| S100A2 | Homo sapiens S100 calcium binding protein A2. | 1,173 | 11,005 | 4,274 | 0,002 | 0,012 |
| TRIM8 | Homo sapiens tripartite motif-containing 8. | 1,173 | 10,104 | 6,339 | 0,000 | 0,002 |
| CDKN2AIPNL | Homo sapiens CDKN2A interacting protein N-terminal like. | 1,172 | 11,382 | 4,851 | 0,001 | 0,007 |
| LOC100129211 | PREDICTED: Homo sapiens hypothetical protein LOC100129211. | 1,171 | 8,781 | 6,692 | 0,000 | 0,002 |
| TIMP1 | Homo sapiens TIMP metallopeptidase inhibitor 1. | 1,169 | 10,413 | 5,197 | 0,000 | 0,005 |
| LOC653086 | PREDICTED: Homo sapiens similar to RAN-binding protein 2-like 1 isoform 2 trans. var. 10. | 1,169 | 9,148 | 5,318 | 0,000 | 0,005 |
| SH3KBP1 | Homo sapiens SH3-domain kinase binding protein 1, trans. var. 1. | 1,169 | 9,478 | 6,024 | 0,000 | 0,003 |
| DDIT3 | Homo sapiens DNA-damage-inducible transcript 3. | 1,168 | 8,132 | 8,347 | 0,000 | 0,001 |
| ZNF738 | PREDICTED: Homo sapiens misc_RNA, partial miscRNA. | 1,168 | 9,222 | 5,246 | 0,000 | 0,005 |
| HSD17B7 | Homo sapiens hydroxysteroid (17-β) dehydrogenase 7. | 1,167 | 9,532 | 6,438 | 0,000 | 0,002 |
| MAD1L1 | Homo sapiens MAD1 mitotic arrest deficient-like 1 (yeast), trans. var. 2. | 1,167 | 8,490 | 4,506 | 0,001 | 0,010 |
| DNAJB9 | Homo sapiens DnaJ (Hsp40) homolog, subfamily B, member 9. | 1,164 | 8,191 | 25,461 | 0,000 | 0,000 |
| FCAR | Homo sapiens Fc fragment of IgA, receptor for trans. var. 9. | 1,163 | 8,407 | 7,760 | 0,000 | 0,001 |
| C21orf24 | Homo sapiens chromosome 21 open reading frame 24. | 1,161 | 10,169 | 4,544 | 0,001 | 0,009 |
| JUP | Homo sapiens junction plakoglobin, trans. var. 1. | 1,161 | 11,848 | 6,527 | 0,000 | 0,002 |
| C14orf85 | Homo sapiens chromosome 14 open reading frame 85, non-coding RNA. | 1,157 | 11,172 | 6,599 | 0,000 | 0,002 |
| LOC441087 | Homo sapiens hypothetical gene supported by AK125735. | 1,157 | 11,547 | 7,355 | 0,000 | 0,001 |
| JUNB | Homo sapiens jun B proto-oncogene. | 1,154 | 9,385 | 3,630 | 0,005 | 0,024 |
| ASS1 | Homo sapiens argininosuccinate synthetase 1, trans. var. 2. | 1,153 | 9,128 | 5,413 | 0,000 | 0,004 |
| CCNL1 | Homo sapiens cyclin L1. | 1,152 | 9,073 | 6,210 | 0,000 | 0,002 |
| PNPT1 | Homo sapiens polyribonucleotide nucleotidyltransferase 1. | 1,152 | 9,051 | 5,802 | 0,000 | 0,003 |
| LOC100130445 | PREDICTED: Homo sapiens similar to AML-associated zinc finger protein. | 1,152 | 10,897 | 5,503 | 0,000 | 0,004 |
| P4HA2 | Homo sapiens prolyl 4-hydroxylase α-polypeptide II, trans. var. 2. | 1,152 | 10,806 | 4,988 | 0,001 | 0,006 |
| GLIPR1 | Homo sapiens GLI pathogenesis-related 1. | 1,151 | 8,351 | 9,070 | 0,000 | 0,001 |
| DUSP19 | Homo sapiens dual specificity phosphatase 19. | 1,150 | 10,322 | 6,390 | 0,000 | 0,002 |
| CSNK1D | Homo sapiens casein kinase 1 delta, trans. var. 1. | 1,147 | 9,458 | 7,497 | 0,000 | 0,001 |
| STX3 | Homo sapiens syntaxin 3. | 1,146 | 8,554 | 3,933 | 0,003 | 0,017 |
| H1F0 | Homo sapiens H1 histone family member 0. | 1,144 | 10,617 | 3,782 | 0,004 | 0,020 |
| C15orf63 | Homo sapiens chromosome 15 open reading frame 63. | 1,144 | 9,579 | 5,524 | 0,000 | 0,004 |
| C5orf41 | Homo sapiens chromosome 5 open reading frame 41. | 1,143 | 7,779 | 6,749 | 0,000 | 0,002 |
| C5orf41 | Homo sapiens chromosome 5 open reading frame 41. | 1,141 | 7,737 | 8,372 | 0,000 | 0,001 |
| ARHGEF4 | Homo sapiens Rho guanine nucleotide exchange factor (GEF) 4, trans. var. 1. | 1,141 | 8,427 | 3,285 | 0,009 | 0,036 |
| RHBDF1 | Homo sapiens rhomboid 5 homolog 1 (Drosophila). | 1,140 | 9,619 | 5,930 | 0,000 | 0,003 |
| LOC392437 | PREDICTED: Homo sapiens misc_RNA, miscRNA. | 1,140 | 11,970 | 4,166 | 0,002 | 0,013 |
| GJC1 | Homo sapiens gap junction protein gamma 1. 45kDa, trans. var. 1. | 1,139 | 11,966 | 5,556 | 0,000 | 0,004 |
| ZYX | Homo sapiens zyxin, trans. var. 1. | 1,139 | 9,808 | 7,499 | 0,000 | 0,001 |
| N4BP2 | Homo sapiens Nedd4 binding protein 2. | 1,136 | 8,301 | 6,226 | 0,000 | 0,002 |
| C4orf34 | Homo sapiens chromosome 4 open reading frame 34. | 1,135 | 10,197 | 4,003 | 0,003 | 0,016 |
| EID2B | Homo sapiens EP300 interacting inhibitor of differentiation 2B. | 1,134 | 10,605 | 5,826 | 0,000 | 0,003 |
| YRDC | Homo sapiens yrdC domain containing (E. coli), nuclear gene encoding mitochondrial protein. | 1,134 | 9,986 | 5,065 | 0,001 | 0,006 |
| HSPC268 | Homo sapiens hypothetical protein HSPC268. | 1,133 | 8,902 | 5,642 | 0,000 | 0,004 |
| MAFF | Homo sapiens v-maf musculoaponeurotic fibrosarcoma oncogene homolog F (avian), trans. var. 1. | 1,133 | 7,895 | 9,088 | 0,000 | 0,001 |
| IL10 | Homo sapiens interleukin 10. | 1,132 | 9,574 | 5,409 | 0,000 | 0,004 |
| ZNF598 | Homo sapiens zinc finger protein 598. | 1,132 | 10,335 | 6,898 | 0,000 | 0,002 |
| BAIAP2L1 | Homo sapiens BAI1-associated protein 2-like 1. | 1,131 | 11,099 | 11,077 | 0,000 | 0,000 |
| FLJ46309 | Homo sapiens hypothetical protein LOC649598. | 1,131 | 10,562 | 4,601 | 0,001 | 0,009 |
| FNBP4 | Homo sapiens formin binding protein 4. | 1,129 | 9,550 | 5,303 | 0,000 | 0,005 |
| KLF4 | Homo sapiens Kruppel-like factor 4 (gut). | 1,128 | 8,093 | 7,573 | 0,000 | 0,001 |
| XBP1 | Homo sapiens X-box binding protein 1, trans. var. 2. | 1,127 | 10,794 | 5,917 | 0,000 | 0,003 |
| DDX51 | Homo sapiens DEAD (Asp-Glu-Ala-Asp) box polypeptide 51. | 1,127 | 9,511 | 5,055 | 0,001 | 0,006 |
| CREB1 | Homo sapiens cAMP responsive element binding protein 1, trans. var. A. | 1,126 | 11,487 | 4,688 | 0,001 | 0,008 |
| CHMP1B | Homo sapiens chromatin modifying protein 1B. | 1,126 | 11,641 | 4,847 | 0,001 | 0,007 |
| KIFC2 | Homo sapiens kinesin family member C2. | 1,125 | 9,134 | 7,486 | 0,000 | 0,001 |
| LOC100128062 | PREDICTED: Homo sapiens misc_RNA, miscRNA. | 1,122 | 9,471 | 6,404 | 0,000 | 0,002 |
| CDCP1 | Homo sapiens CUB domain containing protein 1, trans. var. 2. | 1,118 | 8,047 | 3,904 | 0,003 | 0,018 |
| CCBE1 | Homo sapiens collagen and calcium binding EGF domains 1. | 1,117 | 8,475 | 5,274 | 0,000 | 0,005 |
| LOC285741 | PREDICTED: Homo sapiens misc_RNA, miscRNA. | 1,113 | 9,881 | 4,175 | 0,002 | 0,013 |
| SLC44A4 | Homo sapiens solute carrier family 44, member 4, trans. var. 2. | 1,113 | 11,253 | 5,684 | 0,000 | 0,004 |
| COL7A1 | Homo sapiens collagen, type VII, α-1 (epidermolysis bullosa. dystrophic. dominant and recessive). | 1,112 | 11,535 | 4,783 | 0,001 | 0,007 |
| CLIP2 | Homo sapiens CAP-GLY domain containing linker protein 2, trans. var. 2. | 1,112 | 8,582 | 7,474 | 0,000 | 0,001 |
| P4HA2 | Homo sapiens prolyl 4-hydroxylase, α-polypeptide II, trans. var. 3. | 1,108 | 9,746 | 4,466 | 0,001 | 0,010 |
| SCG5 | Homo sapiens secretogranin V (7B2 protein). | 1,107 | 7,835 | 3,385 | 0,007 | 0,032 |
| LYPD3 | Homo sapiens LY6/PLAUR domain containing 3. | 1,105 | 7,994 | 3,873 | 0,003 | 0,018 |
| FAM107B | Homo sapiens family with sequence similarity 107, member B. | 1,104 | 8,757 | 7,609 | 0,000 | 0,001 |
| LOC653489 | PREDICTED: Homo sapiens similar to Ran-binding protein 2 (RanBP2) (Nuclear pore complex protein Nup358) (Nucleoporin Nup358) (358 kDa nucleoporin) (P270), trans. var. 7. | 1,104 | 8,740 | 8,451 | 0,000 | 0,001 |
| VIL2 | Homo sapiens villin 2 (ezrin). | 1,102 | 12,483 | 10,919 | 0,000 | 0,000 |
| SEMA3E | Homo sapiens sema domain, immunoglobulin domain (Ig), short basic domain, secreted, (semaphorin) 3E. | 1,101 | 9,163 | 9,302 | 0,000 | 0,001 |
| SH2D5 | PREDICTED: Homo sapiens SH2 domain containing 5. | 1,100 | 8,273 | 5,343 | 0,000 | 0,005 |
| PIM3 | PREDICTED: Homo sapiens pim-3 oncogene. | 1,099 | 8,523 | 8,630 | 0,000 | 0,001 |
| LOC389787 | PREDICTED: Homo sapiens similar to Translationally-controlled tumor protein (TCTP) (p23) (Histamine-releasing factor) (HRF) (Fortilin). | 1,098 | 11,060 | 5,080 | 0,001 | 0,006 |
| LOC100128098 | PREDICTED: Homo sapiens hypothetical protein LOC100128098. | 1,096 | 8,459 | 4,163 | 0,002 | 0,013 |
| FTHL8 | Homo sapiens ferritin, heavy polypeptide-like 8, on chromosome X. | 1,092 | 11,056 | 3,264 | 0,009 | 0,037 |
| RAB11FIP5 | Homo sapiens RAB11 family interacting protein 5 (class I). | 1,091 | 8,320 | 5,411 | 0,000 | 0,004 |
| IL24 | Homo sapiens interleukin 24, trans. var. 1. | 1,090 | 7,663 | 3,626 | 0,005 | 0,024 |
| OCIAD1 | Homo sapiens OCIA domain containing 1, trans. var. 5. | 1,087 | 9,954 | 5,256 | 0,000 | 0,005 |
| FKTN | Homo sapiens fukutin, trans. var. 2. | 1,086 | 11,373 | 5,515 | 0,000 | 0,004 |
| SFRS17A | Homo sapiens splicing factor, arginine/serine-rich 17A, trans. var. 1. | 1,084 | 8,756 | 7,390 | 0,000 | 0,001 |
| LOC100134159 | PREDICTED: Homo sapiens similar to Coiled-coil domain containing 144B. | 1,083 | 9,429 | 4,678 | 0,001 | 0,008 |
| LTBP4 | Homo sapiens latent transforming growth factor beta binding protein 4, trans. var. 3. | 1,083 | 9,208 | 7,267 | 0,000 | 0,001 |
| KLHL24 | Homo sapiens kelch-like 24 (Drosophila). | 1,083 | 8,366 | 3,578 | 0,005 | 0,026 |
| GFPT2 | Homo sapiens glutamine-fructose-6-phosphate transaminase 2. | 1,082 | 7,935 | 5,852 | 0,000 | 0,003 |
| SLMO1 | Homo sapiens slowmo homolog 1 (Drosophila) | 1,080 | 9,637 | 5,020 | 0,001 | 0,006 |
| LOC729978 | PREDICTED: Homo sapiens similar to LOC339047 protein, trans. var. 2. | 1,078 | 9,911 | 6,378 | 0,000 | 0,002 |
| RNY4 | Homo sapiens RNA, Ro-associated Y4 (RNY4), small cytoplasmic RNA. | 1,078 | 8,765 | 4,903 | 0,001 | 0,007 |
| LOC729252 | PREDICTED: Homo sapiens similar to Keratin, type I cytoskeletal 14 (Cytokeratin-14) (CK-14) (Keratin-14) (K14). | 1,078 | 8,236 | 4,521 | 0,001 | 0,009 |
| IFFO1 | Homo sapiens intermediate filament family orphan 1, trans. var. 2. | 1,077 | 8,004 | 6,606 | 0,000 | 0,002 |
| NAGK | Homo sapiens N-acetylglucosamine kinase. | 1,076 | 8,926 | 6,738 | 0,000 | 0,002 |
| EIF1B | Homo sapiens eukaryotic translation initiation factor 1B. | 1,075 | 9,594 | 6,945 | 0,000 | 0,002 |
| FAM119A | Homo sapiens family with sequence similarity 119, member A. | 1,073 | 10,522 | 5,859 | 0,000 | 0,003 |
| KLF11 | PREDICTED: Homo sapiens Kruppel-like factor 11. | 1,073 | 8,014 | 5,428 | 0,000 | 0,004 |
| LOC392437 | PREDICTED: Homo sapiens misc_RNA, miscRNA. | 1,071 | 12,782 | 3,982 | 0,003 | 0,016 |
| FLJ35390 | Homo sapiens hypothetical LOC255031, trans. var. 1. non-coding RNA. | 1,069 | 8,696 | 4,975 | 0,001 | 0,006 |
| CHRNA5 | Homo sapiens cholinergic receptor, nicotinic α-5. | 1,068 | 9,087 | 4,984 | 0,001 | 0,006 |
| SH3BGRL3 | Homo sapiens SH3 domain binding glutamic acid-rich protein like 3. | 1,066 | 11,011 | 3,594 | 0,005 | 0,025 |
| CITED2 | Homo sapiens Cbp/p300-interacting transactivator, with Glu/Asp-rich carboxy-terminal domain 2, trans. var. 1. | 1,065 | 8,560 | 7,369 | 0,000 | 0,001 |
| C16orf72 | Homo sapiens chromosome 16 open reading frame 72. | 1,065 | 8,979 | 7,357 | 0,000 | 0,001 |
| SOX9 | Homo sapiens SRY (sex determining region Y)-box 9 (campomelic dysplasia. autosomal sex-reversal). | 1,065 | 9,079 | 3,243 | 0,009 | 0,038 |
| ABTB1 | Homo sapiens ankyrin repeat and BTB (POZ) domain containing 1, trans. var. 1. | 1,064 | 7,766 | 5,721 | 0,000 | 0,004 |
| CCDC130 | Homo sapiens coiled-coil domain containing 130. | 1,063 | 9,353 | 7,404 | 0,000 | 0,001 |
| VIM | Homo sapiens vimentin. | 1,057 | 11,382 | 4,175 | 0,002 | 0,013 |
| LOC729231 | PREDICTED: Homo sapiens misc_RNA, miscRNA. | 1,057 | 8,253 | 6,465 | 0,000 | 0,002 |
| AXIN1 | Homo sapiens axin 1, trans. var. 2. | 1,056 | 9,264 | 9,057 | 0,000 | 0,001 |
| C20orf199 | Homo sapiens chromosome 20 open reading frame 199, trans. var. 3, non-coding RNA. | 1,053 | 11,847 | 5,979 | 0,000 | 0,003 |
| UGDH | Homo sapiens UDP-glucose dehydrogenase. | 1,053 | 9,372 | 12,473 | 0,000 | 0,000 |
| MGLL | Homo sapiens monoglyceride lipase, trans. var. 1. | 1,052 | 8,003 | 3,124 | 0,011 | 0,044 |
| HES4 | Homo sapiens hairy and enhancer of split 4 (Drosophila). | 1,051 | 9,317 | 4,681 | 0,001 | 0,008 |
| FGD6 | Homo sapiens FYVE, RhoGEF and PH domain containing 6. | 1,051 | 8,120 | 8,707 | 0,000 | 0,001 |
| LOC642469 | PREDICTED: Homo sapiens misc_RNA, miscRNA. | 1,051 | 8,616 | 5,616 | 0,000 | 0,004 |
| ETS1 | Homo sapiens v-ets erythroblastosis virus E26 oncogene homolog 1 (avian). | 1,050 | 8,998 | 7,166 | 0,000 | 0,001 |
| DSC2 | Homo sapiens desmocollin 2, trans. var. Dsc2b. | 1,050 | 9,122 | 3,707 | 0,004 | 0,022 |
| CASZ1 | Homo sapiens castor zinc finger 1, trans. var. 2. | 1,046 | 8,090 | 4,745 | 0,001 | 0,008 |
| LOC728620 | PREDICTED: Homo sapiens misc_RNA, miscRNA. | 1,045 | 11,725 | 5,986 | 0,000 | 0,003 |
| IRF6 | Homo sapiens interferon regulatory factor 6. | 1,044 | 8,353 | 6,284 | 0,000 | 0,002 |
| WASL | Homo sapiens Wiskott-Aldrich syndrome-like. | 1,044 | 9,058 | 8,042 | 0,000 | 0,001 |
| AVPI1 | Homo sapiens arginine vasopressin-induced 1. | 1,042 | 9,584 | 3,722 | 0,004 | 0,022 |
| FAM43A | Homo sapiens family with sequence similarity 43. member A. | 1,042 | 8,356 | 3,233 | 0,010 | 0,038 |
| LRP10 | Homo sapiens low density lipoprotein receptor-related protein 10. | 1,042 | 10,120 | 5,194 | 0,000 | 0,005 |
| VPS37B | Homo sapiens vacuolar protein sorting 37 homolog B (S. cerevisiae). | 1,041 | 9,622 | 5,085 | 0,001 | 0,006 |
| FAM63A | Homo sapiens family with sequence similarity 63, member A, trans. var. 1. | 1,040 | 8,358 | 6,646 | 0,000 | 0,002 |
| QRFPR | Homo sapiens pyroglutamylated RFamide peptide receptor. | 1,039 | 9,840 | 5,336 | 0,000 | 0,005 |
| NFIL3 | Homo sapiens nuclear factor, interleukin 3 regulated. | 1,037 | 8,650 | 7,338 | 0,000 | 0,001 |
| FRMD6 | Homo sapiens FERM domain containing 6. | 1,037 | 9,687 | 6,801 | 0,000 | 0,002 |
| SERPINB8 | Homo sapiens serpin peptidase inhibitor, clade B (ovalbumin), member 8, trans. var. 2. | 1,036 | 8,088 | 6,217 | 0,000 | 0,002 |
| PINK1 | Homo sapiens PTEN induced putative kinase 1 (PINK1), nuclear gene encoding mitochondrial protein. | 1,036 | 8,970 | 3,692 | 0,004 | 0,022 |
| PIP5K2B | Homo sapiens phosphatidylinositol-4-phosphate 5-kinase, type IIβ, trans. var. 2. | 1,035 | 8,657 | 4,415 | 0,001 | 0,010 |
| FLJ44124 | Homo sapiens hypothetical protein LOC641737. | 1,034 | 9,753 | 4,894 | 0,001 | 0,007 |
| GABPB2 | Homo sapiens GA binding protein transcription factor, β-subunit 2. | 1,033 | 12,070 | 5,419 | 0,000 | 0,004 |
| FOXD1 | Homo sapiens forkhead box D1. | 1,033 | 8,726 | 4,202 | 0,002 | 0,013 |
| NBPF20 | Homo sapiens neuroblastoma breakpoint family, member 20. | 1,033 | 8,751 | 4,037 | 0,003 | 0,015 |
| EIF2C2 | Homo sapiens eukaryotic translation initiation factor 2C. 2. | 1,031 | 9,548 | 5,768 | 0,000 | 0,003 |
| ABTB1 | Homo sapiens ankyrin repeat and BTB (POZ) domain containing 1, trans. var. 3. | 1,031 | 7,823 | 5,571 | 0,000 | 0,004 |
| BCL2L1 | Homo sapiens BCL2-like 1, nuclear gene encoding mitochondrial protein, trans. var. 1. | 1,031 | 11,438 | 6,319 | 0,000 | 0,002 |
| ADRB2 | Homo sapiens adrenergic β-2- receptor surface. | 1,030 | 8,232 | 4,568 | 0,001 | 0,009 |
| RRAGC | Homo sapiens Ras-related GTP binding C. | 1,029 | 8,972 | 5,617 | 0,000 | 0,004 |
| HAS3 | Homo sapiens hyaluronan synthase 3, trans. var. 1. | 1,028 | 9,971 | 5,270 | 0,000 | 0,005 |
| PIK4CA | Homo sapiens phosphatidylinositol 4-kinase, catalytic α polypeptide, trans. var. 1. | 1,028 | 9,396 | 9,065 | 0,000 | 0,001 |
| ANXA2P1 | Homo sapiens annexin A2 pseudogene 1 on chromosome 4. | 1,028 | 11,728 | 4,981 | 0,001 | 0,006 |
| LOC390345 | PREDICTED: Homo sapiens misc_RNA, miscRNA. | 1,027 | 12,147 | 4,732 | 0,001 | 0,008 |
| BPGM | Homo sapiens 2.3-bisphosphoglycerate mutase, trans. var. 1. | 1,026 | 8,481 | 3,456 | 0,007 | 0,030 |
| WARS | Homo sapiens tryptophanyl-tRNA synthetase, trans. var. 1. | 1,026 | 9,495 | 3,023 | 0,013 | 0,050 |
| SDC4 | Homo sapiens syndecan 4. | 1,026 | 10,213 | 5,796 | 0,000 | 0,003 |
| CDAN1 | Homo sapiens congenital dyserythropoietic anemia, type I. | 1,022 | 11,086 | 5,173 | 0,000 | 0,005 |
| EDEM1 | Homo sapiens ER degradation enhancer, mannosidase α-like 1. | 1,022 | 8,632 | 7,487 | 0,000 | 0,001 |
| RAB11FIP1 | Homo sapiens RAB11 family interacting protein 1 (class I), trans. var. 3. | 1,021 | 8,395 | 5,165 | 0,000 | 0,005 |
| LOC729090 | PREDICTED: Homo sapiens similar to Eukaryotic translation elongation factor 1, α 1. | 1,019 | 12,127 | 5,287 | 0,000 | 0,005 |
| TMEM44 | Homo sapiens transmembrane protein 44, trans. var. 1. | 1,017 | 8,669 | 3,816 | 0,004 | 0,019 |
| DGKA | Homo sapiens diacylglycerol kinase α, 80kDa, trans. var. 4. | 1,017 | 8,866 | 3,576 | 0,005 | 0,026 |
| SDHALP1 | Homo sapiens succinate dehydrogenase complex, subunit A, flavoprotein pseudogene 1 on chromosome 3. | 1,016 | 9,399 | 4,467 | 0,001 | 0,010 |
| CSF2RA | Homo sapiens colony stimulating factor 2 receptor-α, low-affinity (granulocyte-macrophage), trans. var. 6. | 1,016 | 9,575 | 5,909 | 0,000 | 0,003 |
| HMGA1 | Homo sapiens high mobility group AT-hook 1, trans. var. 1. | 1,016 | 12,001 | 3,132 | 0,011 | 0,043 |
| HBP1 | Homo sapiens HMG-box transcription factor 1. | 1,014 | 8,097 | 5,185 | 0,000 | 0,005 |
| AKR1D1 | Homo sapiens aldo-keto reductase family 1, member D1 (delta 4-3-ketosteroid-5-β-reductase). | 1,013 | 11,731 | 6,054 | 0,000 | 0,003 |
| CDK5R1 | Homo sapiens cyclin-dependent kinase 5, regulatory subunit 1 (p35). | 1,013 | 8,798 | 4,103 | 0,002 | 0,014 |
| LOC100133177 | PREDICTED: Homo sapiens misc_RNA, miscRNA. | 1,012 | 11,555 | 4,947 | 0,001 | 0,006 |
| RPPH1 | Homo sapiens ribonuclease P RNA component H1, RNase P RNA. | 1,012 | 7,653 | 5,955 | 0,000 | 0,003 |
| LOC100129269 | PREDICTED: Homo sapiens hypothetical protein LOC100129269. | 1,011 | 8,600 | 4,192 | 0,002 | 0,013 |
| ETV5 | Homo sapiens ets variant gene 5 (ets-related molecule). | 1,011 | 9,080 | 5,630 | 0,000 | 0,004 |
| PTPRE | Homo sapiens protein tyrosine phosphatase. receptor type. E, trans. var. 2. | 1,011 | 8,474 | 7,207 | 0,000 | 0,001 |
| LYN | Homo sapiens v-yes-1 Yamaguchi sarcoma viral related oncogene homolog. | 1,009 | 8,869 | 9,907 | 0,000 | 0,000 |
| DHRS7 | Homo sapiens dehydrogenase/reductase (SDR family) member 7. | 1,008 | 10,289 | 4,318 | 0,002 | 0,011 |
| CYLN2 | Homo sapiens cytoplasmic linker 2, trans. var. 2. | 1,007 | 8,674 | 5,952 | 0,000 | 0,003 |
| SHCBP1 | Homo sapiens SHC SH2-domain binding protein 1. | 1,007 | 11,394 | 6,807 | 0,000 | 0,002 |
| ZNF652 | Homo sapiens zinc finger protein 652. | 1,006 | 9,498 | 5,896 | 0,000 | 0,003 |
| SEC16A | Homo sapiens SEC16 homolog A (S. cerevisiae). | 1,004 | 9,352 | 4,710 | 0,001 | 0,008 |
| MANBA | Homo sapiens mannosidase β-A, lysosomal. | 1,004 | 8,735 | 4,087 | 0,002 | 0,015 |
| PODXL | Homo sapiens podocalyxin-like, trans. var. 1. | 1,003 | 7,992 | 7,275 | 0,000 | 0,001 |
| LOC729603 | Homo sapiens calcium binding protein P22 pseudogene, non-coding RNA. | 1,003 | 11,369 | 3,833 | 0,004 | 0,019 |
| SLC38A1 | Homo sapiens solute carrier family 38, member 1, trans. var. 1. | 1,002 | 9,049 | 7,595 | 0,000 | 0,001 |
| B4GALT5 | Homo sapiens UDP-Gal:betaGlcNAc β 1.4- galactosyltransferase, polypeptide 5. | 1,002 | 10,402 | 5,066 | 0,001 | 0,006 |
| LOC100133233 | PREDICTED: Homo sapiens hypothetical protein LOC100133233. | 1,000 | 11,582 | 5,185 | 0,000 | 0,005 |
| LILRB1 | Homo sapiens leukocyte immunoglobulin-like receptor, subfamily B (with TM and ITIM domains) member 1, trans. var. 2. | 1,000 | 10,726 | 4,516 | 0,001 | 0,009 |
| EXOSC9 | Homo sapiens exosome component 9, trans. var. 1. | -1,002 | 8,943 | -5,706 | 0,000 | 0,004 |
| XRCC5 | Homo sapiens X-ray repair complementing defective repair in Chinese hamster cells 5 (double-strand-break rejoining; Ku autoantigen. 80kDa) | -1,002 | 9,000 | -6,661 | 0,000 | 0,002 |
| ALG8 | Homo sapiens asparagine-linked glycosylation 8, α-1.3-glucosyltransferase homolog (S. cerevisiae), trans. var. 2. | -1,002 | 8,645 | -4,561 | 0,001 | 0,009 |
| GGCT | Homo sapiens gamma-glutamyl cyclotransferase. | -1,003 | 10,532 | -4,789 | 0,001 | 0,007 |
| SLC37A4 | Homo sapiens solute carrier family 37 (glucose-6-phosphate transporter). member 4. | -1,007 | 9,250 | -4,301 | 0,002 | 0,012 |
| HNRNPAB | Homo sapiens heterogeneous nuclear ribonucleoprotein A/B, trans. var. 2. | -1,009 | 12,509 | -7,331 | 0,000 | 0,001 |
| NDUFB6 | Homo sapiens NADH dehydrogenase (ubiquinone) 1 β-subcomplex 6, 17kDa, nuclear gene encoding mitochondrial protein, trans. var. 1. | -1,009 | 10,041 | -5,745 | 0,000 | 0,003 |
| PAQR4 | Homo sapiens progestin and adipoQ receptor family member IV. | -1,011 | 9,103 | -6,665 | 0,000 | 0,002 |
| PON2 | Homo sapiens paraoxonase 2, trans. var. 1. | -1,011 | 10,040 | -3,668 | 0,005 | 0,023 |
| C12orf24 | Homo sapiens chromosome 12 open reading frame 24. | -1,013 | 8,307 | -4,122 | 0,002 | 0,014 |
| AKR7A2 | Homo sapiens aldo-keto reductase family 7, member A2 (aflatoxin aldehyde reductase). | -1,015 | 10,379 | -8,932 | 0,000 | 0,001 |
| TMEM203 | Homo sapiens transmembrane protein 203. | -1,016 | 9,545 | -7,656 | 0,000 | 0,001 |
| LYAR | Homo sapiens Ly1 antibody reactive homolog (mouse). | -1,016 | 9,490 | -5,532 | 0,000 | 0,004 |
| PXMP2 | Homo sapiens peroxisomal membrane protein 2. 22kDa. | -1,016 | 8,218 | -4,443 | 0,001 | 0,010 |
| PAFAH1B3 | Homo sapiens platelet-activating factor acetylhydrolase, isoform Ib, gamma subunit 29kDa. | -1,017 | 9,632 | -4,642 | 0,001 | 0,008 |
| PGAM4 | Homo sapiens phosphoglycerate mutase family member 4. | -1,019 | 11,108 | -12,708 | 0,000 | 0,000 |
| PSME3 | Homo sapiens proteasome (prosome macropain) activator subunit 3 (PA28 gamma; Ki), trans. var. 1. | -1,020 | 8,823 | -5,645 | 0,000 | 0,004 |
| MRPL34 | Homo sapiens mitochondrial ribosomal protein L34, nuclear gene encoding mitochondrial protein. | -1,020 | 9,316 | -5,527 | 0,000 | 0,004 |
| GEMIN4 | Homo sapiens gem (nuclear organelle) associated protein 4. | -1,021 | 9,224 | -6,830 | 0,000 | 0,002 |
| POLE3 | Homo sapiens polymerase (DNA directed) epsilon 3 (p17 subunit). | -1,022 | 11,656 | -6,981 | 0,000 | 0,002 |
| CDCA4 | Homo sapiens cell division cycle associated 4, trans. var. 14. | -1,022 | 8,975 | -9,170 | 0,000 | 0,001 |
| POLR3K | Homo sapiens polymerase (RNA) III (DNA directed) polypeptide K, 12.3 kDa. | -1,023 | 8,037 | -6,804 | 0,000 | 0,002 |
| NASP | Homo sapiens nuclear autoantigenic sperm protein (histone-binding), trans. var. 2. | -1,025 | 7,808 | -9,341 | 0,000 | 0,001 |
| TRAP1 | Homo sapiens TNF receptor-associated protein 1. | -1,025 | 9,246 | -3,311 | 0,008 | 0,035 |
| TUBB2C | Homo sapiens tubulin β-2C. | -1,029 | 11,941 | -14,526 | 0,000 | 0,000 |
| MRPL3 | Homo sapiens mitochondrial ribosomal protein L3, nuclear gene encoding mitochondrial protein. | -1,029 | 11,219 | -4,246 | 0,002 | 0,012 |
| APTX | Homo sapiens aprataxin, trans. var. 5. | -1,030 | 8,512 | -5,788 | 0,000 | 0,003 |
| CENPF | Homo sapiens centromere protein F. 350/400ka (mitosin). | -1,030 | 7,981 | -4,214 | 0,002 | 0,013 |
| HIBCH | Homo sapiens 3-hydroxyisobutyryl-Coenzyme A hydrolase, nuclear gene encoding mitochondrial protein, trans. var. 2. | -1,031 | 8,602 | -5,212 | 0,000 | 0,005 |
| RDH16 | Homo sapiens retinol dehydrogenase 16 (all-trans). | -1,032 | 7,665 | -3,951 | 0,003 | 0,017 |
| KIF4A | Homo sapiens kinesin family member 4A. | -1,032 | 8,241 | -3,541 | 0,006 | 0,027 |
| CPSF4 | Homo sapiens cleavage and polyadenylation specific factor 4, 30kDa, trans. var. 2. | -1,035 | 10,312 | -6,574 | 0,000 | 0,002 |
| CCNE2 | Homo sapiens cyclin E2, trans. var. 2. | -1,036 | 7,841 | -8,617 | 0,000 | 0,001 |
| NHP2L1 | Homo sapiens NHP2 non-histone chromosome protein 2-like 1 (S. cerevisiae), trans. var. 2. | -1,036 | 9,100 | -5,323 | 0,000 | 0,005 |
| SNRPF | Homo sapiens small nuclear ribonucleoprotein polypeptide F. | -1,036 | 11,459 | -4,309 | 0,002 | 0,012 |
| MCM7 | Homo sapiens minichromosome maintenance complex component 7, trans. var. 1. | -1,038 | 8,208 | -5,616 | 0,000 | 0,004 |
| CECR5 | Homo sapiens cat eye syndrome chromosome region, candidate 5, trans. var. 2. | -1,038 | 9,561 | -5,561 | 0,000 | 0,004 |
| ACN9 | Homo sapiens ACN9 homolog (S. cerevisiae). | -1,038 | 8,978 | -4,996 | 0,001 | 0,006 |
| EXOSC9 | Homo sapiens exosome component 9, trans. var. 1. | -1,040 | 8,915 | -6,615 | 0,000 | 0,002 |
| STAT1 | Homo sapiens signal transducer and activator of transcription, 1. 91kDa, trans. var. α. | -1,040 | 9,091 | -4,340 | 0,002 | 0,011 |
| ETFB | Homo sapiens electron-transfer-flavoprotein, β-polypeptide, trans. var. 1. | -1,040 | 9,566 | -4,882 | 0,001 | 0,007 |
| DEK | Homo sapiens DEK oncogene (DNA binding). | -1,042 | 9,832 | -4,032 | 0,003 | 0,016 |
| NMRAL1 | Homo sapiens NmrA-like family domain containing 1. | -1,043 | 9,563 | -5,352 | 0,000 | 0,005 |
| RUVBL1 | Homo sapiens RuvB-like 1 (E. coli). | -1,044 | 9,239 | -4,962 | 0,001 | 0,006 |
| TMEM177 | Homo sapiens transmembrane protein 177. | -1,044 | 8,267 | -8,478 | 0,000 | 0,001 |
| SIVA | Homo sapiens CD27-binding (Siva) protein, trans. var. 1. | -1,045 | 9,352 | -6,551 | 0,000 | 0,002 |
| SPR | Homo sapiens sepiapterin reductase (7.8-dihydrobiopterin: NADP+ oxidoreductase) | -1,045 | 8,299 | -6,098 | 0,000 | 0,003 |
| EEF1B2 | Homo sapiens eukaryotic translation elongation factor 1 β 2, trans. var. 2. | -1,046 | 9,543 | -3,918 | 0,003 | 0,017 |
| TNS3 | Homo sapiens tensin 3. | -1,047 | 9,174 | -4,625 | 0,001 | 0,009 |
| GPSM2 | Homo sapiens G-protein signalling modulator 2 (AGS3-like. C. elegans). | -1,050 | 9,048 | -3,726 | 0,004 | 0,022 |
| TOMM40 | Homo sapiens translocase of outer mitochondrial membrane 40 homolog (yeast), nuclear gene encoding mitochondrial protein. | -1,051 | 10,812 | -4,069 | 0,002 | 0,015 |
| NAE1 | Homo sapiens NEDD8 activating enzyme E1 subunit 1, trans. var. 3. | -1,052 | 10,565 | -8,048 | 0,000 | 0,001 |
| SFRS1 | Homo sapiens splicing factor, arginine/serine-rich 1, trans. var. 2. | -1,052 | 10,866 | -4,066 | 0,002 | 0,015 |
| NGFRAP1 | Homo sapiens nerve growth factor receptor (TNFRSF16) associated protein 1, trans. var. 1. | -1,052 | 10,588 | -5,743 | 0,000 | 0,003 |
| HAT1 | Homo sapiens histone acetyltransferase 1, trans. var. 1. | -1,053 | 9,138 | -6,054 | 0,000 | 0,003 |
| D2HGDH | Homo sapiens D-2-hydroxyglutarate dehydrogenase, nuclear gene encoding mitochondrial protein. | -1,053 | 8,014 | -6,503 | 0,000 | 0,002 |
| HNRPA2B1 | Homo sapiens heterogeneous nuclear ribonucleoprotein A2/B1, trans. var. B1. | -1,055 | 12,307 | -5,510 | 0,000 | 0,004 |
| SEPHS1 | Homo sapiens selenophosphate synthetase. | -1,055 | 8,735 | -5,564 | 0,000 | 0,004 |
| GLRX5 | Homo sapiens glutaredoxin 5. | -1,055 | 11,198 | -5,512 | 0,000 | 0,004 |
| SCO1 | Homo sapiens SCO cytochrome oxidase deficient homolog 1 (yeast), nuclear gene encoding mitochondrial protein. | -1,056 | 9,299 | -10,343 | 0,000 | 0,000 |
| SF3A3 | Homo sapiens splicing factor 3a, subunit 3, 60kDa. | -1,056 | 9,315 | -6,038 | 0,000 | 0,003 |
| HMBS | Homo sapiens hydroxymethylbilane synthase, trans. var. 1. | -1,057 | 9,227 | -6,197 | 0,000 | 0,002 |
| CAV2 | Homo sapiens caveolin 2, trans. var. 1. | -1,057 | 10,210 | -3,188 | 0,010 | 0,040 |
| DKC1 | Homo sapiens dyskeratosis congenita 1, dyskerin. | -1,059 | 11,233 | -5,198 | 0,000 | 0,005 |
| HNRNPM | Homo sapiens heterogeneous nuclear ribonucleoprotein M, trans. var. 2. | -1,060 | 10,813 | -8,115 | 0,000 | 0,001 |
| C1orf131 | Homo sapiens chromosome 1 open reading frame 131. | -1,060 | 9,054 | -5,616 | 0,000 | 0,004 |
| UBR7 | Homo sapiens ubiquitin protein ligase E3 component n-recognin 7 (putative), trans. var. 3. | -1,061 | 8,233 | -6,525 | 0,000 | 0,002 |
| CCDC99 | Homo sapiens coiled-coil domain containing 99. | -1,062 | 8,814 | -3,434 | 0,007 | 0,030 |
| LOC728873 | PREDICTED: Homo sapiens misc_RNA, miscRNA. | -1,063 | 11,085 | -3,356 | 0,008 | 0,033 |
| PKM2 | Homo sapiens pyruvate kinase, muscle, trans. var. 2. | -1,063 | 10,506 | -4,379 | 0,002 | 0,011 |
| TMEM109 | Homo sapiens transmembrane protein 109. | -1,063 | 8,365 | -6,439 | 0,000 | 0,002 |
| IDH1 | Homo sapiens isocitrate dehydrogenase 1 (NADP+), soluble. | -1,063 | 8,403 | -5,680 | 0,000 | 0,004 |
| DYNLL1 | Homo sapiens dynein, light chain, LC8-type 1, trans. var. 1. | -1,064 | 11,240 | -6,109 | 0,000 | 0,003 |
| STRA13 | Homo sapiens stimulated by retinoic acid 13 homolog (mouse). | -1,065 | 10,043 | -7,399 | 0,000 | 0,001 |
| MRPL24 | Homo sapiens mitochondrial ribosomal protein L24, nuclear gene encoding mitochondrial protein, trans. var. 1. | -1,066 | 10,051 | -4,450 | 0,001 | 0,010 |
| MRPS28 | Homo sapiens mitochondrial ribosomal protein S28, nuclear gene encoding mitochondrial protein. | -1,066 | 9,093 | -7,958 | 0,000 | 0,001 |
| ABCE1 | Homo sapiens ATP-binding cassette, sub-family E (OABP), member 1, trans. var. 2. | -1,066 | 9,354 | -5,399 | 0,000 | 0,004 |
| TRIM6 | Homo sapiens tripartite motif-containing 6, trans. var. 2. | -1,067 | 7,800 | -14,647 | 0,000 | 0,000 |
| SHMT1 | Homo sapiens serine hydroxymethyltransferase 1 (soluble), trans. var. 1. | -1,069 | 8,035 | -6,499 | 0,000 | 0,002 |
| LDHB | Homo sapiens lactate dehydrogenase B. | -1,073 | 11,403 | -3,405 | 0,007 | 0,031 |
| HSP90AA1 | Homo sapiens heat shock protein 90kDa alpha (cytosolic), class A member 1, trans. var. 1. | -1,073 | 12,182 | -3,209 | 0,010 | 0,039 |
| CASP2 | Homo sapiens caspase 2, apoptosis-related cysteine peptidase, trans. var. 1. | -1,074 | 9,398 | -6,586 | 0,000 | 0,002 |
| SFRS10 | Homo sapiens splicing factor, arginine/serine-rich 10 (transformer 2 homolog, Drosophila). | -1,074 | 9,816 | -5,847 | 0,000 | 0,003 |
| LYPD1 | Homo sapiens LY6/PLAUR domain containing 1, trans. var. 1. | -1,076 | 7,873 | -6,551 | 0,000 | 0,002 |
| SUCLG1 | Homo sapiens succinate-CoA ligase, α subunit. | -1,077 | 9,604 | -5,130 | 0,001 | 0,005 |
| LOC100132715 | PREDICTED: Homo sapiens misc_RNA, miscRNA. | -1,077 | 10,238 | -5,096 | 0,001 | 0,006 |
| COQ9 | Homo sapiens coenzyme Q9 homolog (S. cerevisiae). | -1,078 | 9,792 | -5,063 | 0,001 | 0,006 |
| SIVA1 | Homo sapiens SIVA1, apoptosis-inducing factor, trans. var. 2. | -1,079 | 10,808 | -4,996 | 0,001 | 0,006 |
| USP5 | Homo sapiens ubiquitin specific peptidase 5 (isopeptidase T), trans. var. 2. | -1,080 | 10,168 | -4,959 | 0,001 | 0,006 |
| CHCHD10 | Homo sapiens coiled-coil-helix-coiled-coil-helix domain containing 10. | -1,081 | 9,039 | -3,552 | 0,006 | 0,026 |
| FJX1 | Homo sapiens four jointed box 1 (Drosophila). | -1,082 | 9,753 | -6,543 | 0,000 | 0,002 |
| DERA | Homo sapiens 2-deoxyribose-5-phosphate aldolase homolog (C. elegans). | -1,082 | 10,408 | -5,133 | 0,001 | 0,005 |
| NGFRAP1 | Homo sapiens nerve growth factor receptor (TNFRSF16) associated protein 1, trans. var. 1. | -1,084 | 9,250 | -5,147 | 0,001 | 0,005 |
| KIF23 | Homo sapiens kinesin family member 23, trans. var. 2. | -1,084 | 8,440 | -3,644 | 0,005 | 0,024 |
| SKP2 | Homo sapiens S-phase kinase-associated protein 2 (p45), trans. var. 1. | -1,086 | 7,828 | -8,397 | 0,000 | 0,001 |
| HNRNPAB | Homo sapiens heterogeneous nuclear ribonucleoprotein A/B, trans. var. 2. | -1,086 | 10,899 | -7,364 | 0,000 | 0,001 |
| CKLF | Homo sapiens chemokine-like factor, trans. var. 6. | -1,089 | 9,689 | -3,674 | 0,005 | 0,023 |
| RCN2 | Homo sapiens reticulocalbin 2, EF-hand calcium binding domain. | -1,089 | 9,854 | -4,641 | 0,001 | 0,008 |
| LOC401397 | PREDICTED: Homo sapiens hypothetical LOC401397. | -1,091 | 9,688 | -6,332 | 0,000 | 0,002 |
| LOC100132863 | PREDICTED: Homo sapiens misc_RNA, miscRNA. | -1,091 | 10,303 | -4,150 | 0,002 | 0,014 |
| PGAM1 | Homo sapiens phosphoglycerate mutase 1 (brain). | -1,091 | 10,291 | -9,328 | 0,000 | 0,001 |
| HSPE1 | Homo sapiens heat shock 10kDa protein 1 (chaperonin 10). | -1,092 | 11,372 | -5,365 | 0,000 | 0,005 |
| LOC732007 | PREDICTED: Homo sapiens similar to Phosphoglycerate mutase 1 (Phosphoglycerate mutase isozyme B) (PGAM-B) (BPG-dependent PGAM 1). | -1,093 | 10,554 | -11,367 | 0,000 | 0,000 |
| PAFAH1B1 | Homo sapiens platelet-activating factor acetylhydrolase, isoform Ib, α-subunit 45kDa. | -1,093 | 9,048 | -6,218 | 0,000 | 0,002 |
| SFRS3 | Homo sapiens splicing factor, arginine/serine-rich 3. | -1,093 | 8,541 | -4,852 | 0,001 | 0,007 |
| LOC648695 | PREDICTED: Homo sapiens similar to retinoblastoma binding protein 4, trans. var.5. | -1,093 | 9,475 | -5,491 | 0,000 | 0,004 |
| ALDH7A1 | Homo sapiens aldehyde dehydrogenase 7 family, member A1. | -1,094 | 8,243 | -3,925 | 0,003 | 0,017 |
| LOC730107 | PREDICTED: Homo sapiens similar to Glycine cleavage system H protein, mitochondrial. | -1,095 | 9,989 | -4,162 | 0,002 | 0,013 |
| PFN2 | Homo sapiens profilin 2, trans. var. 1. | -1,095 | 10,962 | -6,273 | 0,000 | 0,002 |
| DTL | Homo sapiens denticleless homolog (Drosophila). | -1,098 | 7,961 | -8,845 | 0,000 | 0,001 |
| PSMA2 | Homo sapiens proteasome (prosome, macropain) subunit, α-type. 2. | -1,099 | 8,390 | -5,864 | 0,000 | 0,003 |
| HNRPR | Homo sapiens heterogeneous nuclear ribonucleoprotein. | -1,099 | 10,285 | -8,206 | 0,000 | 0,001 |
| RRS1 | Homo sapiens RRS1 ribosome biogenesis regulator homolog (S. cerevisiae). | -1,102 | 9,086 | -5,695 | 0,000 | 0,004 |
| GINS3 | Homo sapiens GINS complex subunit 3 (Psf3 homolog). | -1,103 | 8,620 | -7,708 | 0,000 | 0,001 |
| RNASEH2A | Homo sapiens ribonuclease H2. subunit A. | -1,106 | 8,763 | -4,377 | 0,002 | 0,011 |
| TUBA1A | Homo sapiens tubulin α 1a. | -1,106 | 11,968 | -4,714 | 0,001 | 0,008 |
| RNASET2 | Homo sapiens ribonuclease T2. | -1,107 | 9,080 | -3,965 | 0,003 | 0,017 |
| DSCC1 | Homo sapiens defective in sister chromatid cohesion 1 homolog (S. cerevisiae). | -1,108 | 8,207 | -4,279 | 0,002 | 0,012 |
| FOXM1 | Homo sapiens forkhead box M1, trans. var. 2. | -1,109 | 8,405 | -3,859 | 0,003 | 0,019 |
| B3GNT6 | Homo sapiens UDP-GlcNAc:betaGal β-1.3-N-acetylglucosaminyltransferase 6. | -1,112 | 7,933 | -8,023 | 0,000 | 0,001 |
| HNRNPR | Homo sapiens heterogeneous nuclear ribonucleoprotein R, trans. var. 2. | -1,115 | 11,130 | -9,140 | 0,000 | 0,001 |
| CSTF2 | Homo sapiens cleavage stimulation factor, 3' pre-RNA, subunit 2, 64kDa. | -1,116 | 9,111 | -10,354 | 0,000 | 0,000 |
| C14orf156 | Homo sapiens chromosome 14 open reading frame 156. | -1,120 | 12,238 | -6,341 | 0,000 | 0,002 |
| PTPLB | Homo sapiens protein tyrosine phosphatase-like (proline instead of catalytic arginine), member b. | -1,122 | 9,799 | -4,164 | 0,002 | 0,013 |
| C21orf33 | Homo sapiens chromosome 21 open reading frame 33, nuclear gene encoding mitochondrial protein, trans. var. 2. | -1,122 | 9,499 | -4,282 | 0,002 | 0,012 |
| MRPL20 | Homo sapiens mitochondrial ribosomal protein L20, nuclear gene encoding mitochondrial protein. | -1,126 | 9,168 | -8,445 | 0,000 | 0,001 |
| FZD2 | Homo sapiens frizzled homolog 2 (Drosophila). | -1,128 | 8,835 | -9,432 | 0,000 | 0,001 |
| SNHG3-RCC1 | Homo sapiens SNHG3-RCC1 readthrough transcript, trans. var. 1. | -1,129 | 9,188 | -7,179 | 0,000 | 0,001 |
| LOC653874 | PREDICTED: Homo sapiens similar to Dihydrofolate reductase, trans. var. 1. | -1,130 | 8,212 | -4,999 | 0,001 | 0,006 |
| LQK1 | PREDICTED: Homo sapiens misc_RNA, miscRNA. | -1,130 | 8,213 | -7,270 | 0,000 | 0,001 |
| CSE1L | Homo sapiens CSE1 chromosome segregation 1-like (yeast). | -1,130 | 10,638 | -3,841 | 0,004 | 0,019 |
| PGAM1 | Homo sapiens phosphoglycerate mutase 1 (brain). | -1,131 | 11,164 | -14,823 | 0,000 | 0,000 |
| PSME2 | Homo sapiens proteasome (prosome, macropain) activator subunit 2 (PA28 β). | -1,132 | 11,051 | -3,708 | 0,004 | 0,022 |
| MRPL20 | Homo sapiens mitochondrial ribosomal protein L20 (MRPL20), nuclear gene encoding mitochondrial protein. | -1,133 | 9,137 | -7,001 | 0,000 | 0,002 |
| ATP5G1 | Homo sapiens ATP synthase, H+ transporting, mitochondrial F0 complex, sub-unit C1 (subunit 9), nuclear gene encoding mitochondrial protein, trans. var. 2. | -1,135 | 9,844 | -4,988 | 0,001 | 0,006 |
| FEN1 | Homo sapiens flap structure-specific endonuclease 1. | -1,137 | 8,230 | -10,346 | 0,000 | 0,000 |
| ARHGAP23 | PREDICTED: Homo sapiens Rho GTPase activating protein 23, trans. var. 1. | -1,137 | 8,358 | -5,750 | 0,000 | 0,003 |
| IMP3 | Homo sapiens IMP3, U3 small nucleolar ribonucleoprotein homolog (yeast). | -1,138 | 10,327 | -5,429 | 0,000 | 0,004 |
| CCND3 | Homo sapiens cyclin D3. | -1,138 | 8,656 | -6,154 | 0,000 | 0,003 |
| C12orf32 | Homo sapiens chromosome 12 open reading frame 32. | -1,139 | 8,278 | -7,333 | 0,000 | 0,001 |
| TECR | Homo sapiens trans-2.3-enoyl-CoA reductase. | -1,141 | 11,213 | -4,257 | 0,002 | 0,012 |
| LANCL2 | Homo sapiens LanC lantibiotic synthetase component C-like 2 (bacterial). | -1,142 | 8,754 | -6,915 | 0,000 | 0,002 |
| NOL11 | Homo sapiens nucleolar protein 11. | -1,142 | 9,602 | -9,136 | 0,000 | 0,001 |
| NT5DC2 | Homo sapiens 5'-nucleotidase domain containing. | -1,142 | 8,774 | -10,281 | 0,000 | 0,000 |
| PSRC1 | Homo sapiens proline/serine-rich coiled-coil 1, trans. var. 4. | -1,143 | 8,538 | -4,992 | 0,001 | 0,006 |
| IVNS1ABP | Homo sapiens influenza virus NS1A binding protein. | -1,144 | 8,612 | -3,518 | 0,006 | 0,028 |
| PTPLB | Homo sapiens protein tyrosine phosphatase-like (proline instead of catalytic arginine), member b. | -1,144 | 10,121 | -3,712 | 0,004 | 0,022 |
| COMMD4 | Homo sapiens COMM domain containing 4. | -1,144 | 9,196 | -4,666 | 0,001 | 0,008 |
| PRDX2 | Homo sapiens peroxiredoxin 2, nuclear gene encoding mitochondrial protein, trans. var. 3. | -1,145 | 9,077 | -4,924 | 0,001 | 0,006 |
| POLA2 | Homo sapiens polymerase (DNA directed) α-2 (70kD subunit). | -1,145 | 8,187 | -6,770 | 0,000 | 0,002 |
| THOC4 | PREDICTED: Homo sapiens THO complex 4. | -1,145 | 10,021 | -6,448 | 0,000 | 0,002 |
| ACN9 | Homo sapiens ACN9 homolog (S. cerevisiae). | -1,146 | 8,513 | -6,540 | 0,000 | 0,002 |
| RBM14 | Homo sapiens RNA binding motif protein 14. | -1,148 | 9,260 | -5,995 | 0,000 | 0,003 |
| BRIX1 | Homo sapiens BRX1, biogenesis of ribosomes, homolog (S. cerevisiae). | -1,150 | 10,744 | -5,690 | 0,000 | 0,004 |
| MYH10 | Homo sapiens myosin, heavy chain 10, non-muscle. | -1,150 | 9,210 | -4,892 | 0,001 | 0,007 |
| PPIL5 | Homo sapiens peptidylprolyl isomerase (cyclophilin)-like 5, trans. var. 3. | -1,152 | 8,588 | -6,345 | 0,000 | 0,002 |
| DLK2 | Homo sapiens delta-like 2 homolog (Drosophila) (DLK2), trans. var. 2. | -1,153 | 8,480 | -5,396 | 0,000 | 0,004 |
| CDK4 | Homo sapiens cyclin-dependent kinase 4. | -1,157 | 10,104 | -5,245 | 0,000 | 0,005 |
| SIGMAR1 | Homo sapiens sigma non-opioid intracellular receptor 1, trans. var. 1. | -1,159 | 9,745 | -3,660 | 0,005 | 0,023 |
| C1QBP | Homo sapiens complement component 1, q subcomponent binding protein, nuclear gene encoding mitochondrial protein. | -1,161 | 10,832 | -5,306 | 0,000 | 0,005 |
| HMBS | Homo sapiens hydroxymethylbilane synthase, trans. var. 1. | -1,168 | 8,825 | -8,579 | 0,000 | 0,001 |
| GGH | Homo sapiens gamma-glutamyl hydrolase (conjugase, folylpolygammaglutamyl hydrolase). | -1,169 | 9,283 | -3,299 | 0,009 | 0,036 |
| CPA4 | Homo sapiens carboxypeptidase A4. | -1,169 | 7,777 | -6,884 | 0,000 | 0,002 |
| PSME3 | Homo sapiens proteasome (prosome. macropain) activator subunit 3 (PA28 gamma; Ki), trans. var. 1. | -1,173 | 9,054 | -7,629 | 0,000 | 0,001 |
| ATAD2 | Homo sapiens ATPase family, AAA domain containing 2. | -1,174 | 8,827 | -7,654 | 0,000 | 0,001 |
| NME1 | Homo sapiens non-metastatic cells 1, protein (NM23A) expressed in, trans. var. 2. | -1,175 | 12,197 | -3,691 | 0,005 | 0,022 |
| PRMT6 | Homo sapiens protein arginine methyltransferase 6. | -1,175 | 8,128 | -7,850 | 0,000 | 0,001 |
| RPS15 | Homo sapiens ribosomal protein S15. | -1,176 | 9,346 | -5,134 | 0,001 | 0,005 |
| MRPL12 | Homo sapiens mitochondrial ribosomal protein L12, nuclear gene encoding mitochondrial protein. | -1,178 | 9,930 | -5,885 | 0,000 | 0,003 |
| PRPS1 | Homo sapiens phosphoribosyl pyrophosphate synthetase 1. | -1,178 | 9,336 | -5,124 | 0,001 | 0,006 |
| GEMIN6 | Homo sapiens gem (nuclear organelle) associated protein 6. | -1,179 | 8,775 | -6,188 | 0,000 | 0,002 |
| PHB | Homo sapiens prohibitin. | -1,186 | 9,388 | -5,289 | 0,000 | 0,005 |
| HNRNPD | Homo sapiens heterogeneous nuclear ribonucleoprotein D (AU-rich element RNA binding protein 1. 37kDa), trans. var. 2. | -1,188 | 11,951 | -4,495 | 0,001 | 0,010 |
| ARV1 | Homo sapiens ARV1 homolog (S. cerevisiae). | -1,189 | 8,342 | -6,265 | 0,000 | 0,002 |
| HNRNPA1 | Homo sapiens heterogeneous nuclear ribonucleoprotein A1, trans. var. 2. | -1,189 | 8,516 | -7,746 | 0,000 | 0,001 |
| ID3 | Homo sapiens inhibitor of DNA binding 3, dominant negative helix-loop-helix protein (ID3). | -1,191 | 7,882 | -4,802 | 0,001 | 0,007 |
| PLK4 | Homo sapiens polo-like kinase 4 (Drosophila). | -1,193 | 8,785 | -3,186 | 0,010 | 0,041 |
| ORC6L | Homo sapiens origin recognition complex, subunit 6 like (yeast). | -1,194 | 8,680 | -5,913 | 0,000 | 0,003 |
| GBP6 | Homo sapiens guanylate binding protein family, member 6. | -1,196 | 8,030 | -6,657 | 0,000 | 0,002 |
| KIF20B | Homo sapiens kinesin family member 20B. | -1,198 | 8,379 | -5,751 | 0,000 | 0,003 |
| TUBB | Homo sapiens tubulin β. | -1,198 | 11,431 | -5,903 | 0,000 | 0,003 |
| HNRNPA0 | Homo sapiens heterogeneous nuclear ribonucleoprotein A0. | -1,199 | 9,988 | -4,998 | 0,001 | 0,006 |
| TMEM14A | Homo sapiens transmembrane protein 14A. | -1,202 | 10,505 | -8,156 | 0,000 | 0,001 |
| GCSH | Homo sapiens glycine cleavage system protein H (aminomethyl carrier). | -1,202 | 9,504 | -5,988 | 0,000 | 0,003 |
| HSPE1 | Homo sapiens heat shock 10kDa protein 1 (chaperonin 10). | -1,205 | 9,314 | -3,184 | 0,010 | 0,041 |
| MRPL40 | Homo sapiens mitochondrial ribosomal protein L40, nuclear gene encoding mitochondrial protein. | -1,206 | 9,687 | -4,913 | 0,001 | 0,007 |
| WNT10A | Homo sapiens wingless-type MMTV integration site family, member 10A. | -1,206 | 7,860 | -5,025 | 0,001 | 0,006 |
| RPL6 | Homo sapiens ribosomal protein L6, trans. var. 1. | -1,209 | 9,605 | -7,070 | 0,000 | 0,001 |
| TTK | Homo sapiens TTK protein kinase. | -1,210 | 8,747 | -3,078 | 0,012 | 0,046 |
| HN1 | Homo sapiens hematological and neurological expressed 1, trans. var. 1. | -1,210 | 8,829 | -7,964 | 0,000 | 0,001 |
| POP5 | Homo sapiens processing of precursor 5, ribonuclease P/MRP subunit (S. cerevisiae), trans. var. 3. | -1,213 | 8,622 | -6,637 | 0,000 | 0,002 |
| LOC100133328 | PREDICTED: Homo sapiens misc_RNA, miscRNA. | -1,216 | 9,132 | -6,238 | 0,000 | 0,002 |
| C6orf173 | Homo sapiens chromosome 6 open reading frame 173. | -1,218 | 9,065 | -3,103 | 0,012 | 0,045 |
| GRWD1 | Homo sapiens glutamate-rich WD repeat containing 1. | -1,219 | 9,253 | -8,977 | 0,000 | 0,001 |
| PSMB4 | Homo sapiens proteasome (prosome, macropain) subunit β type 4. | -1,221 | 10,702 | -8,683 | 0,000 | 0,001 |
| CKLF | Homo sapiens chemokine-like factor, trans. var. 5. | -1,221 | 10,192 | -3,996 | 0,003 | 0,016 |
| APTX | Homo sapiens aprataxin, trans. var. 2. | -1,222 | 8,876 | -6,116 | 0,000 | 0,003 |
| CCDC34 | Homo sapiens coiled-coil domain containing 34, trans. var. 1. | -1,222 | 8,356 | -3,914 | 0,003 | 0,017 |
| ARHGDIB | Homo sapiens Rho GDP dissociation inhibitor (GDI) β. | -1,223 | 8,231 | -5,352 | 0,000 | 0,005 |
| LOC100134073 | PREDICTED: Homo sapiens similar to LYPDC1 protein. | -1,225 | 7,936 | -5,912 | 0,000 | 0,003 |
| CYBASC3 | Homo sapiens cytochrome b, ascorbate dependent 3. | -1,225 | 8,498 | -5,519 | 0,000 | 0,004 |
| C18orf55 | Homo sapiens chromosome 18 open reading frame 55. | -1,226 | 8,705 | -10,133 | 0,000 | 0,000 |
| TMPO | Homo sapiens thymopoietin, trans. var. 1. | -1,228 | 8,197 | -5,907 | 0,000 | 0,003 |
| LOC651697 | PREDICTED: Homo sapiens misc_RNA, miscRNA. | -1,228 | 9,709 | -4,502 | 0,001 | 0,010 |
| SOX2 | Homo sapiens SRY (sex determining region Y)-box 2. | -1,229 | 7,790 | -12,563 | 0,000 | 0,000 |
| E2F2 | Homo sapiens E2F transcription factor 2. | -1,233 | 7,715 | -14,592 | 0,000 | 0,000 |
| C16orf53 | Homo sapiens chromosome 16 open reading frame 53. | -1,233 | 9,087 | -8,216 | 0,000 | 0,001 |
| EFEMP1 | Homo sapiens EGF-containing fibulin-like extracellular matrix protein 1, trans. var. 1. | -1,233 | 8,297 | -3,336 | 0,008 | 0,034 |
| CDC25A | Homo sapiens cell division cycle 25 homolog A (S. pombe), trans. var. 1. | -1,235 | 8,071 | -8,087 | 0,000 | 0,001 |
| FAM96A | Homo sapiens family with sequence similarity 96, member A, trans. var. 1. | -1,238 | 10,255 | -9,474 | 0,000 | 0,001 |
| PPT1 | Homo sapiens palmitoyl-protein thioesterase 1 (ceroid-lipofuscinosis, neuronal 1, infantile). | -1,242 | 10,613 | -3,440 | 0,007 | 0,030 |
| TIMELESS | Homo sapiens timeless homolog (Drosophila). | -1,243 | 8,743 | -4,621 | 0,001 | 0,009 |
| APITD1 | Homo sapiens apoptosis-inducing, TAF9-like domain 1, trans. var. B. | -1,244 | 8,300 | -7,079 | 0,000 | 0,001 |
| QDPR | Homo sapiens quinoid dihydropteridine reductase. | -1,245 | 9,062 | -4,419 | 0,001 | 0,010 |
| RNF26 | Homo sapiens ring finger protein 26. | -1,247 | 8,712 | -9,758 | 0,000 | 0,000 |
| LOC653110 | PREDICTED: Homo sapiens similar to annexin A8, trans. var. 1. | -1,247 | 9,268 | -7,365 | 0,000 | 0,001 |
| ATP1B1 | Homo sapiens ATPase. Na+/K+ transporting, β1 polypeptide, trans. var. 2. | -1,248 | 10,429 | -5,089 | 0,001 | 0,006 |
| LOC100132299 | PREDICTED: Homo sapiens similar to MSTP075. | -1,248 | 9,064 | -6,800 | 0,000 | 0,002 |
| HNRNPD | Homo sapiens heterogeneous nuclear ribonucleoprotein D (AU-rich element RNA binding protein, 1. 37kDa), trans. var. 3. | -1,252 | 11,715 | -5,057 | 0,001 | 0,006 |
| ZWINT | Homo sapiens ZW10 interactor, trans. var. 3. | -1,254 | 8,097 | -8,339 | 0,000 | 0,001 |
| GALNTL4 | Homo sapiens UDP-N-acetyl-alpha-D-galactosamine:polypeptide N-acetylgalactosaminyltransferase-like 4. | -1,259 | 8,261 | -7,221 | 0,000 | 0,001 |
| PRMT6 | Homo sapiens protein arginine methyltransferase 6. | -1,260 | 8,228 | -9,769 | 0,000 | 0,000 |
| BUB1 | Homo sapiens BUB1 budding uninhibited by benzimidazoles 1 homolog (yeast). | -1,260 | 8,593 | -4,210 | 0,002 | 0,013 |
| STARD7 | Homo sapiens START domain containing 7, trans. var. 2. | -1,260 | 9,571 | -8,729 | 0,000 | 0,001 |
| KIF2C | Homo sapiens kinesin family member 2C. | -1,261 | 8,273 | -4,477 | 0,001 | 0,010 |
| CXXC5 | Homo sapiens CXXC finger 5. | -1,262 | 8,947 | -3,634 | 0,005 | 0,024 |
| CALM3 | Homo sapiens calmodulin 3 (phosphorylase kinase. delta). | -1,266 | 11,290 | -5,977 | 0,000 | 0,003 |
| DUT | Homo sapiens deoxyuridine triphosphatase, nuclear gene encoding mitochondrial protein, trans. var. 1. | -1,270 | 8,167 | -4,784 | 0,001 | 0,007 |
| C17orf97 | Homo sapiens chromosome 17 open reading frame 97. | -1,271 | 8,747 | -5,606 | 0,000 | 0,004 |
| PTDSS1 | Homo sapiens phosphatidylserine synthase 1. | -1,276 | 9,876 | -5,197 | 0,000 | 0,005 |
| RHOBTB3 | Homo sapiens Rho-related BTB domain containing 3. | -1,276 | 8,478 | -4,802 | 0,001 | 0,007 |
| ICMT | Homo sapiens isoprenylcysteine carboxyl methyltransferase. | -1,277 | 9,078 | -6,507 | 0,000 | 0,002 |
| LOC493869 | Homo sapiens similar to RIKEN cDNA 2310016C16. | -1,279 | 9,497 | -7,024 | 0,000 | 0,002 |
| MRPL13 | Homo sapiens mitochondrial ribosomal protein L13, nuclear gene encoding mitochondrial protein. | -1,282 | 10,050 | -8,221 | 0,000 | 0,001 |
| HMGB2 | Homo sapiens high-mobility group box 2. | -1,283 | 8,725 | -4,122 | 0,002 | 0,014 |
| ATP1B1 | Homo sapiens ATPase, Na+/K+ transporting β1 polypeptide, trans. var. 1. | -1,290 | 9,539 | -5,683 | 0,000 | 0,004 |
| RAD51C | Homo sapiens RAD51 homolog C (S. cerevisiae), trans. var. 2. | -1,291 | 8,613 | -5,594 | 0,000 | 0,004 |
| LSM4 | Homo sapiens LSM4 homolog, U6 small nuclear RNA associated (S. cerevisiae). | -1,297 | 10,593 | -5,123 | 0,001 | 0,006 |
| SIGMAR1 | Homo sapiens sigma non-opioid intracellular receptor 1, trans. var. 1. | -1,297 | 9,508 | -4,496 | 0,001 | 0,010 |
| RPA1 | Homo sapiens replication protein A1, 70kDa. | -1,301 | 10,418 | -5,380 | 0,000 | 0,004 |
| NEK2 | Homo sapiens NIMA (never in mitosis gene a)-related kinase 2. | -1,302 | 8,413 | -3,714 | 0,004 | 0,022 |
| CSE1L | Homo sapiens CSE1 chromosome segregation 1-like (yeast). | -1,308 | 10,816 | -4,699 | 0,001 | 0,008 |
| LY6E | Homo sapiens lymphocyte antigen 6 complex. locus E. | -1,309 | 11,431 | -3,384 | 0,007 | 0,032 |
| EXO1 | Homo sapiens exonuclease 1, trans. var. 1. | -1,311 | 8,216 | -5,698 | 0,000 | 0,004 |
| TFDP1 | Homo sapiens transcription factor Dp-1. | -1,313 | 10,334 | -8,945 | 0,000 | 0,001 |
| RPA1 | Homo sapiens replication protein A1, 70kDa. | -1,314 | 9,222 | -7,359 | 0,000 | 0,001 |
| MELK | Homo sapiens maternal embryonic leucine zipper kinase. | -1,316 | 9,650 | -3,270 | 0,009 | 0,037 |
| GMNN | Homo sapiens geminin, DNA replication inhibitor. | -1,318 | 8,397 | -11,214 | 0,000 | 0,000 |
| POLE2 | Homo sapiens polymerase (DNA directed) epsilon 2 (p59 subunit). | -1,321 | 8,064 | -11,808 | 0,000 | 0,000 |
| RTN4IP1 | Homo sapiens reticulon 4 interacting protein 1, nuclear gene encoding mitochondrial protein. | -1,324 | 8,600 | -11,572 | 0,000 | 0,000 |
| NUP37 | Homo sapiens nucleoporin, 37kDa. | -1,326 | 10,478 | -6,736 | 0,000 | 0,002 |
| MGC40489 | PREDICTED: Homo sapiens hypothetical protein MGC40489, misc RNA. | -1,326 | 9,533 | -5,069 | 0,001 | 0,006 |
| CKAP2L | Homo sapiens cytoskeleton associated protein 2-like. | -1,331 | 8,969 | -3,759 | 0,004 | 0,021 |
| RPA3 | Homo sapiens replication protein A3, 14kDa. | -1,331 | 11,303 | -3,964 | 0,003 | 0,017 |
| NCAPD2 | Homo sapiens non-SMC condensin I complex, subunit D2. | -1,334 | 8,694 | -4,147 | 0,002 | 0,014 |
| PSPH | Homo sapiens phosphoserine phosphatase. | -1,337 | 9,565 | -5,275 | 0,000 | 0,005 |
| KIF11 | Homo sapiens kinesin family member 11. | -1,343 | 8,213 | -5,734 | 0,000 | 0,003 |
| LAPTM4B | Homo sapiens lysosomal protein transmembrane 4 β. | -1,346 | 11,379 | -4,209 | 0,002 | 0,013 |
| SFRS2 | Homo sapiens splicing factor, arginine/serine-rich 2. | -1,347 | 11,722 | -17,876 | 0,000 | 0,000 |
| PYCARD | Homo sapiens PYD and CARD domain containing, trans. var. 1. | -1,348 | 9,809 | -5,409 | 0,000 | 0,004 |
| NSL1 | Homo sapiens NSL1, MIND kinetochore complex component, homolog (S. cerevisiae), trans. var. 2. | -1,349 | 8,828 | -8,433 | 0,000 | 0,001 |
| MCM7 | Homo sapiens minichromosome maintenance complex component 7, trans. var. 2. | -1,351 | 11,811 | -4,481 | 0,001 | 0,010 |
| AIMP2 | Homo sapiens aminoacyl tRNA synthetase complex-interacting multifunctional protein 2. | -1,351 | 10,887 | -4,770 | 0,001 | 0,008 |
| ANLN | Homo sapiens anillin, actin binding protein. | -1,353 | 8,770 | -3,427 | 0,007 | 0,031 |
| PRDX3 | Homo sapiens peroxiredoxin 3, nuclear gene encoding mitochondrial protein, trans. var. 1. | -1,353 | 10,146 | -4,806 | 0,001 | 0,007 |
| MSH6 | Homo sapiens mutS homolog 6 (E. coli). | -1,354 | 9,752 | -7,147 | 0,000 | 0,001 |
| BRI3BP | PREDICTED: Homo sapiens BRI3 binding protein. | -1,356 | 9,352 | -6,491 | 0,000 | 0,002 |
| TFDP1 | Homo sapiens transcription factor Dp-1. | -1,360 | 10,344 | -6,640 | 0,000 | 0,002 |
| ASPM | Homo sapiens asp (abnormal spindle) homolog, microcephaly associated (Drosophila). | -1,365 | 8,671 | -4,078 | 0,002 | 0,015 |
| ALDH7A1 | Homo sapiens aldehyde dehydrogenase 7 family, member A1. | -1,366 | 8,500 | -4,011 | 0,003 | 0,016 |
| SOX2 | Homo sapiens SRY (sex determining region Y)-box 2. | -1,373 | 7,906 | -12,490 | 0,000 | 0,000 |
| DNMT1 | Homo sapiens DNA (cytosine-5-)-methyltransferase 1. | -1,373 | 10,887 | -5,147 | 0,001 | 0,005 |
| LMNB2 | Homo sapiens lamin B2. | -1,379 | 10,088 | -3,983 | 0,003 | 0,016 |
| PRDX3 | Homo sapiens peroxiredoxin 3, nuclear gene encoding mitochondrial protein, trans. var. 1. | -1,390 | 10,448 | -5,390 | 0,000 | 0,004 |
| C9orf140 | Homo sapiens chromosome 9 open reading frame 140. | -1,390 | 8,638 | -3,087 | 0,012 | 0,046 |
| TACC3 | Homo sapiens transforming, acidic coiled-coil containing protein 3. | -1,391 | 8,648 | -4,189 | 0,002 | 0,013 |
| NUSAP1 | Homo sapiens nucleolar and spindle associated protein 1, trans. var. 2. | -1,396 | 8,356 | -4,813 | 0,001 | 0,007 |
| STARD7 | Homo sapiens START domain containing 7, trans. var. 1. | -1,403 | 11,145 | -7,876 | 0,000 | 0,001 |
| TTF2 | Homo sapiens transcription termination factor, RNA polymerase II. | -1,403 | 8,790 | -4,520 | 0,001 | 0,009 |
| KPNA2 | Homo sapiens karyopherin α-2 (RAG cohort 1, importin α 1). | -1,407 | 9,457 | -4,878 | 0,001 | 0,007 |
| LAPTM4B | Homo sapiens lysosomal protein transmembrane 4 β. | -1,413 | 11,223 | -4,050 | 0,003 | 0,015 |
| HADH | Homo sapiens hydroxyacyl-Coenzyme A dehydrogenase, nuclear gene encoding mitochondrial protein. | -1,418 | 8,553 | -4,695 | 0,001 | 0,008 |
| CENPA | Homo sapiens centromere protein A, trans. var. 2. | -1,420 | 8,680 | -3,314 | 0,008 | 0,035 |
| PRSS23 | Homo sapiens protease, serine 23. | -1,421 | 10,165 | -5,271 | 0,000 | 0,005 |
| PXMP2 | Homo sapiens peroxisomal membrane protein 2, 22kDa. | -1,433 | 9,307 | -3,573 | 0,005 | 0,026 |
| C18orf56 | Homo sapiens chromosome 18 open reading frame 56. | -1,436 | 8,683 | -4,516 | 0,001 | 0,009 |
| HMGB2 | Homo sapiens high-mobility group box 2. | -1,436 | 9,038 | -3,621 | 0,005 | 0,024 |
| MCM5 | Homo sapiens minichromosome maintenance complex component 5. | -1,437 | 8,885 | -5,664 | 0,000 | 0,004 |
| MCM10 | Homo sapiens minichromosome maintenance complex component 10, trans. var. 2. | -1,448 | 8,085 | -10,151 | 0,000 | 0,000 |
| C16orf33 | Homo sapiens chromosome 16 open reading frame 33. | -1,457 | 9,175 | -7,204 | 0,000 | 0,001 |
| IDH1 | Homo sapiens isocitrate dehydrogenase 1 (NADP+), soluble. | -1,458 | 9,705 | -5,103 | 0,001 | 0,006 |
| MCM4 | Homo sapiens minichromosome maintenance complex component 4, trans. var. 2. | -1,464 | 8,696 | -8,828 | 0,000 | 0,001 |
| C18orf55 | Homo sapiens chromosome 18 open reading frame 55. | -1,469 | 9,479 | -10,535 | 0,000 | 0,000 |
| PKMYT1 | Homo sapiens protein kinase, membrane associated tyrosine/threonine 1, trans. var. 2. | -1,470 | 8,967 | -4,048 | 0,003 | 0,015 |
| PBK | Homo sapiens PDZ binding kinase. | -1,476 | 8,486 | -4,503 | 0,001 | 0,010 |
| FH | Homo sapiens fumarate hydratase, nuclear gene encoding mitochondrial protein . | -1,477 | 10,322 | -5,133 | 0,001 | 0,005 |
| PTTG1 | Homo sapiens pituitary tumor-transforming 1. | -1,480 | 11,374 | -3,529 | 0,006 | 0,027 |
| PTTG1 | Homo sapiens pituitary tumor-transforming 1. | -1,481 | 10,578 | -3,541 | 0,006 | 0,027 |
| RANBP1 | Homo sapiens RAN binding protein 1. | -1,481 | 11,838 | -4,898 | 0,001 | 0,007 |
| CDCA3 | Homo sapiens cell division cycle associated 3 | -1,483 | 8,450 | -4,246 | 0,002 | 0,012 |
| RFC4 | Homo sapiens replication factor C (activator 1) 4, 37kDa, trans. var. 2. | -1,483 | 10,168 | -4,809 | 0,001 | 0,007 |
| NETO2 | Homo sapiens neuropilin (NRP) and tolloid (TLL)-like 2. | -1,489 | 9,715 | -4,313 | 0,002 | 0,012 |
| LOC727761 | PREDICTED: Homo sapiens similar to Deoxythymidylate kinase (thymidylate kinase) trans. var. 4. | -1,496 | 9,662 | -6,843 | 0,000 | 0,002 |
| MAT2A | Homo sapiens methionine adenosyltransferase II α. | -1,498 | 10,872 | -6,703 | 0,000 | 0,002 |
| CENPM | Homo sapiens centromere protein M, trans. var. 2. | -1,499 | 8,364 | -5,818 | 0,000 | 0,003 |
| PAICS | Homo sapiens phosphoribosylaminoimidazole carboxylase. phosphoribosylaminoimidazole succinocarboxamide synthetase, trans. var. 2. | -1,505 | 10,979 | -4,781 | 0,001 | 0,007 |
| PAICS | Homo sapiens phosphoribosylaminoimidazole carboxylase. phosphoribosylaminoimidazole succinocarboxamide synthetase, trans. var. 2. | -1,506 | 11,092 | -4,856 | 0,001 | 0,007 |
| MDH1 | Homo sapiens malate dehydrogenase 1. NAD (soluble). | -1,518 | 11,617 | -4,803 | 0,001 | 0,007 |
| COQ3 | Homo sapiens coenzyme Q3 homolog, methyltransferase (S. cerevisiae). | -1,520 | 8,698 | -6,602 | 0,000 | 0,002 |
| CDC2 | Homo sapiens cell division cycle 2, G1 to S and G2 to M, trans. var. 1. | -1,525 | 8,754 | -3,914 | 0,003 | 0,017 |
| TMEM97 | Homo sapiens transmembrane protein 97. | -1,528 | 9,481 | -3,615 | 0,005 | 0,024 |
| RAD51AP1 | Homo sapiens RAD51 associated protein 1. | -1,530 | 8,446 | -5,309 | 0,000 | 0,005 |
| STMN1 | Homo sapiens stathmin 1, trans. var. 1. | -1,533 | 9,341 | -4,940 | 0,001 | 0,006 |
| CCNB1 | Homo sapiens cyclin B1. | -1,543 | 8,450 | -4,524 | 0,001 | 0,009 |
| AURKB | Homo sapiens aurora kinase B. | -1,549 | 9,013 | -3,676 | 0,005 | 0,023 |
| WDR4 | Homo sapiens WD repeat domain 4, trans. var. 2. | -1,549 | 9,419 | -5,253 | 0,000 | 0,005 |
| C16orf75 | Homo sapiens chromosome 16 open reading frame 75. | -1,551 | 8,909 | -4,781 | 0,001 | 0,007 |
| FAM111A | Homo sapiens family with sequence similarity 111, member A, trans. var. 1. | -1,554 | 9,010 | -7,040 | 0,000 | 0,002 |
| TPX2 | Homo sapiens TPX2, microtubule-associated, homolog (Xenopus laevis). | -1,556 | 9,220 | -3,484 | 0,006 | 0,029 |
| DCTPP1 | Homo sapiens dCTP pyrophosphatase 1. | -1,556 | 11,293 | -5,206 | 0,000 | 0,005 |
| RRM2 | Homo sapiens ribonucleotide reductase M2 polypeptide. | -1,560 | 8,537 | -5,703 | 0,000 | 0,004 |
| MELK | Homo sapiens maternal embryonic leucine zipper kinase. | -1,560 | 10,159 | -3,279 | 0,009 | 0,036 |
| CCNF | Homo sapiens cyclin F. | -1,562 | 8,792 | -4,979 | 0,001 | 0,006 |
| SKA2 | Homo sapiens spindle and kinetochore associated complex subunit 2, trans. var. 1. | -1,564 | 9,351 | -7,736 | 0,000 | 0,001 |
| SMC4 | Homo sapiens structural maintenance of chromosomes 4, trans. var. 2. | -1,566 | 9,388 | -3,720 | 0,004 | 0,022 |
| PTTG3P | Homo sapiens pituitary tumor-transforming 3 (pseudogene), non-coding RNA. | -1,567 | 10,601 | -3,555 | 0,006 | 0,026 |
| NCAPG | Homo sapiens non-SMC condensin I complex. subunit G. | -1,570 | 9,131 | -3,289 | 0,009 | 0,036 |
| SNCA | Homo sapiens synuclein, α (non A4 component of amyloid precursor), trans. var. NACP112. | -1,572 | 9,072 | -5,268 | 0,000 | 0,005 |
| SUPT16H | Homo sapiens suppressor of Ty 16 homolog (S. cerevisiae). | -1,583 | 10,316 | -7,293 | 0,000 | 0,001 |
| DCTPP1 | Homo sapiens dCTP pyrophosphatase 1. | -1,599 | 10,502 | -6,033 | 0,000 | 0,003 |
| CAV1 | Homo sapiens caveolin 1, caveolae protein, 22kDa. | -1,617 | 12,828 | -3,549 | 0,006 | 0,026 |
| CDCA5 | Homo sapiens cell division cycle associated 5. | -1,630 | 9,753 | -3,324 | 0,008 | 0,034 |
| PRC1 | Homo sapiens protein regulator of cytokinesis 1, trans. var. 2. | -1,630 | 10,040 | -3,272 | 0,009 | 0,037 |
| KIFC1 | Homo sapiens kinesin family member C1. | -1,636 | 8,908 | -4,610 | 0,001 | 0,009 |
| PNPO | Homo sapiens pyridoxamine 5'-phosphate oxidase. | -1,638 | 9,260 | -5,914 | 0,000 | 0,003 |
| CCDC34 | Homo sapiens coiled-coil domain containing 34, trans. var. 1. | -1,638 | 9,619 | -3,504 | 0,006 | 0,028 |
| MCM4 | Homo sapiens minichromosome maintenance complex component 4, trans. var. 1. | -1,638 | 10,244 | -6,499 | 0,000 | 0,002 |
| NUSAP1 | Homo sapiens nucleolar and spindle associated protein 1, trans. var. 2. | -1,649 | 8,864 | -3,969 | 0,003 | 0,017 |
| DPYSL3 | Homo sapiens dihydropyrimidinase-like 3. | -1,650 | 9,288 | -5,167 | 0,000 | 0,005 |
| DLL1 | Homo sapiens delta-like 1 (Drosophila). | -1,653 | 9,640 | -6,877 | 0,000 | 0,002 |
| KIAA0101 | Homo sapiens KIAA0101, trans. var. 1. | -1,655 | 9,433 | -3,430 | 0,007 | 0,031 |
| RFC4 | Homo sapiens replication factor C (activator 1) 4, 37kDa, trans. var. 1. | -1,656 | 10,597 | -5,531 | 0,000 | 0,004 |
| HJURP | Homo sapiens Holliday junction recognition protein. | -1,658 | 8,915 | -4,026 | 0,003 | 0,016 |
| CAV1 | Homo sapiens caveolin 1, caveolae protein, 22kDa. | -1,659 | 9,841 | -3,995 | 0,003 | 0,016 |
| HMGN2 | Homo sapiens high-mobility group nucleosomal binding domain 2. | -1,666 | 11,734 | -3,101 | 0,012 | 0,045 |
| PRIM1 | Homo sapiens primase, DNA, polypeptide 1 (49kDa). | -1,672 | 8,580 | -6,913 | 0,000 | 0,002 |
| CDCA8 | Homo sapiens cell division cycle associated 8. | -1,676 | 8,975 | -4,570 | 0,001 | 0,009 |
| C1orf59 | Homo sapiens chromosome 1 open reading frame 59. | -1,683 | 9,523 | -5,758 | 0,000 | 0,003 |
| SPC24 | Homo sapiens SPC24, NDC80 kinetochore complex component, homolog (S. cerevisiae). | -1,690 | 8,601 | -6,370 | 0,000 | 0,002 |
| PRIM1 | Homo sapiens primase, DNA, polypeptide 1 (49kDa). | -1,703 | 8,594 | -7,112 | 0,000 | 0,001 |
| LOC727803 | PREDICTED: Homo sapiens misc_RNA, miscRNA. | -1,705 | 10,899 | -4,820 | 0,001 | 0,007 |
| CXXC5 | Homo sapiens CXXC finger 5. | -1,705 | 9,239 | -4,165 | 0,002 | 0,013 |
| H2AFX | Homo sapiens H2A histone family, member X. | -1,707 | 8,952 | -7,428 | 0,000 | 0,001 |
| RRM1 | Homo sapiens ribonucleotide reductase M1 polypeptide. | -1,711 | 10,200 | -3,856 | 0,003 | 0,019 |
| DLGAP5 | Homo sapiens discs, large (Drosophila) homolog-associated protein 5. | -1,712 | 8,535 | -4,856 | 0,001 | 0,007 |
| LOC647000 | PREDICTED: Homo sapiens similar to tubulin β 5. | -1,723 | 10,795 | -6,186 | 0,000 | 0,002 |
| HIST1H4C | Homo sapiens histone cluster 1, H4c. | -1,723 | 12,065 | -3,344 | 0,008 | 0,034 |
| LOC399942 | PREDICTED: Homo sapiens similar to Tubulin α-2 chain (α-tubulin 2) trans. var. 5. | -1,725 | 10,458 | -6,388 | 0,000 | 0,002 |
| SCD | Homo sapiens stearoyl-CoA desaturase (delta-9-desaturase). | -1,729 | 11,637 | -3,230 | 0,010 | 0,038 |
| FBXO5 | Homo sapiens F-box protein 5. | -1,731 | 8,629 | -7,980 | 0,000 | 0,001 |
| TK1 | Homo sapiens thymidine kinase 1, soluble. | -1,736 | 9,700 | -3,254 | 0,009 | 0,037 |
| CYR61 | Homo sapiens cysteine-rich, angiogenic inducer. 61. | -1,742 | 9,722 | -4,725 | 0,001 | 0,008 |
| DLGAP5 | Homo sapiens discs, large (Drosophila) homolog-associated protein 5. | -1,744 | 8,571 | -4,558 | 0,001 | 0,009 |
| LOC730534 | PREDICTED: Homo sapiens similar to D-PCa-2 protein isoform c. | -1,751 | 12,015 | -3,367 | 0,008 | 0,033 |
| TRMT5 | Homo sapiens TRM5 tRNA methyltransferase 5 homolog (S. cerevisiae). | -1,761 | 11,554 | -3,373 | 0,008 | 0,033 |
| LOC148915 | PREDICTED: Homo sapiens similar to Nonhistone chromosomal protein HMG-17 (High-mobility group nucleosome binding domain 2). | -1,764 | 11,158 | -3,413 | 0,007 | 0,031 |
| KRT13 | Homo sapiens keratin 13, trans. var. 2. | -1,787 | 8,371 | -3,752 | 0,004 | 0,021 |
| CCNB2 | Homo sapiens cyclin B2. | -1,831 | 9,384 | -3,164 | 0,011 | 0,042 |
| OIP5 | Homo sapiens Opa interacting protein 5. | -1,832 | 8,896 | -4,633 | 0,001 | 0,008 |
| HMMR | Homo sapiens hyaluronan-mediated motility receptor (RHAMM), trans. var. 1. | -1,838 | 8,913 | -3,761 | 0,004 | 0,021 |
| LOC729816 | PREDICTED: Homo sapiens misc_RNA, miscRNA. | -1,846 | 8,989 | -4,400 | 0,001 | 0,011 |
| RANBP1 | Homo sapiens RAN binding protein 1. | -1,847 | 11,114 | -5,062 | 0,001 | 0,006 |
| RFC5 | Homo sapiens replication factor C (activator 1) 5, 36.5kDa, trans. var. 1. | -1,872 | 9,215 | -5,182 | 0,000 | 0,005 |
| NDC80 | Homo sapiens NDC80 homolog, kinetochore complex component (S. cerevisiae). | -1,880 | 8,979 | -4,449 | 0,001 | 0,010 |
| PARP1 | Homo sapiens poly (ADP-ribose) polymerase family, member 1. | -1,886 | 10,091 | -4,427 | 0,001 | 0,010 |
| VSNL1 | Homo sapiens visinin-like 1. | -1,891 | 8,498 | -5,706 | 0,000 | 0,004 |
| ASF1B | Homo sapiens ASF1 anti-silencing function 1 homolog B (S. cerevisiae). | -1,924 | 8,549 | -6,812 | 0,000 | 0,002 |
| LOC731314 | PREDICTED: Homo sapiens similar to H2A histone family. member X. | -1,926 | 9,787 | -6,680 | 0,000 | 0,002 |
| LFNG | Homo sapiens LFNG O-fucosylpeptide 3-beta-N-acetylglucosaminyltransferase, trans. var. 1. | -1,935 | 8,313 | -3,810 | 0,004 | 0,020 |
| MCM2 | Homo sapiens minichromosome maintenance complex component 2. | -1,939 | 8,834 | -7,416 | 0,000 | 0,001 |
| CDKN3 | Homo sapiens cyclin-dependent kinase inhibitor 3 (CDK2-associated dual specificity phosphatase). | -1,958 | 9,252 | -3,611 | 0,005 | 0,025 |
| FEN1 | Homo sapiens flap structure-specific endonuclease 1. | -1,989 | 9,977 | -11,007 | 0,000 | 0,000 |
| BIRC5 | Homo sapiens baculoviral IAP repeat-containing 5, trans. var. 1. | -1,990 | 8,922 | -4,731 | 0,001 | 0,008 |
| PCNA | Homo sapiens proliferating cell nuclear antigen, trans. var. 2. | -2,005 | 9,932 | -7,196 | 0,000 | 0,001 |
| UBE2T | Homo sapiens ubiquitin-conjugating enzyme E2T (putative). | -2,046 | 9,210 | -6,244 | 0,000 | 0,002 |
| AURKA | Homo sapiens aurora kinase A, trans. var. 5. | -2,078 | 10,225 | -4,416 | 0,001 | 0,010 |
| CDCA7 | Homo sapiens cell division cycle associated 7, trans. var. 1. | -2,100 | 8,873 | -5,922 | 0,000 | 0,003 |
| CDC45L | Homo sapiens CDC45 cell division cycle 45-like (S. cerevisiae). | -2,106 | 9,509 | -4,567 | 0,001 | 0,009 |
| MAD2L1 | Homo sapiens MAD2 mitotic arrest deficient-like 1 (yeast). | -2,130 | 9,888 | -4,563 | 0,001 | 0,009 |
| SKP2 | Homo sapiens S-phase kinase-associated protein 2 (p45), trans. var. 2. | -2,139 | 9,342 | -6,972 | 0,000 | 0,002 |
| HMMR | Homo sapiens hyaluronan-mediated motility receptor (RHAMM), trans. var. 2. | -2,186 | 9,266 | -3,993 | 0,003 | 0,016 |
| AURKA | Homo sapiens aurora kinase A, trans. var. 3. | -2,205 | 10,008 | -4,701 | 0,001 | 0,008 |
| KIF20A | Homo sapiens kinesin family member 20A. | -2,230 | 8,721 | -4,983 | 0,001 | 0,006 |
| UHRF1 | Homo sapiens ubiquitin-like with PHD and ring finger domains 1, trans. var. 1. | -2,244 | 9,288 | -9,136 | 0,000 | 0,001 |
| UNG | Homo sapiens uracil-DNA glycosylase (UNG), nuclear gene encoding mitochondrial protein, trans. var. 1. | -2,303 | 9,962 | -5,156 | 0,000 | 0,005 |
| CCNA2 | Homo sapiens cyclin A2. | -2,364 | 9,825 | -3,686 | 0,005 | 0,023 |
| FAM83D | Homo sapiens family with sequence similarity 83, member D. | -2,368 | 9,120 | -5,265 | 0,000 | 0,005 |
| TYMS | Homo sapiens thymidylate synthetase. | -2,380 | 10,235 | -4,845 | 0,001 | 0,007 |
| GINS2 | Homo sapiens GINS complex subunit 2 (Psf2 homolog). | -2,393 | 9,505 | -6,083 | 0,000 | 0,003 |
| TOP2A | Homo sapiens topoisomerase (DNA) II alpha 170kDa. | -2,426 | 9,893 | -3,140 | 0,011 | 0,043 |
| MCM3 | Homo sapiens minichromosome maintenance complex component 3. | -2,484 | 11,671 | -7,280 | 0,000 | 0,001 |
| MCM3 | Homo sapiens minichromosome maintenance complex component 3. | -2,509 | 9,837 | -8,497 | 0,000 | 0,001 |
| MCM6 | Homo sapiens minichromosome maintenance complex component 6. | -2,579 | 10,055 | -7,911 | 0,000 | 0,001 |
