# Supplementary material for: Upregulation of cell cycle genes in head and neck cancer patients may be antagonized by erufosine’s down regulation of cell cycle processes in OSCC cells
Source: Oncotarget. 2017 Dec 20;9(5):5797–810. doi: 10.18632/oncotarget.23537 (PMC5814175; doi:10.18632/oncotarget.23537)
Supplement: Supplementary file 4 [file oncotarget-09-5797-s004.docx]

**Table 4A: List of GO terms enriched in IC50 vs Control**

| **GO BP ID** | **P value** | **Odds Ratio** | **Experimental Count** | **Count** | **Size** | **Term** |
| --- | --- | --- | --- | --- | --- | --- |
| GO:0006259 | 3.49774E-24 | 12.71624559 | 5.553086213 | 39 | 779 | DNA metabolic process |
| GO:0007049 | 4.72255E-22 | 9.273041796 | 10.69984904 | 48 | 1501 | cell cycle |
| GO:0006260 | 1.10302E-21 | 20.05186722 | 1.89617578 | 25 | 266 | DNA replication |
| GO:0000278 | 2.14725E-21 | 10.4301661 | 6.622358269 | 39 | 929 | mitotic cell cycle |
| GO:0006271 | 6.13113E-20 | 96.98295455 | 0.249496813 | 13 | 35 | DNA strand elongation involved in DNA replication |
| GO:0022616 | 1.46289E-19 | 88.88599537 | 0.263753774 | 13 | 37 | DNA strand elongation |
| GO:0022402 | 2.79645E-19 | 8.748066748 | 8.069439785 | 40 | 1132 | cell cycle process |
| GO:0006261 | 2.95767E-18 | 29.95153061 | 0.819775243 | 17 | 115 | DNA-dependent DNA replication |
| GO:1903047 | 5.24761E-18 | 9.504671442 | 5.517443811 | 33 | 774 | mitotic cell cycle process |
| GO:0051276 | 2.98388E-16 | 8.173370006 | 6.315833613 | 33 | 886 | chromosome organization |
| GO:0006310 | 4.61343E-15 | 16.47148468 | 1.482723918 | 18 | 208 | DNA recombination |
| GO:0006270 | 1.59108E-14 | 68.49855072 | 0.235239852 | 10 | 33 | DNA replication initiation |
| GO:0006974 | 5.48444E-13 | 7.561141728 | 4.833109695 | 26 | 678 | cellular response to DNA damage stimulus |
| GO:0006281 | 8.012E-13 | 9.531448287 | 2.958319356 | 21 | 415 | DNA repair |
| GO:0006996 | 1.13354E-12 | 4.83943979 | 21.10030191 | 52 | 2960 | organelle organization |
| GO:0044770 | 4.30968E-12 | 8.664171007 | 3.22920161 | 21 | 453 | cell cycle phase transition |
| GO:0044843 | 1.00777E-11 | 12.02380952 | 1.71083529 | 16 | 240 | cell cycle G1/S phase transition |
| GO:0071103 | 1.67133E-11 | 12.86266568 | 1.489852399 | 15 | 209 | DNA conformation change |
| GO:0000082 | 8.37139E-11 | 11.36986301 | 1.668064408 | 15 | 234 | G1/S transition of mitotic cell cycle |
| GO:0044772 | 1.4924E-10 | 7.904902389 | 3.100888963 | 19 | 435 | mitotic cell cycle phase transition |
| GO:0033260 | 1.55473E-10 | 42.31079266 | 0.263753774 | 8 | 37 | nuclear DNA replication |
| GO:0006312 | 3.04386E-10 | 38.33441558 | 0.285139215 | 8 | 40 | mitotic recombination |
| GO:0032201 | 3.15199E-10 | 58.93660969 | 0.178212009 | 7 | 25 | telomere maintenance via semi-conservative replication |
| GO:0000722 | 5.75641E-10 | 53.03397436 | 0.19246897 | 7 | 27 | telomere maintenance via recombination |
| GO:0044786 | 9.97677E-10 | 32.26520848 | 0.327910097 | 8 | 46 | cell cycle DNA replication |
| GO:1902589 | 1.00565E-09 | 4.084563615 | 14.7844683 | 39 | 2074 | single-organism organelle organization |
| GO:0000280 | 1.56521E-09 | 7.215171203 | 3.157916806 | 18 | 443 | nuclear division |
| GO:0048285 | 4.27062E-09 | 6.737129331 | 3.364642737 | 18 | 472 | organelle fission |
| GO:0032392 | 9.02486E-09 | 23.55044955 | 0.427708823 | 8 | 60 | DNA geometric change |
| GO:0010833 | 2.65283E-08 | 27.87010796 | 0.320781617 | 7 | 45 | telomere maintenance via telomere lengthening |
| GO:0071824 | 5.22209E-08 | 10.32665701 | 1.275997987 | 11 | 179 | protein-DNA complex subunit organization |
| GO:0042276 | 9.45503E-08 | 61.59895833 | 0.121184166 | 5 | 17 | error-prone translesion synthesis |
| GO:0090304 | 1.0109E-07 | 3.207676768 | 28.17175444 | 52 | 3952 | nucleic acid metabolic process |
| GO:0032508 | 1.12012E-07 | 22.04513889 | 0.392066421 | 7 | 55 | DNA duplex unwinding |
| GO:0065004 | 1.4845E-07 | 10.67853881 | 1.112042939 | 10 | 156 | protein-DNA complex assembly |
| GO:0007067 | 2.52832E-07 | 6.472676056 | 2.594766857 | 14 | 364 | mitotic nuclear division |
| GO:0034728 | 3.59941E-07 | 11.37327437 | 0.933830929 | 9 | 131 | nucleosome organization |
| GO:0033554 | 4.70034E-07 | 3.448136279 | 11.74060718 | 30 | 1647 | cellular response to stress |
| GO:0006268 | 4.88812E-07 | 97.3909465 | 0.071284804 | 4 | 10 | DNA unwinding involved in DNA replication |
| GO:0019985 | 6.237E-07 | 23.58627582 | 0.313653137 | 6 | 44 | translesion synthesis |
| GO:0006333 | 9.01952E-07 | 10.11505955 | 1.040758135 | 9 | 146 | chromatin assembly or disassembly |
| GO:0006334 | 1.01049E-06 | 12.07457889 | 0.777004361 | 8 | 109 | nucleosome assembly |
| GO:0006725 | 1.10346E-06 | 2.907021728 | 32.00687689 | 54 | 4490 | cellular aromatic compound metabolic process |
| GO:0046483 | 1.10346E-06 | 2.907021728 | 32.00687689 | 54 | 4490 | heterocycle metabolic process |
| GO:0006323 | 1.13182E-06 | 9.82474804 | 1.069272056 | 9 | 150 | DNA packaging |
| GO:0006284 | 1.2009E-06 | 20.83485428 | 0.349295538 | 6 | 49 | base-excision repair |
| GO:0006139 | 1.30104E-06 | 2.8785196 | 31.2013586 | 53 | 4377 | nucleobase-containing compound metabolic process |
| GO:0044763 | 1.33816E-06 | 5.705511631 | 62.78052667 | 80 | 8807 | single-organism cellular process |
| GO:0006301 | 1.71878E-06 | 19.47110622 | 0.37068098 | 6 | 52 | postreplication repair |
| GO:0000723 | 2.29378E-06 | 13.53172255 | 0.605920832 | 7 | 85 | telomere maintenance |
| GO:0007059 | 2.39521E-06 | 7.720066335 | 1.504109359 | 10 | 211 | chromosome segregation |
| GO:0031497 | 2.52706E-06 | 10.59198193 | 0.876803086 | 8 | 123 | chromatin assembly |
| GO:0032200 | 2.68455E-06 | 13.1911859 | 0.620177793 | 7 | 87 | telomere organization |
| GO:0016043 | 2.80474E-06 | 2.795549868 | 33.8175109 | 55 | 4744 | cellular component organization |
| GO:1901360 | 3.66776E-06 | 2.752022719 | 33.09753438 | 54 | 4643 | organic cyclic compound metabolic process |
| GO:0006297 | 4.10023E-06 | 48.67078189 | 0.114055686 | 4 | 16 | nucleotide-excision repair. DNA gap filling |
| GO:0051301 | 6.63514E-06 | 4.536516854 | 3.913535726 | 15 | 549 | cell division |
| GO:0071840 | 6.92747E-06 | 2.679106029 | 34.68005703 | 55 | 4865 | cellular component organization or biogenesis |
| GO:0051052 | 7.54228E-06 | 6.729855072 | 1.71083529 | 10 | 240 | regulation of DNA metabolic process |
| GO:0071897 | 7.78504E-06 | 11.09419703 | 0.727104998 | 7 | 102 | DNA biosynthetic process |
| GO:0070987 | 8.5908E-06 | 38.92674897 | 0.135441127 | 4 | 19 | error-free translesion synthesis |
| GO:0051321 | 1.05138E-05 | 8.619692364 | 1.062143576 | 8 | 149 | meiotic cell cycle |
| GO:0006302 | 1.15932E-05 | 8.497684134 | 1.076400537 | 8 | 151 | double-strand break repair |
