# Supplementary material for: Upregulation of cell cycle genes in head and neck cancer patients may be antagonized by erufosine’s down regulation of cell cycle processes in OSCC cells
Source: Oncotarget. 2017 Dec 20;9(5):5797–810. doi: 10.18632/oncotarget.23537 (PMC5814175; doi:10.18632/oncotarget.23537)
Supplement: Supplementary file 5 [file oncotarget-09-5797-s005.docx]

**Table 4B: List of GO terms enriched in IC75 vs Control**

| **GO BP ID** | **P-value** | **Odds Ratio** | **Experimental Count** | **Count** | **Size** | **Term** |
| --- | --- | --- | --- | --- | --- | --- |
| GO:0000278 | 4.22234E-44 | 7.136584261 | 23.91839987 | 108 | 929 | mitotic cell cycle |
| GO:1903047 | 9.25996E-44 | 7.715512821 | 19.92770882 | 99 | 774 | mitotic cell cycle process |
| GO:0022402 | 3.36203E-38 | 5.877371125 | 29.14491781 | 111 | 1132 | cell cycle process |
| GO:0007049 | 7.26442E-35 | 4.967016628 | 38.64533714 | 123 | 1501 | cell cycle |
| GO:0007067 | 2.16161E-26 | 7.785005738 | 9.371687353 | 54 | 364 | mitotic nuclear division |
| GO:0000280 | 2.67692E-26 | 6.959288894 | 11.40565247 | 59 | 443 | nuclear division |
| GO:0048285 | 7.85952E-25 | 6.453917051 | 12.15229789 | 59 | 472 | organelle fission |
| GO:0044770 | 3.95197E-24 | 6.460575758 | 11.6631164 | 57 | 453 | cell cycle phase transition |
| GO:0051301 | 1.07717E-23 | 5.783514227 | 14.13477021 | 62 | 549 | cell division |
| GO:0044772 | 2.19765E-23 | 6.453999582 | 11.19968131 | 55 | 435 | mitotic cell cycle phase transition |
| GO:0006260 | 1.14104E-21 | 8.061084906 | 6.848540758 | 42 | 266 | DNA replication |
| GO:0007059 | 8.73856E-21 | 9.012154108 | 5.432489098 | 37 | 211 | chromosome segregation |
| GO:0051276 | 2.56611E-20 | 4.22614643 | 22.81130493 | 74 | 886 | chromosome organization |
| GO:0006271 | 4.60087E-20 | 42.49949115 | 0.901123784 | 18 | 35 | DNA strand elongation involved in DNA replication |
| GO:0006259 | 1.17167E-19 | 4.364222705 | 20.05644079 | 68 | 779 | DNA metabolic process |
| GO:0022616 | 1.71043E-19 | 38.01930432 | 0.952616572 | 18 | 37 | DNA strand elongation |
| GO:0006996 | 2.48877E-19 | 2.914597815 | 76.20932573 | 148 | 2960 | organelle organization |
| GO:1902589 | 6.31227E-19 | 3.08370933 | 53.3980208 | 118 | 2074 | single-organism organelle organization |
| GO:0000819 | 2.20971E-18 | 12.85786563 | 2.806356927 | 26 | 109 | sister chromatid segregation |
| GO:0098813 | 2.32293E-17 | 10.12651701 | 3.655987924 | 28 | 142 | nuclear chromosome segregation |
| GO:0006261 | 1.10519E-16 | 11.35441292 | 2.96083529 | 25 | 115 | DNA-dependent DNA replication |
| GO:0000070 | 1.35122E-16 | 12.07800026 | 2.703371352 | 24 | 105 | mitotic sister chromatid segregation |
| GO:0044843 | 3.3168E-16 | 6.898787297 | 6.179134519 | 34 | 240 | cell cycle G1/S phase transition |
| GO:0000082 | 1.06734E-15 | 6.840396557 | 6.024656156 | 33 | 234 | G1/S transition of mitotic cell cycle |
| GO:0071103 | 9.78324E-14 | 6.628157474 | 5.38099631 | 29 | 209 | DNA conformation change |
| GO:0006270 | 6.12708E-13 | 25.63962585 | 0.849630996 | 13 | 33 | DNA replication initiation |
| GO:0051726 | 4.14248E-12 | 3.19234406 | 21.83294197 | 58 | 848 | regulation of cell cycle |
| GO:0006807 | 7.65825E-12 | 2.218588187 | 134.4219222 | 193 | 5221 | nitrogen compound metabolic process |
| GO:0010564 | 9.19428E-12 | 3.943325132 | 12.30677625 | 41 | 478 | regulation of cell cycle process |
| GO:0000226 | 1.25934E-11 | 4.55767749 | 8.831013083 | 34 | 343 | microtubule cytoskeleton organization |
| GO:0016043 | 2.52344E-11 | 2.160302574 | 122.1408923 | 179 | 4744 | cellular component organization |
| GO:0071840 | 2.96657E-11 | 2.155862481 | 125.256206 | 182 | 4865 | cellular component organization or biogenesis |
| GO:0007051 | 8.11282E-11 | 8.001388889 | 2.935088896 | 19 | 114 | spindle organization |
| GO:0034641 | 9.57461E-11 | 2.113260559 | 128.7062227 | 184 | 4999 | cellular nitrogen compound metabolic process |
| GO:0006310 | 1.29395E-10 | 5.539084603 | 5.355249916 | 25 | 208 | DNA recombination |
| GO:0007052 | 1.6673E-10 | 10.99980974 | 1.776501174 | 15 | 69 | mitotic spindle organization |
| GO:0000086 | 1.695E-10 | 5.988777828 | 4.582858101 | 23 | 178 | G2/M transition of mitotic cell cycle |
| GO:0007088 | 1.75179E-10 | 7.598020833 | 3.063820865 | 19 | 119 | regulation of mitotic nuclear division |
| GO:0007017 | 1.9027E-10 | 3.66198568 | 12.43550822 | 39 | 483 | microtubule-based process |
| GO:0044839 | 2.38229E-10 | 5.87352915 | 4.660097283 | 23 | 181 | cell cycle G2/M phase transition |
| GO:0006323 | 2.39009E-10 | 6.538949423 | 3.861959074 | 21 | 150 | DNA packaging |
| GO:0006974 | 3.99753E-10 | 3.147275387 | 17.45605502 | 47 | 678 | cellular response to DNA damage stimulus |
| GO:0007346 | 4.27234E-10 | 3.764173096 | 11.12244213 | 36 | 432 | regulation of mitotic cell cycle |
| GO:0051302 | 5.00122E-10 | 4.953870316 | 6.153388125 | 26 | 239 | regulation of cell division |
| GO:0031145 | 8.20658E-10 | 7.953103448 | 2.62613217 | 17 | 102 | anaphase-promoting complex-dependent proteasomal ubiquitin-dependent protein catabolic process |
| GO:0033260 | 1.29802E-09 | 16.56717775 | 0.952616572 | 11 | 37 | nuclear DNA replication |
| GO:0051783 | 1.40914E-09 | 6.598369565 | 3.450016773 | 19 | 134 | regulation of nuclear division |
| GO:0050000 | 2.0814E-09 | 13.08587571 | 1.235826904 | 12 | 48 | chromosome localization |
| GO:0051303 | 2.0814E-09 | 13.08587571 | 1.235826904 | 12 | 48 | establishment of chromosome localization |
| GO:0006281 | 2.11428E-09 | 3.672848586 | 10.68475344 | 34 | 415 | DNA repair |
| GO:0006725 | 2.4377E-09 | 1.985681107 | 115.6013083 | 166 | 4490 | cellular aromatic compound metabolic process |
| GO:0032201 | 6.28945E-09 | 21.89786074 | 0.643659846 | 9 | 25 | telomere maintenance via semi-conservative replication |
| GO:0065003 | 8.86731E-09 | 2.421291679 | 31.53933244 | 65 | 1225 | macromolecular complex assembly |
| GO:0046483 | 9.59533E-09 | 1.932895583 | 115.6013083 | 164 | 4490 | heterocycle metabolic process |
| GO:0034622 | 1.29685E-08 | 2.851405274 | 18.15120765 | 45 | 705 | cellular macromolecular complex assembly |
| GO:0000722 | 1.37882E-08 | 19.4614094 | 0.695152633 | 9 | 27 | telomere maintenance via recombination |
| GO:0044786 | 1.64703E-08 | 12.29749035 | 1.184334116 | 11 | 46 | cell cycle DNA replication |
| GO:0065004 | 1.84685E-08 | 5.528183293 | 4.016437437 | 19 | 156 | protein-DNA complex assembly |
| GO:0006336 | 2.66437E-08 | 11.63075237 | 1.235826904 | 11 | 48 | DNA replication-independent nucleosome assembly |
| GO:0034724 | 2.66437E-08 | 11.63075237 | 1.235826904 | 11 | 48 | DNA replication-independent nucleosome organization |
| GO:0008152 | 3.14087E-08 | 2.362403049 | 229.4776082 | 268 | 8913 | metabolic process |
| GO:0032392 | 3.17022E-08 | 9.804237288 | 1.54478363 | 12 | 60 | DNA geometric change |
| GO:0071824 | 3.40606E-08 | 5.021804396 | 4.608604495 | 20 | 179 | protein-DNA complex subunit organization |
| GO:0006139 | 3.44846E-08 | 1.884524816 | 112.6919658 | 159 | 4377 | nucleobase-containing compound metabolic process |
| GO:0051310 | 3.62708E-08 | 13.45408104 | 1.004109359 | 10 | 39 | metaphase plate congression |
| GO:1901360 | 3.85616E-08 | 1.8775871 | 119.5405065 | 166 | 4643 | organic cyclic compound metabolic process |
| GO:0051983 | 4.33226E-08 | 8.37671462 | 1.905233143 | 13 | 74 | regulation of chromosome segregation |
| GO:0006312 | 4.72691E-08 | 13.00448934 | 1.029855753 | 10 | 40 | mitotic recombination |
| GO:0071897 | 4.76858E-08 | 6.807982995 | 2.62613217 | 15 | 102 | DNA biosynthetic process |
| GO:0070507 | 6.22636E-08 | 6.653840234 | 2.677624958 | 15 | 104 | regulation of microtubule cytoskeleton organization |
| GO:0043933 | 6.60092E-08 | 2.043053691 | 53.47525998 | 91 | 2077 | macromolecular complex subunit organization |
| GO:0051304 | 9.83535E-08 | 10.00267128 | 1.390305267 | 11 | 54 | chromosome separation |
| GO:0051225 | 1.15626E-07 | 8.551248074 | 1.725008386 | 12 | 67 | spindle assembly |
| GO:0032508 | 1.20153E-07 | 9.774493243 | 1.416051661 | 11 | 55 | DNA duplex unwinding |
| GO:0032886 | 1.39908E-07 | 5.751702593 | 3.244045622 | 16 | 126 | regulation of microtubule-based process |
| GO:0000075 | 1.4666E-07 | 4.172811175 | 5.998909762 | 22 | 233 | cell cycle checkpoint |
| GO:0010833 | 1.58708E-07 | 11.14189514 | 1.158587722 | 10 | 45 | telomere maintenance via telomere lengthening |
| GO:0000723 | 2.372E-07 | 7.090183296 | 2.188443475 | 13 | 85 | telomere maintenance |
| GO:0044237 | 2.39244E-07 | 1.974589342 | 201.6715029 | 242 | 7833 | cellular metabolic process |
| GO:0071822 | 2.95056E-07 | 2.138688818 | 36.86883596 | 68 | 1432 | protein complex subunit organization |
| GO:0032200 | 3.13705E-07 | 6.897361647 | 2.239936263 | 13 | 87 | telomere organization |
| GO:0044710 | 4.05052E-07 | 1.792251686 | 106.0493962 | 148 | 4119 | single-organism metabolic process |
| GO:0090068 | 5.56038E-07 | 4.146704413 | 5.458235491 | 20 | 212 | positive regulation of cell cycle process |
| GO:0045787 | 6.72542E-07 | 3.652400514 | 7.080258303 | 23 | 275 | positive regulation of cell cycle |
| GO:0006334 | 7.43012E-07 | 5.795149991 | 2.806356927 | 14 | 109 | nucleosome assembly |
| GO:0044238 | 1.07443E-06 | 1.880630448 | 199.6890305 | 238 | 7756 | primary metabolic process |
| GO:0007080 | 1.08205E-06 | 12.92419175 | 0.823884602 | 8 | 32 | mitotic metaphase plate congression |
| GO:1901987 | 1.25846E-06 | 3.628389155 | 6.79704797 | 22 | 264 | regulation of cell cycle phase transition |
| GO:0034728 | 1.33229E-06 | 5.09314478 | 3.372777591 | 15 | 131 | nucleosome organization |
| GO:0051321 | 1.40637E-06 | 4.74753895 | 3.83621268 | 16 | 149 | meiotic cell cycle |
| GO:0043486 | 1.4187E-06 | 9.994055609 | 1.132841328 | 9 | 44 | histone exchange |
| GO:1902850 | 1.4187E-06 | 9.994055609 | 1.132841328 | 9 | 44 | microtubule cytoskeleton organization involved in mitosis |
| GO:0030261 | 1.66344E-06 | 15.92156863 | 0.617913452 | 7 | 24 | chromosome condensation |
| GO:1901990 | 1.70718E-06 | 3.684267275 | 6.385105669 | 21 | 248 | regulation of mitotic cell cycle phase transition |
| GO:0010948 | 2.01043E-06 | 3.785299486 | 5.92167058 | 20 | 230 | negative regulation of cell cycle process |
| GO:0034508 | 2.10719E-06 | 9.452203882 | 1.184334116 | 9 | 46 | centromere complex assembly |
| GO:0061640 | 2.26364E-06 | 11.48519757 | 0.901123784 | 8 | 35 | cytoskeleton-dependent cytokinesis |
| GO:0071704 | 2.45844E-06 | 1.861298108 | 205.6364475 | 242 | 7987 | organic substance metabolic process |
| GO:0006268 | 2.48222E-06 | 38.45033113 | 0.257463938 | 5 | 10 | DNA unwinding involved in DNA replication |
| GO:0042276 | 2.7023E-06 | 21.03171247 | 0.437688695 | 6 | 17 | error-prone translesion synthesis |
| GO:0000281 | 3.02448E-06 | 14.24315789 | 0.66940624 | 7 | 26 | mitotic cytokinesis |
| GO:0031497 | 3.23511E-06 | 5.044681717 | 3.166806441 | 14 | 123 | chromatin assembly |
| GO:0034080 | 3.55048E-06 | 10.69126975 | 0.952616572 | 8 | 37 | CENP-A containing nucleosome assembly |
| GO:0061641 | 3.55048E-06 | 10.69126975 | 0.952616572 | 8 | 37 | CENP-A containing chromatin organization |
| GO:0006284 | 3.67169E-06 | 8.74102349 | 1.261573298 | 9 | 49 | base-excision repair |
| GO:0051186 | 3.74558E-06 | 3.371848853 | 7.260483059 | 22 | 282 | cofactor metabolic process |
| GO:0006461 | 4.1881E-06 | 2.172061849 | 25.8751258 | 50 | 1005 | protein complex assembly |
| GO:0070271 | 4.1881E-06 | 2.172061849 | 25.8751258 | 50 | 1005 | protein complex biogenesis |
| GO:0007076 | 4.45492E-06 | 32.03918322 | 0.283210332 | 5 | 11 | mitotic chromosome condensation |
| GO:0090304 | 5.03516E-06 | 1.693386035 | 101.7497484 | 139 | 3952 | nucleic acid metabolic process |
| GO:0051656 | 5.19418E-06 | 3.408191808 | 6.848540758 | 21 | 266 | establishment of organelle localization |
| GO:0006333 | 5.22881E-06 | 4.504078218 | 3.758973499 | 15 | 146 | chromatin assembly or disassembly |
| GO:0031055 | 5.40993E-06 | 9.999784227 | 1.004109359 | 8 | 39 | chromatin remodeling at centromere |
| GO:0090307 | 5.40993E-06 | 9.999784227 | 1.004109359 | 8 | 39 | mitotic spindle assembly |
| GO:0007126 | 5.85147E-06 | 5.144437573 | 2.883596109 | 13 | 112 | meiotic nuclear division |
| GO:0010965 | 7.23557E-06 | 7.943639414 | 1.364558873 | 9 | 53 | regulation of mitotic sister chromatid separation |
| GO:0040001 | 7.92793E-06 | 16.52064547 | 0.514927877 | 6 | 20 | establishment of mitotic spindle localization |
| GO:0051640 | 9.27329E-06 | 2.993904594 | 8.856759477 | 24 | 344 | organelle localization |
| GO:0044711 | 1.00332E-05 | 1.981953621 | 33.57329755 | 59 | 1304 | single-organism biosynthetic process |
| GO:1903046 | 1.04343E-05 | 4.847942987 | 3.038074472 | 13 | 118 | meiotic cell cycle process |
| GO:0006732 | 1.04759E-05 | 3.482172711 | 6.050402549 | 19 | 235 | coenzyme metabolic process |
| GO:0045931 | 1.05173E-05 | 5.26893925 | 2.600385777 | 12 | 101 | positive regulation of mitotic cell cycle |
| GO:0031109 | 1.11673E-05 | 6.485409652 | 1.802247568 | 10 | 70 | microtubule polymerization or depolymerization |
| GO:0033045 | 1.15591E-05 | 7.434670855 | 1.441798054 | 9 | 56 | regulation of sister chromatid segregation |
| GO:0033047 | 1.15591E-05 | 7.434670855 | 1.441798054 | 9 | 56 | regulation of mitotic sister chromatid segregation |
| GO:0051306 | 1.16587E-05 | 8.853893932 | 1.107094935 | 8 | 43 | mitotic sister chromatid separation |
